# Capsaicin for Orofacial Pain Management: Systematic Review and Meta-Analysis

**DOI:** 10.3390/ijms27167099

**Published:** 2026-08-07

**Authors:** Ana Claudia de Macedo Andrade, Fernanda Aragão Felix, Sebastián Brauchi, Bruna Benso

**Affiliations:** 1 Faculty of Medicine, School of Dentistry, Pontificia Universidad Católica de Chile, Santiago 8331150, Chile; ana.demacedo@uc.cl; 2 School of Dentistry, Federal University of Minas Gerais, Belo Horizonte 31270-901, Brazil; fafelix@ufmg.br; 3 Institute of Physiology, Faculty of Medicine, Universidad Austral de Chile, Valdivia 5090000, Chile; sbrauchi@uach.cl

**Keywords:** receptors, vanilloid, capsaicin, neuropathic pain, pain, systematic reviews, PRISMA

## Abstract

Orofacial pain is a multifactorial condition that severely impacts basic functions and overall quality of life in patients, underscoring the need for non-opioid therapeutic strategies. Targeting the Transient Receptor Potential Vanilloid 1 (TRPV1) pathway has emerged as a biologically plausible approach, given its central role in nociceptive transduction and peripheral sensitization. Capsaicin, a selective TRPV1 agonist, induces receptor desensitization and has shown potential in chronic pain modulation; however, its efficacy in orofacial conditions remains unclear. This systematic review and meta-analysis aimed to evaluate the efficacy and safety of capsaicin in managing orofacial pain, compared to placebo or other pharmacological interventions. Following PRISMA guidelines, a comprehensive search of five databases (PubMed, Scopus, Web of Science, CDSR, and LILACS) was conducted without language or date restrictions. Studies involving human participants with orofacial pain treated with capsaicin were included. The protocol was registered in PROSPERO (CRD420251004538). Risk of bias was assessed using the RoB2, RoB2 crossover trial and ROBINS-I checklist, and meta-analyses were performed using random-effects models. Nine studies enrolling a total of 164 participants met the eligibility criteria and encompassed temporomandibular disorders, burning mouth syndrome, oral mucositis, trigeminal neuralgia, and neuropathic facial pain. Although placebo-controlled comparisons showed a trend toward pain reduction that did not reach statistical significance (MD = –1.87; [95% CI: –3.94, 0.19]; *p* = 0.08; I^2^ = 86%), and no significant difference was found between capsaicin concentrations (MD = 0.46; [95% CI: –2.67, 3.60]; *p* = 0.77, I^2^ = 66%), pre–post analyses of uncontrolled (single-arm) studies demonstrated a significant and clinically meaningful reduction in pain following capsaicin treatment (MD = –6.39; [95% CI: –7.40, –5.38; *p* < 0.001, I^2^ = 0%). Capsaicin also showed an acceptable safety profile: although adverse events were significantly more frequent than with control (OR = 19.84; [95% CI: 4.32–91.21]; *p* < 0.001, I^2^ = 0%), these were mild, localized, and self-limited. Capsaicin may provide clinically meaningful pain relief in selected orofacial conditions, particularly burning mouth syndrome, where uncontrolled evidence was most consistent (I^2^ = 0%); evidence for temporomandibular disorders was more heterogeneous and did not reach significance in pooled placebo-controlled analyses.

## 1. Introduction

Orofacial pain is a subjective, multifactorial phenomenon influenced by biological, psychological, and social factors, and related to actual or potential tissue damage [1]. Substantial evidence indicates that orofacial pain represents one of the most widespread health problems that negatively affects daily living activities. Ranging from incapacitating pain to an increased feeling of fatigue, orofacial pain reduces the quality of life [2,3]. The International Classification of Orofacial Pain (ICOP) covers seven main categories that affect orofacial region, including dentoalveolar and anatomically related tissues, muscle pain, temporomandibular joint (TMJ) pain, and neuropathic and idiopathic pain [4,5]. A definitive diagnosis of orofacial pain necessitates an exhaustive clinical assessment, encompassing onset, relieving and aggravating factors, as well as associated symptoms [6]. Prior epidemiological research has shown that the prevalence of acute and chronic orofacial pain differs markedly to pain subtype and age group, with reported rates ranging from 1.1% for burning mouth syndrome (BMS) to 80.8% for cancer therapy-related orofacial pain in adults [6,7].

The perception of painful stimuli involves the detection and transduction of nociceptive signals, in which transient receptor potential (TRP) channels play a major role [8].These pore-forming membrane proteins act as non-selective cation channels, mediating ion influx in response to various noxious stimuli [9,10]. A promising target for pain control is the ligand-gated ion channel transient receptor potential vanilloid 1 (TRPV1) [11]. It plays a key role in the detection of noxious heat and inflammatory pain, is predominantly expressed in Aδ and C-fiber primary afferent neurons and can be modulated by both agonists and antagonists such as capsaicin that inhibit pathological pain pathways [12,13]. Julius, Caterina, and colleagues’ landmark discovery of TRPV1 as a molecular sensor for capsaicin and noxious heat underscored the essential function of TRP channels in nociceptive signaling and as therapeutic targets for pain modulation [14,15,16]. Given the diffuse use of capsaicin as a TRPV1-targeted therapy, this review specifically focuses on capsaicin-mediated modulation of pain.

Capsaicin is the active compound of hot chili peppers and causes enhanced sensitivity, usually perceived as itching, pricking, or burning. The initial excitatory response is followed by a long-lasting refractory period with reduced sensitivity and persistent desensitization, also referred to as nociceptor inactivation [17,18]. Research efforts have employed capsaicin to manage pain associated with both local and systemic conditions, including postherpetic neuralgia [19,20], diabetic neuropathy [21], and osteoarthritis [22], demonstrating both efficacy and safety when properly administered. Capsaicin formulations differ significantly according to the intended route of administration, target condition, concentration of the drug and desired therapeutic effect, containing topical and systemic forms include hydrogels, patches, lotion, nanoemulsions, and microemulsions [23,24]. In the orofacial region, capsaicin’s topical preparations and patches have been explored as a local therapeutic agent for several conditions, such as burning mouth syndrome (BMS) [25,26], temporomandibular disorders (TMD) [27,28], and trigeminal neuralgia [29,30]. However, there is a lack of comprehensive reviews on the effectiveness of capsaicin in the management of orofacial region [31]. Accordingly, here we present a systematic review and comprehensive meta-analysis, conducted to rigorously appraise the therapeutic efficacy and safety of topical capsaicin in the management of orofacial pain, compared to placebo or alternative pharmacologic interventions.

## 2. Materials and Methods

### 2.1. Focused Question

The research question guiding this systematic review was structured according to the PICO framework (Population, Intervention, Comparison, Outcome) as follows: Is capsaicin (I) more effective and better tolerated (O) than placebo or other pharmacological intervention (C) in managing orofacial pain (P) in humans?

### 2.2. Search Strategy

**Figure 1:**
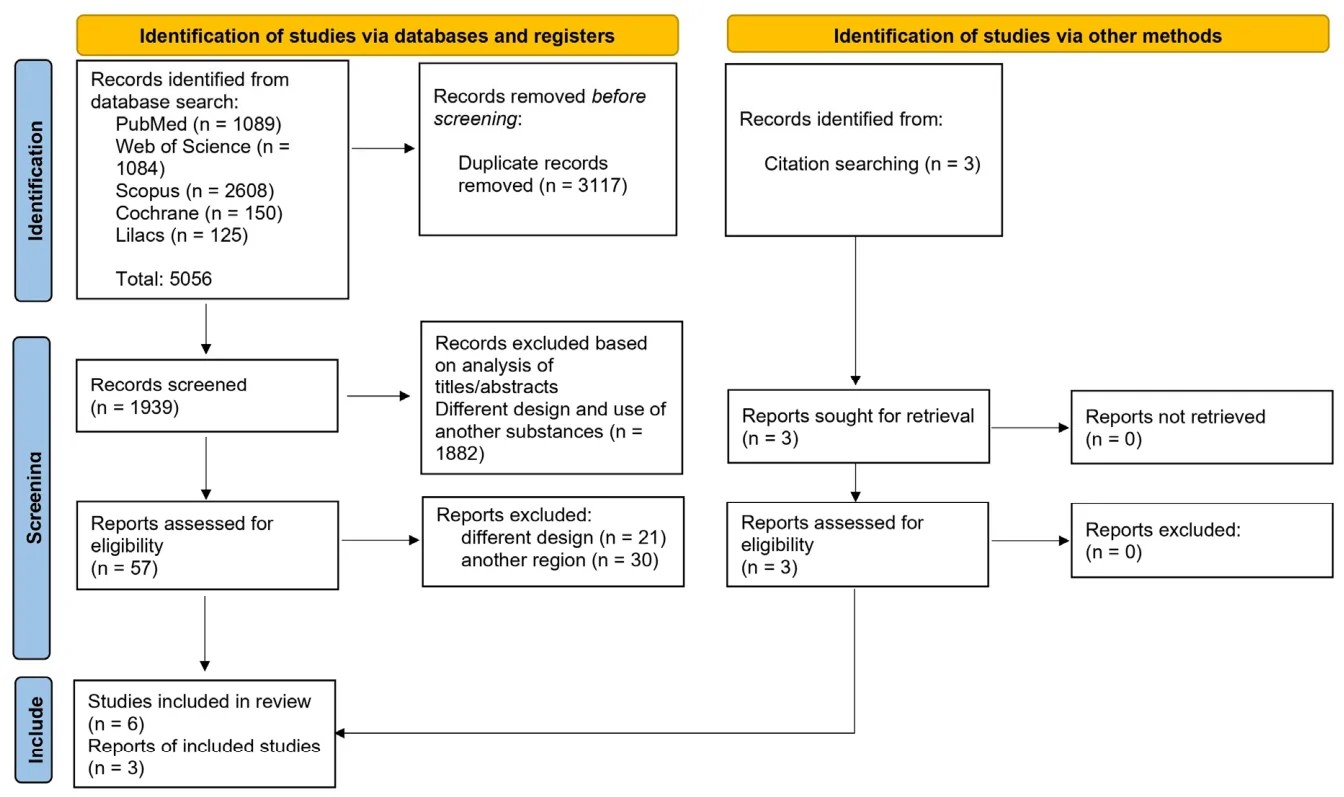
Flow diagram of literature searches according to the PRISMA statement.

This study was designed following the guidelines of the Preferred Reporting Items for Systematic Reviews and Meta-Analyses: The PRISMA Statement (2020) [32]. The protocol was registered in the International Reporting items for Systematic Reviews (PROSPERO) on 4 March 2025, under number CRD420251004538. A comprehensive systematic search was performed across the following electronic databases: PubMed (MEDLINE), Scopus, Web of Science Core Collection, Cochrane Database of Systematic Reviews (CDSR), and Latin American and Caribbean Health Sciences Literature (LILACS). The search strategy employed a combination of Medical Subject Headings (MeSH) and free-text terms targeting titles and abstracts, including: ‘Facial Pain,’ ‘Facial Neuralgia,’ ‘Acute Pain,’ ‘Chronic Pain,’ ‘Eye Pain,’ ‘TRPV Cation Channels,’ and ‘Capsaicin.’ Boolean operators “AND” and “OR” were used to optimize the combination of search terms (Supplementary File S1). No language or publication year restrictions were applied to the bibliographic search. To ensure exhaustive retrieval, reference lists of all eligible studies were systematically reviewed to identify additional relevant publications beyond those retrieved through database searches. The study selection process was summarized in the PRISMA flow diagram (Figure 1).

### 2.3. Eligibility Criteria

Studies qualified for inclusion if they met the following criteria (i) clinical trials, cross-sectional, cohort studies, or case–control studies; (ii) studies including patients diagnosed with orofacial pain; (iii) studies evaluating the efficacy of capsaicin; (iv) human studies. The following studies were excluded: (i) case reports, case series, in vitro studies, literature reviews, letters to the editor, and those not meeting the inclusion criteria; and (ii) studies assessing antinociceptive agents other than capsaicin.

### 2.4. Screening and Selection of Studies

A comprehensive literature search was initially conducted by a designated reviewer to identify all relevant studies. Subsequently, two independent reviewers screened the titles and abstracts to select potentially eligible articles. Additionally, during the full-text review of the articles selected from the databases, a manual search of the reference lists was conducted. Studies identified through this process that involved “capsaicin and clinical trials” were included in the screening and eligibility assessment. In the final screening phase, full-text articles that did not meet the inclusion criteria were excluded. The remaining studies were selected for inclusion in qualitative synthesis.

### 2.5. Data Extraction

Two reviewers independently collected relevant data from all studies meeting the eligibility criteria using a pre-defined and standardized form. The following variables were recorded: (1) first author and year of publication; (2) geographic location of the study; (3) study design; (4) duration of follow-up; (5) subtype and anatomical site of orofacial pain; (6) total sample size; (7) participant age range; (8) sex distribution; (9) intervention details, including the number and proportion of responders; (10) capsaicin concentration; (11) primary and secondary outcomes; (12) route of administration; and (13) reported adverse events or safety outcomes. Any discrepancies were resolved through consensus, with adjudication by a third reviewer when required.

### 2.6. Risk of Bias Assessment

The risk of bias of the included studies was assessed according to study design using validated tools recommended by the Cochrane Collaboration. Randomized controlled trials were evaluated using the revised Cochrane Risk of Bias tool for randomized trials (RoB 2) (Supplementary File S2; Supplementary File S3) whereas non-randomized intervention studies were assessed using the Risk Of Bias In Non-randomized Studies of Interventions (ROBINS-I) tool (Supplementary File S4).

The RoB 2 tool evaluates five domains of bias: (i) bias arising from the randomization process, (ii) bias due to deviations from intended interventions, (iii) bias due to missing outcome data, (iv) bias in measurement of the outcome, and (v) bias in selection of the reported result. Each domain was judged as low risk of bias, some concerns, or high risk of bias, leading to an overall risk-of-bias judgment for each outcome. The ROBINS-I tool evaluates seven domains: (i) bias due to confounding, (ii) bias in selection of participants into the study, (iii) bias in classification of interventions, (iv) bias due to deviations from intended interventions, (v) bias due to missing data, (vi) bias in measurement of outcomes, and (vii) bias in selection of the reported result. Each domain and the overall study were judged as presenting low, moderate, serious, or critical risk of bias, or no information, according to the ROBINS-I guidance. Risk-of-bias assessments were performed independently by two reviewers. Any disagreements were resolved through discussion until consensus was reached.

### 2.7. Certainty of Evidence Assessment

The certainty of the evidence for each outcome included in the meta-analysis was assessed using the Grading of Recommendations Assessment, Development and Evaluation (GRADE) approach. The certainty of the evidence was evaluated across the domains of risk of bias, inconsistency, indirectness, imprecision, and publication bias. Randomized controlled trials were initially rated as high-certainty evidence and could be downgraded according to these domains, whereas non-randomized studies were considered separately because they were not included in the comparative Summary of Findings analysis. The certainty of the evidence was categorized as high, moderate, low, or very low. The GRADE assessments and Summary of Findings table were generated using GRADEpro Guideline Development Tool (GRADEpro GDT; McMaster University and Evidence Prime, Hamilton, Canada).

### 2.8. Data Analysis

Outcome data from studies selected for meta-analysis were entered into and analyzed using Review Manager (RevMan) software. (version 5.4; The Nordic Cochrane Centre, The Cochrane Collaboration, Copenhagen, Denmark, 2014). Prior to synthesis, data were checked for completeness. When summary statistics such as standard deviations or standard errors were not reported, authors were contacted to request missing data. Studies that did not provide extractable outcome data were included in the qualitative synthesis only and excluded from the meta-analysis. No unit conversions were required, as all included studies reported pain outcomes on comparable numerical scales. Four different outcomes were evaluated: (1) The effect of capsaicin on reducing orofacial pain in controlled studies (continuous outcome); (2) the effect of capsaicin on orofacial pain reduction before and after treatment in uncontrolled (single-arm) studies (continuous outcome); (3) duration of capsaicin use and its effect on orofacial pain reduction (continuous outcome); (4) the adverse events associated with capsaicin use were analyzed as a dichotomous outcome. Pooled estimates were calculated using the Mantel–Haenszel method under a random-effects model, which accounts for potential between-study variability. The measure of association was expressed as odds ratios (ORs) with 95% confidence intervals (CIs). Statistical significance was set at a two-tailed alpha level of 0.05. Heterogeneity across studies was quantified using the I^2^ statistic, with values above 50% indicating substantial heterogeneity. When considerable heterogeneity was detected, its potential sources were explored through sensitivity or subgroup analyses, as appropriate. Studies were assigned to each synthesis group based on their design and comparator type. Controlled studies with a placebo or vehicle comparator were included in the placebo-controlled synthesis, studies comparing two capsaicin concentrations were assigned to the dose-comparison synthesis, and uncontrolled studies were included in the pre–post synthesis.

## 3. Results

### 3.1. Included Studies

A total of 5057 records were initially identified through the systematic database search. After duplicate removal, 1939 unique records remained. Following title and abstract screening, 1882 records were excluded for not meeting the inclusion criteria. The remaining 57 articles were retrieved for full-text assessment. In addition, three relevant studies were identified through citation tracking of included articles [25,33,34]. Ultimately, nine studies met the eligibility criteria and were included in the systematic review [25,27,28,29,33,34,35,36,37]. The study selection process is summarized in the PRISMA flow diagram (Figure 1).

### 3.2. Demographic Data and Clinical Features

A total of 164 participants were included across the nine studies evaluating the effects of capsaicin on different subgroups of orofacial pain. Study durations varied considerably, including both cross-sectional designs and longitudinal studies lasting up to two years. Most studies involved small to moderate sample sizes, ranging from as few as 2 participants to a maximum of 50. The participants’ mean ages ranged from 27.4 to 65.4 years, with a predominance of female patients across studies [132 female (81.48%) and 30 male (18.52%)] (Supplementary File S5).

**Figure 2:**
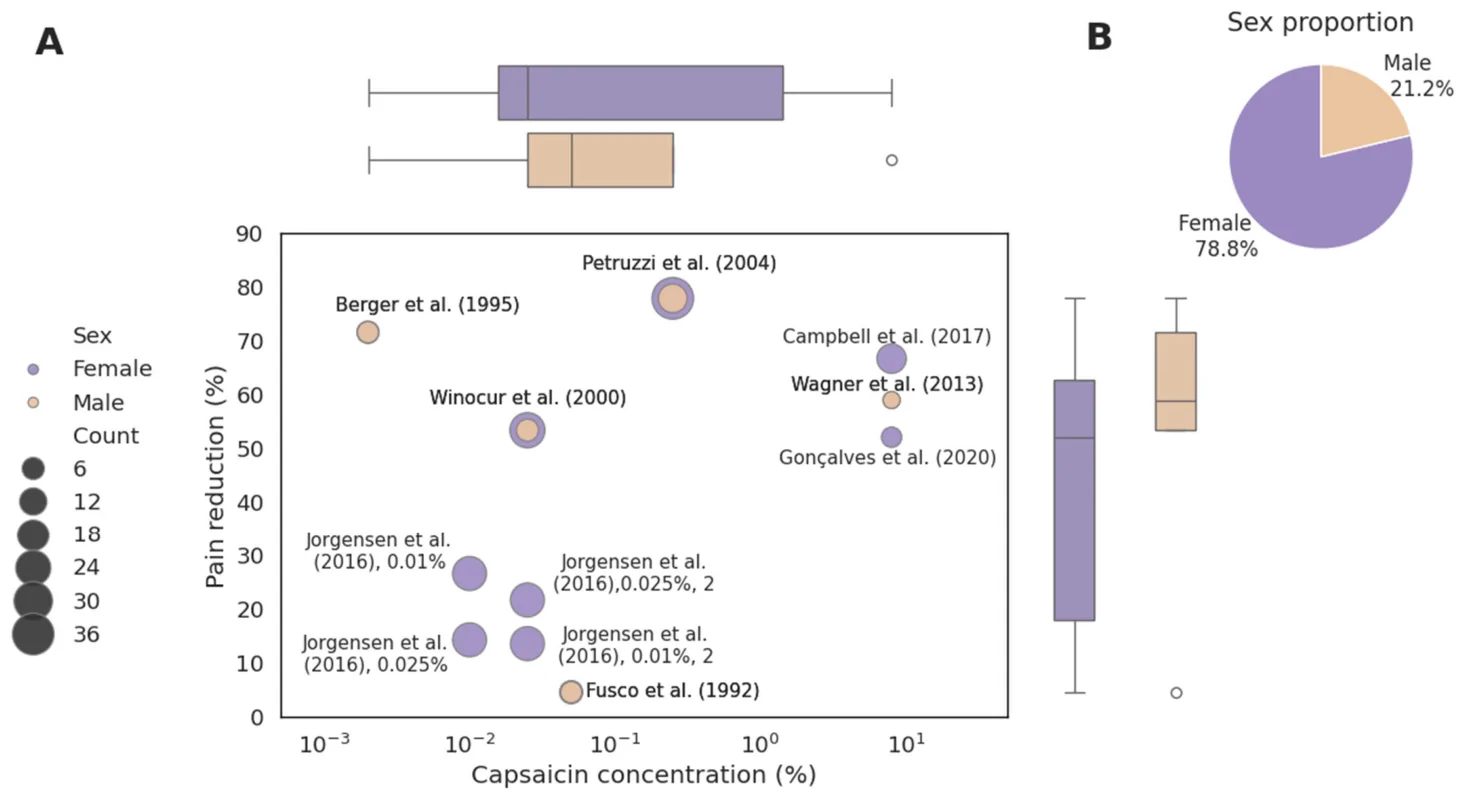
Relationship between capsaicin concentration and pain reduction according to participant sex across the included studies: Jorgensen et. al (2016) [25], Winocur et. al (2000) [27], Campbell et. al (2017) [28], Fusco et. al (1992) [29], Berger et. al (1996) [33], Wagner et. al (2013) [35], Petruzzi et. al (2004) [36], and Gonçalves et. al (2020) [37]. (**A**) Bubble scatter plot showing the association between capsaicin concentration (%) (logarithmic scale) and pain reduction (%). Bubble size is proportional to the number of participants included in each study, and colors indicate participant sex (purple = female; pink = male). The marginal boxplots summarize the distributions of capsaicin concentration (top) and pain reduction (right) according to sex. (**B**) Pie chart showing the overall sex distribution of participants across the included studies. Pain reduction data were extracted from studies reporting outcomes using the Pain Severity Scale (0–10), Visual Analogue Scale (VAS, 0–10), Numeric Rating Scale (NRS, 0–10), or Numeric Pain Rating Scale (0–11). For Jorgensen et al. (2016) [25], each treatment arm was plotted separately (0.01% and 0.025% capsaicin) to reflect the crossover design. This figure was generated using Python 3.12 (Matplotlib).

**Figure 3:**
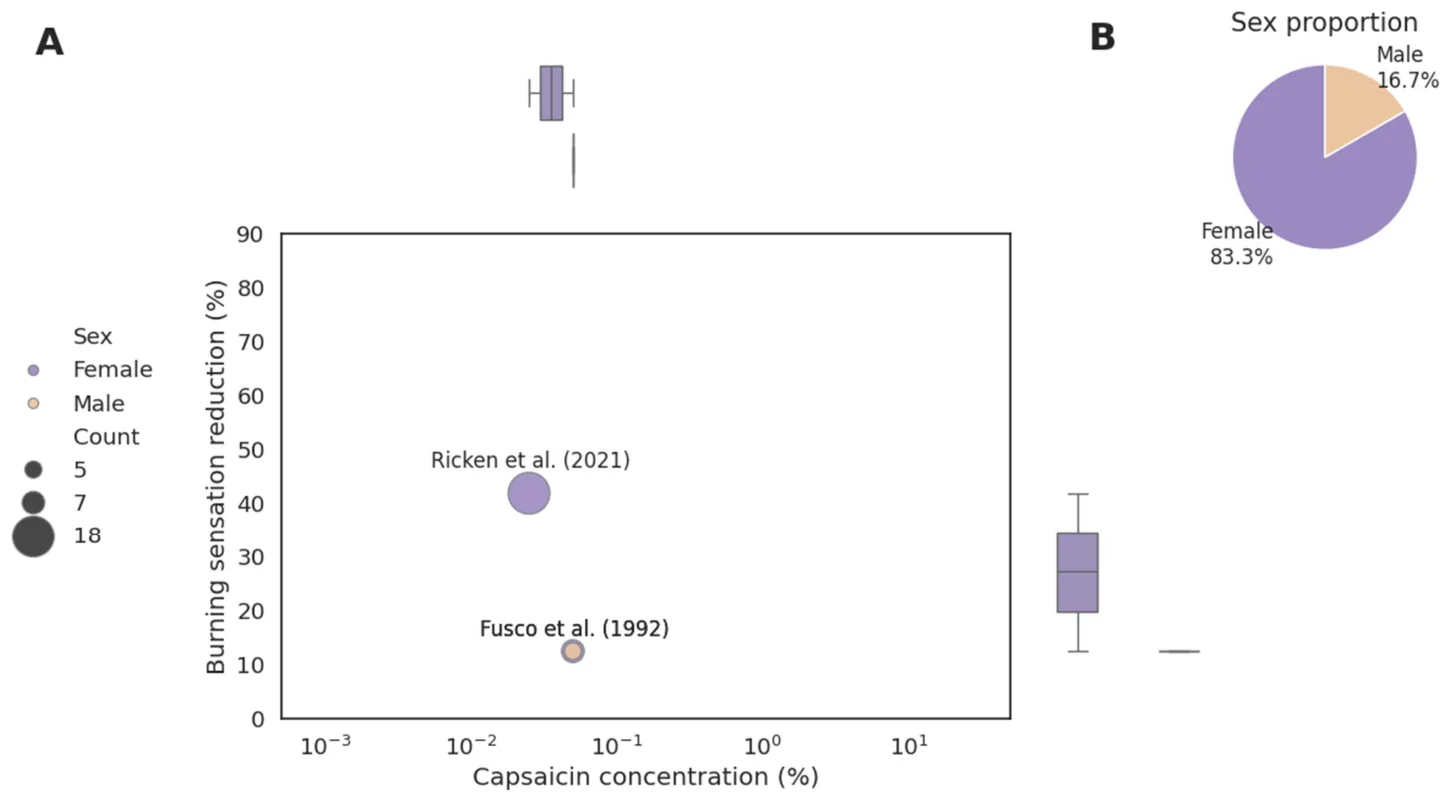
Relationship between capsaicin concentration and burning sensation reduction according to participant sex across the included studies: Fusco et. al (1992) [29] and Ricken et. al (2021) [34]. (**A**) Bubble plot showing the relationship between capsaicin concentration (%) (logarithmic scale) and burning sensation reduction (%). Bubble size is proportional to the number of participants included in each study, and bubble color indicates participant sex (purple = female; pink = male). The marginal boxplots summarize the distributions of capsaicin concentration (top) and burning sensation reduction (right) according to sex. (**B**) Pie chart showing the overall sex distribution of participants across the included studies. Data used from the authors who assessed “burning sensation 0–4 scale”. This figure was generated using Python 3.12 (Matplotlib).

Figure 2 and Figure 3 show the distribution relation among capsaicin concentration, pain reduction and sex in studies that reported pain scale ranging from 0 to ≥10 (Figure 2) and burning sensation scale 0 to 4 (Figure 3). Studies that used an 8% capsaicin concentration reported similar outcomes and substantial pain reduction [28,35,37]. Among the studies that employed lower capsaicin concentrations, Berger et al. (1995) [33], Winocur et al. (2000) [27], and Petruzzi et al. (2004) [36] demonstrated the greatest reductions in pain (Figure 2).

Regarding the reduction in burning sensation using a burning sensation scale 0 to 4, lower capsaicin concentrations were used (0.025% and 0.05%), with the 0.025% concentration showing a greater reduction in burning sensation compared with the 0.05% concentration (Figure 3) [29,34]. A marked predominance of female participants across the included studies, with women representing approximately 78.8% of the total sample for pain-reduction assessment (Figure 2) and 83.3% for burning sensation reduction (Figure 3). Pain-reduction outcomes appear broadly distributed across both sexes; however, the bubble plot suggests that most data points with higher sample sizes correspond to female-majority cohorts. The boxplots indicate comparable variability in pain-reduction percentages between sexes, though female groups display a slightly wider range of responses. Overall, the visualization highlights the sex imbalance in study populations and the heterogeneity in pain perception outcomes.

**Figure 4:**
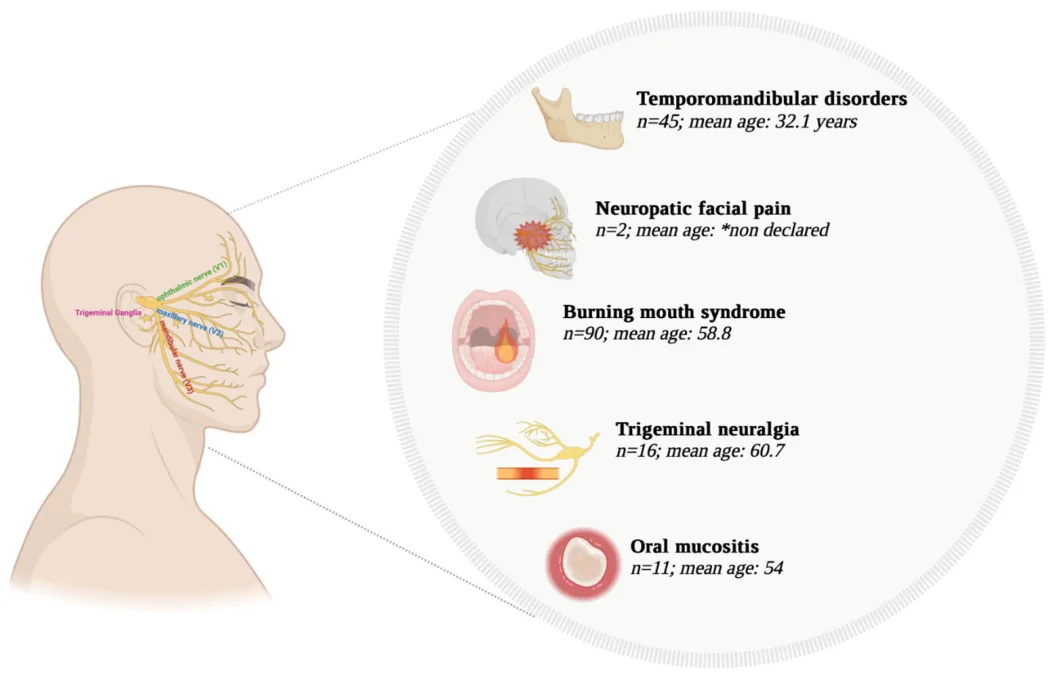
Schematic figure showing the lesions evaluated by the included studies, with the number of participants and mean age for each condition. * Indicates non declared data. Figure created using BioRender.com.

In the schematic figure, it is possible to observe the illustrated clinical conditions evaluated in the included studies, along with the corresponding number of participants and mean age for each condition (Figure 4). They comprised the temporomandibular disorders (TMDs) [27,28], burning mouth syndrome (BMS) [25,34,36], oral mucositis [33], trigeminal neuralgia [29,37], and neuropathic facial pain [35].

### 3.3. Clinical Outcomes and Symptom Improvement

Capsaicin treatment was generally associated with reductions in pain scores; however, the magnitude and consistency of this effect varied across studies, depending on the condition treated, capsaicin concentration, and study design [25,27,28,29,33,34,35,36,37]. In patients with temporomandibular disorders (TMD), topical application of 8% capsaicin resulted in a 66.7% reduction in VAS scores compared to 45.9% with placebo [28]. In contrast, treatment with 0.025% capsaicin cream did not yield a statistically significant difference between the treatment and placebo groups, despite reports of slight subjective improvement [27]. In burning mouth syndrome (BMS), systemic capsaicin improved symptoms in 76% of treated patients [36], and topical application of 0.025% gel led to complete symptom remission in 60% of cases, with additional patients experiencing partial improvement; all participants reported better quality of life scores over time [34]. Another study using both 0.01% and 0.025% topical gels reported a significant pain decrease (mean VAS reduction of 1.4 points; *p* = 0.002), with greater improvement among those with longer symptom duration [25]. In cancer patients with oral mucositis, capsaicin-containing lozenges produced a 71.6% reduction in pain severity after initial use, with all patients reporting relief [33]. For trigeminal neuralgia, 0.05% topical capsaicin cream resulted in complete pain remission in half of the patients, partial relief in others, and successful retreatment in relapsing cases [29]. The 8% capsaicin patch also demonstrated delayed but sustained pain reduction over 12 weeks in 50% of patients, following an initial pain flare [37].

### 3.4. Critical Appraisal of the Included Studies

**Figure 5:**
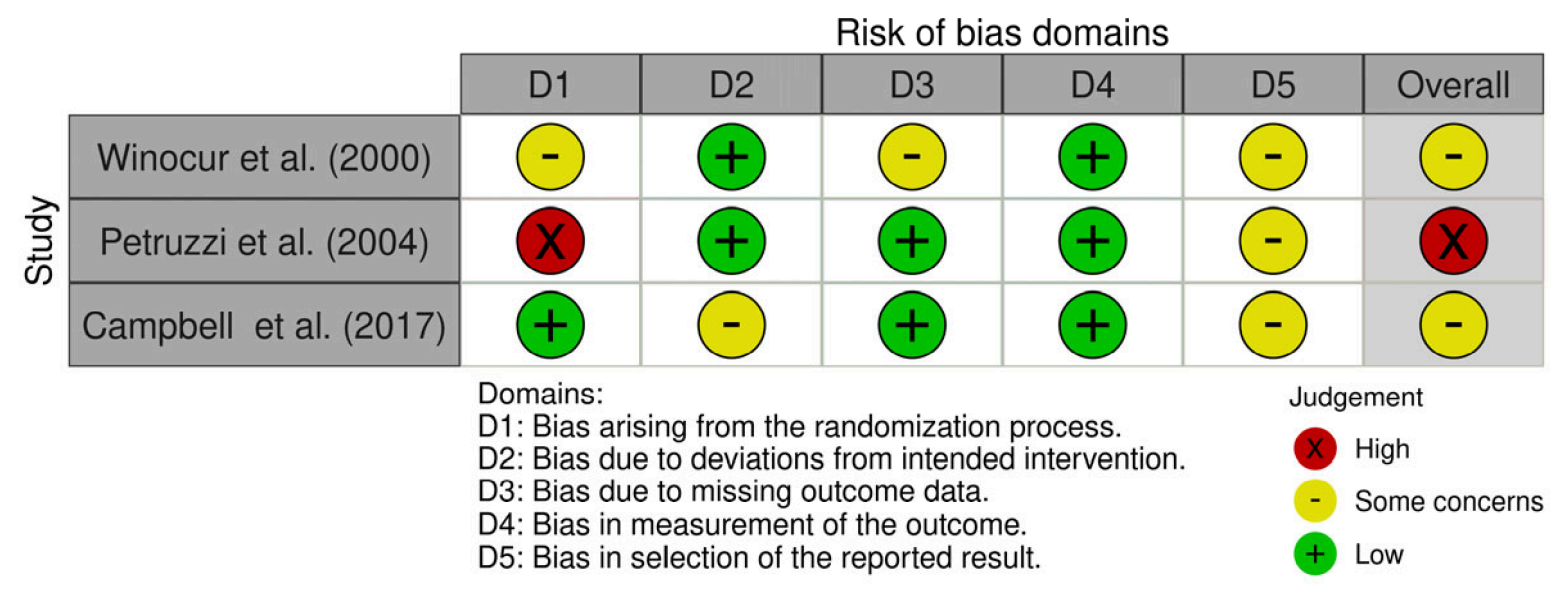
Traffic light plot of the risk of bias assessment for the included randomized controlled trials using the Cochrane Risk of Bias 2 (RoB 2) tool. Each circle represents the judgment for an individual domain and the overall risk of bias for each study (green = low risk, yellow = some concerns, red = high risk). The figure was generated using the robvis visualization tool. The studies [27,28,36] were assessed.

The risk of bias assessment of the three randomized controlled trials using the Cochrane RoB 2 tool is presented in Figure 5 [27,28,36]. Overall, one study was judged as having a high risk of bias, whereas the remaining two studies were judged as having some concerns.

Bias arising from the randomization process showed the greatest variability across studies, with one study rated as low risk, one as having some concerns, and one as high risk. The high-risk judgment was primarily related to inadequate randomization procedures and lack of allocation concealment.

Bias due to deviations from intended interventions and bias due to missing outcome data were generally low, although one study raised some concerns in each of these domains because participants who discontinued treatment were excluded from the final analysis rather than being analyzed according to the intention-to-treat principle.

All studies were judged to have a low risk of bias in the measurement of the outcome, as pain was assessed using validated instruments and comparable assessment procedures were applied across intervention groups.

The domain “selection of the reported result” was judged as having some concerns for all studies because no publicly available pre-specified statistical analysis plan could be identified, preventing confirmation that the reported analyses had been finalized before unblinded outcome data became available.

**Figure 6:**
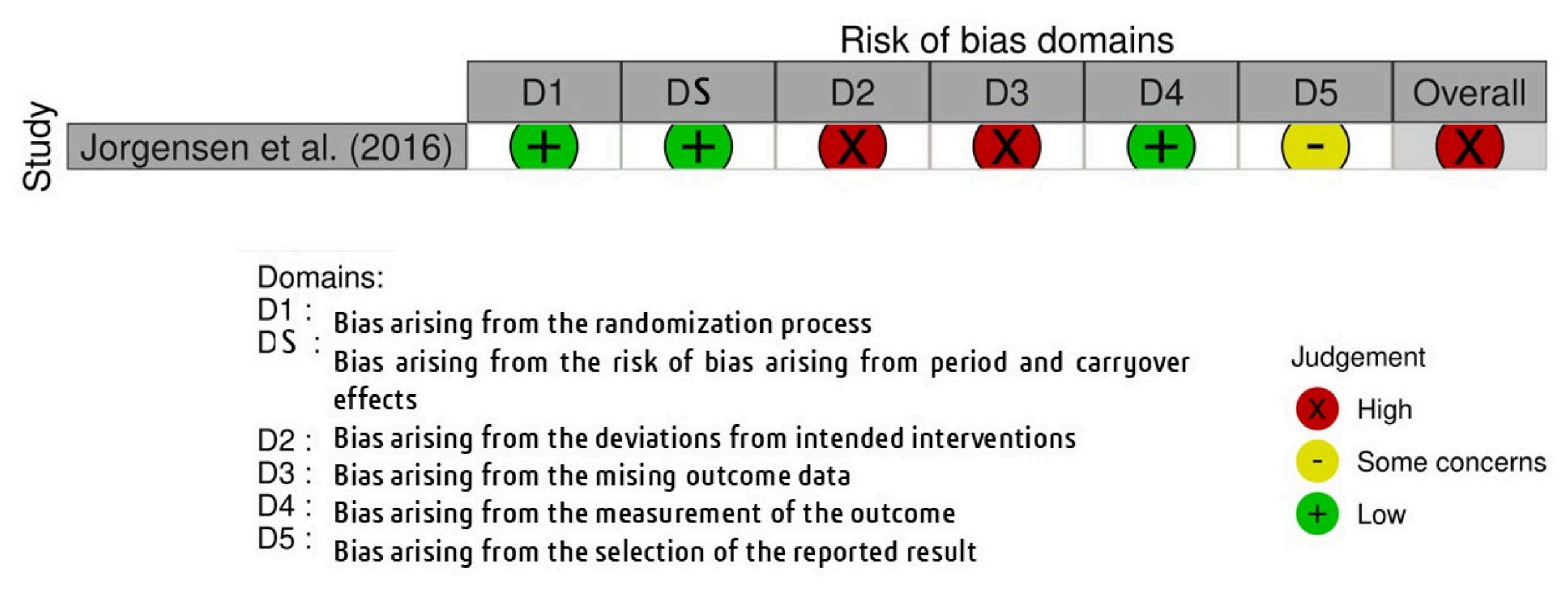
Risk of bias assessment of the included crossover randomized controlled trial Jorgensen et al. 2016 [25] using the Cochrane Risk of Bias 2 tool for crossover trials (RoB 2). Traffic light plot showing the judgment for each risk-of-bias domain, including the additional crossover domain, and the overall risk of bias (green = low risk, yellow = some concerns, red = high risk). The figure was generated using the robvis visualization tool. The study [25] was assessed.

The crossover trial was assessed using the RoB 2 tool adapted for crossover studies (Figure 6) [25]. The study was judged as low risk of bias for the randomization process, additional crossover considerations, and outcome measurement. However, it was judged as high risk of bias for deviations from intended interventions and missing outcome data because four randomized participants discontinued treatment due to intervention-related adverse effects and were excluded from the final analysis. Some concerns were identified regarding selection of the reported result owing to the absence of a publicly available pre-specified statistical analysis plan. Consequently, the overall risk of bias for the crossover trial was judged as high.

**Figure 7:**
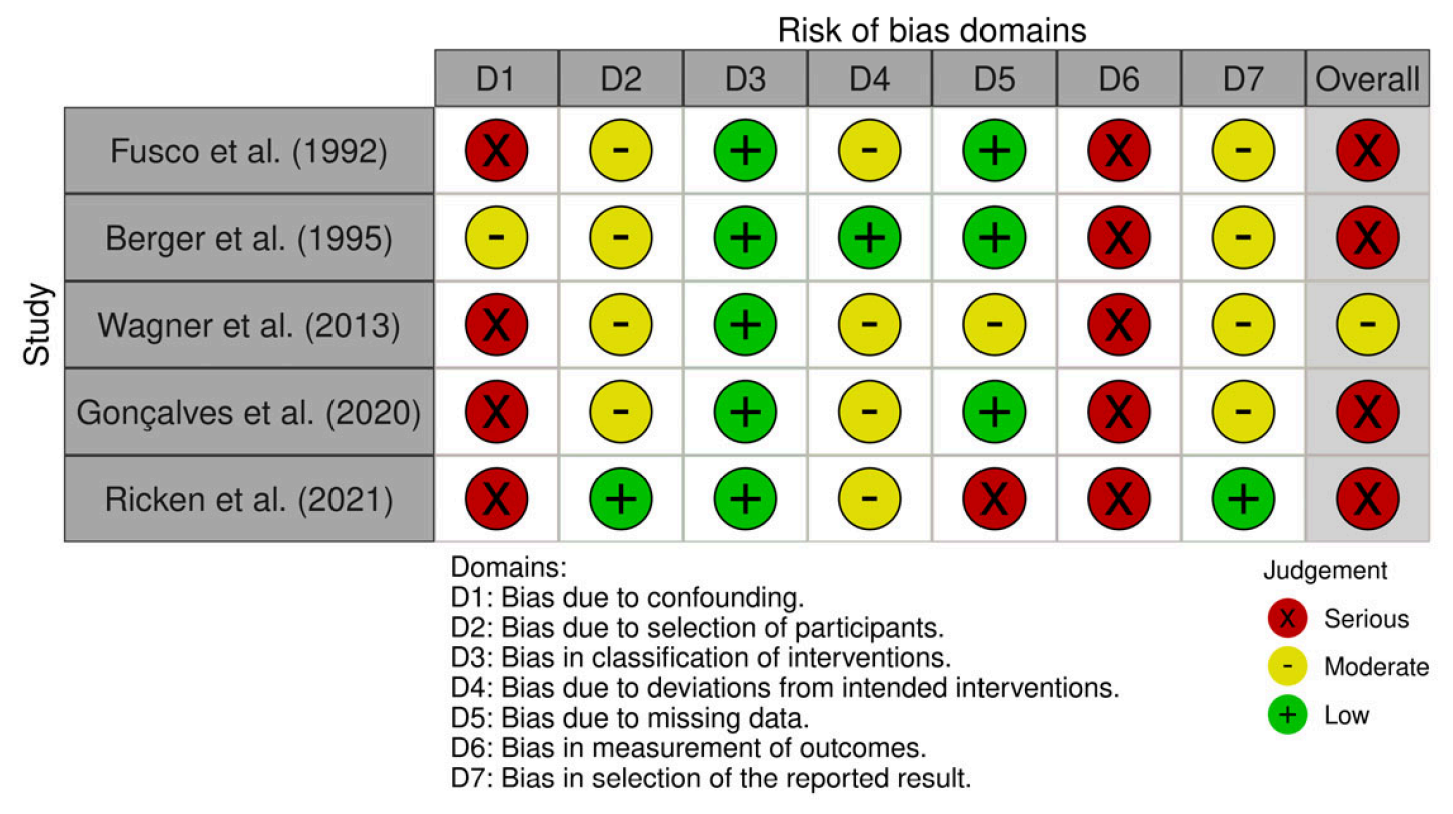
Risk of bias assessment of the non-randomized intervention studies using the ROBINS-I tool. Traffic light plot summarizing the risk of bias judgments for each included study across the seven ROBINS-I domains, and the overall risk of bias (green = low risk, yellow = some concerns, red = high risk). The figure was generated using the robvis visualization tool. The articles [29,33,34,35,37] were assessed.

Overall, the non-randomized studies presented predominantly serious to moderate risks of bias [29,33,34,35,37], with four of the five studies being judged as having an overall serious risk of bias, while one study [35] was judged as having moderate risk of bias (Figure 7). The most frequent sources of bias were confounding (Domain 1) and measurement of outcomes (Domain 6), reflecting the absence of randomization, lack of control for important prognostic variables, and the use of subjective pain outcomes without blinding. Moderate concerns were commonly identified for selection of participants (Domain 2), deviations from intended interventions (Domain 4), and selection of the reported result (Domain 7). Conversely, most studies demonstrated a low risk of bias in the classification of interventions (Domain 3), as treatment status was clearly defined, and missing data (Domain 5) was generally judged as low risk, except for Wagner et al. (2013) [35] and Ricken et al. (2021) [34], where attrition raised additional concerns.

Detailed risk of bias assessments for the clinical trials (RoB 2), crossover clinical trial (RoB 2 for crossover trials), and non-randomized intervention studies (ROBINS-I) are provided in the Supplementary Materials.

### 3.5. Certainty of the Evidence

The certainty of the evidence was assessed using the GRADE approach (Supplementary File S6). For randomized controlled trials comparing capsaicin with placebo or lower-dose capsaicin [25,27,28,36], the certainty of the evidence for orofacial pain reduction was rated as very low, primarily because of serious concerns regarding risk of bias, substantial heterogeneity among studies, and imprecision of the pooled estimate. In contrast, the certainty of the evidence for capsaicin-related adverse events was rated as moderate, as the evidence was downgraded only for risk of bias, while no serious concerns were identified regarding inconsistency, indirectness, or imprecision [27,28,36].

For non-randomized intervention studies [29,34,37], the certainty of the evidence for pain reduction was rated as low because of serious risk of bias and imprecision, despite consistent findings across studies. The complete Summary of Findings table, including the rationale for each GRADE judgment, is provided in the Supplementary Materials.

### 3.6. Meta-Analysis

**Figure 8:**
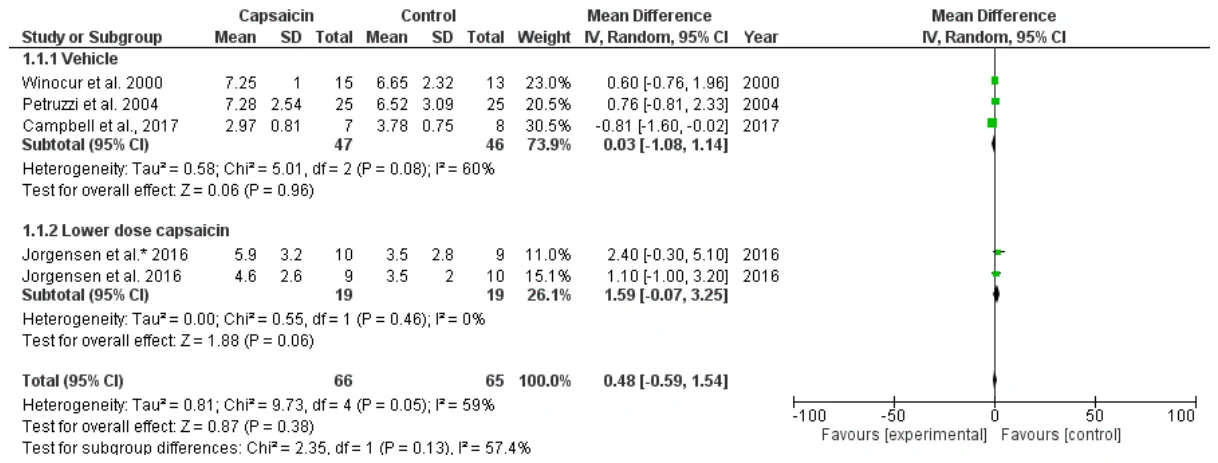
Effect of capsaicin on orofacial pain reduction, expressed as mean pain scale scores and stratified according to the comparator in the baseline. The ‘low dose capsaicin’ subgroup includes comparisons between 0.025% and 0.01% capsaicin from the crossover study, with each comparison arm presented separately in the revised analysis. Green squares represent individual study estimates (with square size proportional to study weight), and black diamonds represent the pooled effect estimates with their corresponding 95% confidence intervals. * Indicates a distinct treatment arm from the same crossover study [25]. The controlled studies included Winocur et al. [27], Campbell et al. [28] and Petruzzi et al. [36].

To evaluate the effect of capsaicin on orofacial pain, a meta-analysis was performed using the included randomized controlled trials [25,27,28,36]. Baseline pain intensity was first analyzed to assess the comparability of the intervention groups before treatment. No significant differences were observed between the capsaicin and control groups (placebo or lower-dose capsaicin), indicating comparable baseline pain levels (MD = 0.48, [95% CI: −0.59, 1.54]; I^2^ = 59%; n= 66; p = 0.38) (Figure 8). At the end of treatment, capsaicin showed a trend toward greater reduction in orofacial pain compared with the vehicle control groups. However, the pooled effect did not reach statistical significance (MD = −1.87, [95% CI −3.94, 0.19]; I^2^ = 86%; n = 47; p = 0.08) [27,28,36].

Since Jorgensen et al. (2016) [25] was a crossover trial, the data from each treatment period were included separately in the meta-analysis as individual comparisons, thereby preserving the treatment-specific effects reported by the authors. No significant difference in pain reduction was observed between the two capsaicin concentrations (0.025% vs. 0.01%) (MD = 0.46, [95% CI: −2.67, 3.60]; I^2^ = 66%; n = 19; p = 0.7738).

**Figure 9:**
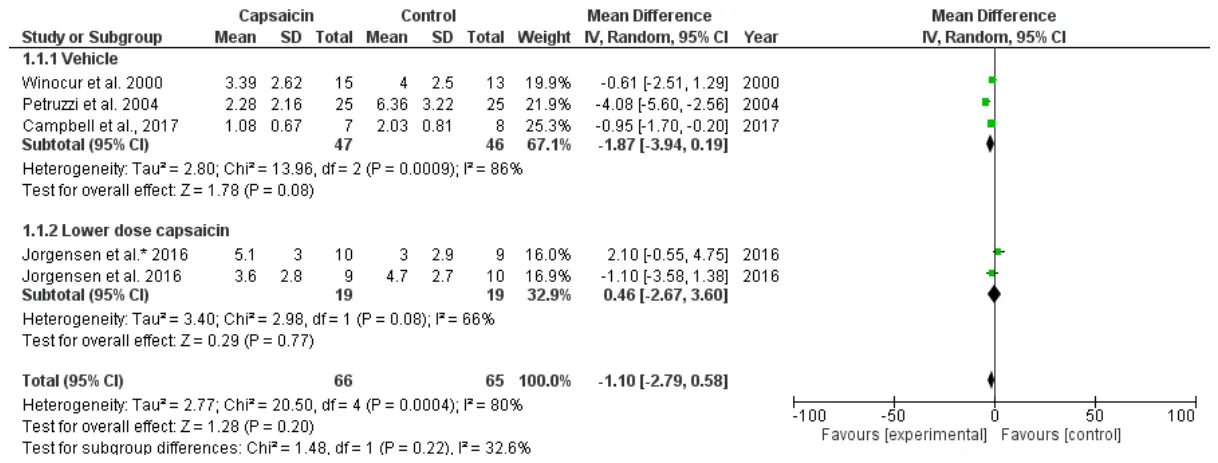
Effect of capsaicin on orofacial pain reduction, expressed as mean pain scale scores and stratified according to the comparator in the final intervention. The ‘low dose capsaicin’ subgroup includes comparisons between 0.025% and 0.01% capsaicin from the crossover study, with each comparison arm presented separately in the revised analysis. Green squares represent individual study estimates (with square size proportional to study weight), and black diamonds represent the pooled effect estimates with their corresponding 95% confidence intervals. * Indicates a distinct treatment arm from the same crossover study Jorgensen et al. [25]. The controlled studies included Winocur et al. [27], Petruzzi et al. [36], and Campbell et al. [28].

When all eligible randomized studies, including the placebo-controlled trials and the separate comparisons from Jorgensen et al. (2016) [25] were pooled, capsaicin showed a trend toward greater reduction in orofacial pain compared with the control interventions. However, the pooled effect did not reach statistical significance, and substantial heterogeneity was observed across studies (MD = −1.10, [95% CI −2.79,0.58]; I^2^ = 80%; n = 66; p = 0.20) (Figure 9) [25,27,28,36].

**Figure 10:**
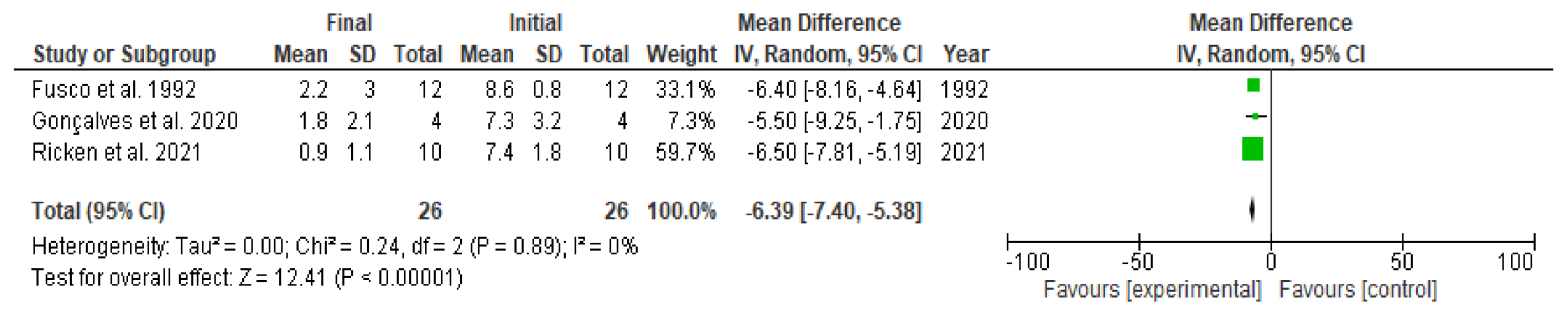
Comparison of the effect of capsaicin on orofacial pain reduction between baseline and the final assessment, based on the mean scores of orofacial pain scales reported in uncontrolled (single-arm) studies Fusco et al. [29], Ricken et al. [34], Gonçalves et al. [37], regardless of follow-up duration. Green squares represent individual study estimates (with square size proportional to study weight), and black diamonds represent the pooled effect estimates with their corresponding 95% confidence intervals.

For uncontrolled (single-arm) studies, we performed a meta-analysis comparing pain levels before and after capsaicin treatment. A significant reduction in pain was observed following capsaicin use (MD = −6.39 [95% CI: −7.40, −5.38]; I^2^ = 0%; n = 26; p < 0.001) (Figure 10) [29,34,37].

**Figure 11:**
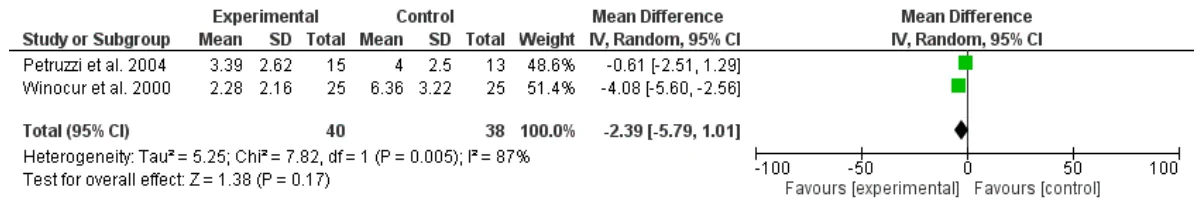
Comparison of the effect of capsaicin on reducing orofacial pain compared to vehicle during 30 days of use, based on mean values obtained for orofacial pain scale scores in the selected studies. Green squares represent individual study estimates (with square size proportional to study weight), and black diamonds represent the pooled effect estimates with their corresponding 95% confidence intervals. The controlled studies included and Winocur et al. [27] and Petruzzi et al [36].

**Figure 12:**
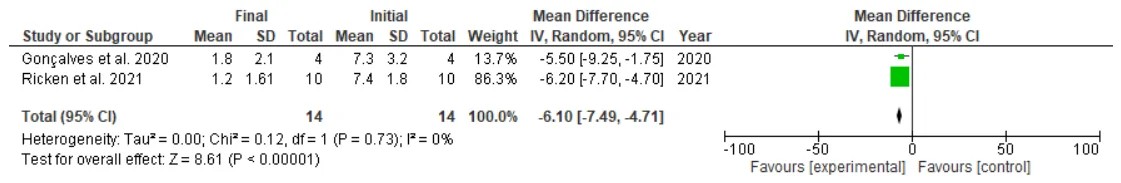
Meta-analysis of within-group changes in orofacial pain from baseline to the same post-treatment time point after capsaicin use in uncontrolled (single-arm) studies Ricken et al. [34] and Gonçalves et al. 2020 [37]. Effect estimates were calculated using the mean scores of orofacial pain scales reported before treatment and after comparable intervention durations across studies. Green squares represent individual study estimates (with square size proportional to study weight), and black diamonds represent the pooled effect estimates with their corresponding 95% confidence intervals.

Additionally, in the longer-term follow-up analyses (30 days for controlled studies and 12 weeks for uncontrolled (single-arm) studies), the pooled analysis of controlled studies [27,36] showed a greater reduction in orofacial pain with capsaicin than with control interventions; however, the difference was not statistically significant (MD = −2.39, [95% CI −5.79, 1.01]; I^2^ = 87%; n = 40; p = 0.17). In contrast, the pooled within-group analysis of uncontrolled (single-arm) studies [34,37] evaluating comparable intervention durations demonstrated a significant reduction in orofacial pain over time following capsaicin treatment (MD = −6.10, [95% CI −7.49, −4.71]; I^2^ = 0%; p < 0.0001) (Figure 11 and Figure 12) [27,34,36,37].

**Figure 13:**
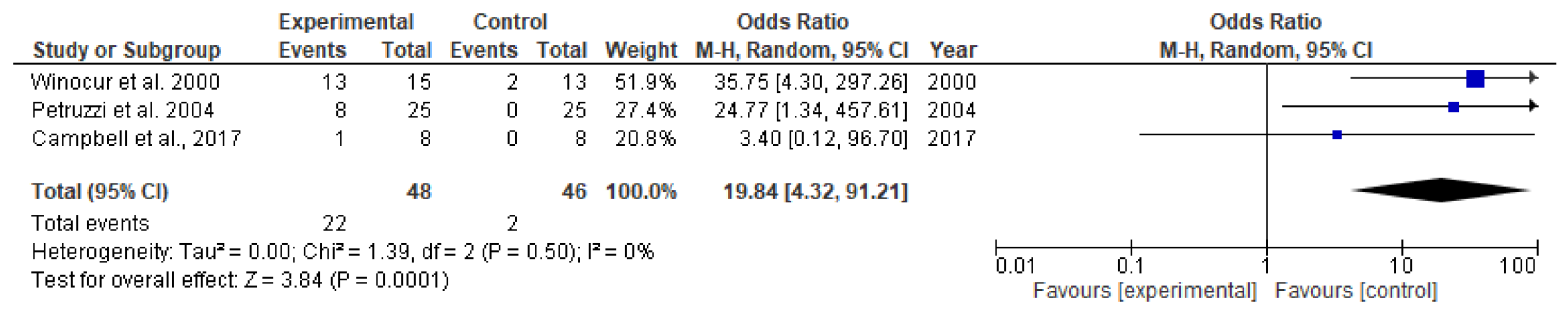
Comparison of the incidence of treatment-related adverse events associated with capsaicin versus control, based on absolute event frequencies reported across the included studies. The pooled outcome included any reported adverse event, such as warm sensation, stinging, burning, redness, gastric pain, and burning/pain at the application site. Regarding adverse effects, capsaicin was associated with a significantly higher likelihood of side effects compared to control (vehicle), with an odds ratio of 19.84 (95% CI: 4.32, 91.21; I^2^ = 0%; n = 48; *p* < 0.001). Blue squares represent individual study estimates (with square size proportional to study weight), and black diamonds represent the pooled effect estimates with their corresponding 95% confidence intervals. The controlled studies included Winocur et al. [27], Campbell et al. [28] and Petruzzi et al. [36].

Regarding adverse effects, capsaicin was associated with a significantly higher likelihood of side effects compared to control (vehicle), with an odds ratio of 19.84 (95% CI: 4.32, 91.21; I^2^ = 0%; n = 48; p < 0.001) [27,28,36] (Figure 13).

## 4. Discussion

Orofacial pain is a prevalent and impactful clinical condition that significantly compromises patients’ quality of life, often contributing to emotional distress and imposing a considerable socioeconomic burden [5,6,7]. Its origin is frequently linked to the activation of the trigeminal nerve, which provides sensory innervation to the face and oral cavity through nociceptors located in Aδ and C fibers, with cell bodies in the trigeminal ganglion [38]. Nociceptive transmission involves the activation of brainstem nuclei and higher central structures, ultimately resulting in pain perception [2,6].

Managing orofacial pain remains a clinical challenge, as many available therapies offer limited efficacy and are often associated with adverse effects [39,40,41]. In this context, the findings of this review highlight capsaicin as a promising therapeutic alternative. Across the included studies, capsaicin demonstrated a consistent trend toward reducing orofacial pain in various conditions, including TMD [27,28], burning mouth syndrome [25,34,36], oral mucositis [33], trigeminal neuralgia [29,37], and neuropathic facial pain [35]. Although the meta-analysis of placebo-controlled studies did not reach statistical significance (*p* = 0.08), showing high heterogeneity (I^2^ = 86%) [27,28,36], no statistically significant difference was found between capsaicin concentrations in the dose-comparison analysis (*p* = 0.77). The meta-analysis of uncontrolled (single-arm) studies showed a significant reduction in pain from baseline to the final assessment when all eligible studies were included [29,34,37]. A separate analysis restricted to studies with comparable longer-term follow-up periods also demonstrated a significant reduction in pain [34,37].

An important limitation of this meta-analysis is that studies addressing different orofacial pain conditions, including temporomandibular disorders, burning mouth syndrome, oral mucositis, and trigeminal/neuropathic facial pain were pooled within the same quantitative synthesis rather than analyzed in condition-specific subgroups. These conditions differ substantially in underlying pathophysiology, encompassing musculoskeletal, mucosal, and neuropathic pain mechanisms, as well as in baseline severity and expected treatment response. This clinical heterogeneity likely contributed to the substantial statistical heterogeneity observed in the controlled comparisons (I^2^ = 80–87%) and may partly explain the discordance between the non-significant placebo-controlled estimate and the significant effect observed in uncontrolled analyses. Consequently, the pooled estimates should be interpreted cautiously, as the overall effect may be influenced by clinical and methodological variability across studies. While the findings support the potential therapeutic role of capsaicin, the current evidence remains insufficient to draw definitive conclusions regarding its efficacy across all orofacial pain conditions.

Capsaicin is a highly selective agonist of transient receptor potential vanilloid 1 (TRPV1) channel, located in C fibers and some Aδ fibers of the skin [40]. It is one of the few treatments approved by the Food and Drug Administration (FDA) for postherpetic neuralgia (PHN), either alone or in association with diabetic peripheral neuropathy. In Europe, it is approved for the treatment of peripheral neuropathic pain in adults, either as monotherapy or in combination with other analgesic agents [19]. Beyond its analgesic properties, capsaicin has attracted considerable interest for its ability to inhibit oral cancer cell lines [41,42,43,44], oral epithelial dysplasia [44], and the growth of oral pathogens associated with dental caries, periodontitis and oral candidiasis [45,46].

The antinociceptive mechanism of capsaicin is primarily related to the sustained activation of TRPV1, a non-selective cation channel predominantly expressed in primary afferent C fibers [16,40,42]. Prolonged TRPV1 activation induces calcium and sodium influx, leading to neuronal depolarization and an initial burning sensation. However, this overstimulation results in functional desensitization of nociceptive fibers through mechanisms such as depletion of neuropeptides (e.g., substance P, NKA, CGRP), disruption of axonal transport, mitochondrial damage, and collapse of peripheral nerve terminals [43,47]. These effects culminate in a temporary loss of sensory function in the affected fibers, resulting in decreased pain perception [48]. Notably, preclinical studies have also demonstrated that excessive or prolonged exposure to capsaicin may induce neuronal apoptosis through calcium-dependent activation of the caspase cascade in rats [41,47]. In humans, topical capsaicin leads to temporary degeneration and impairment of sensory and autonomic nerve fiber function in health individuals. Baseline function is typically restored after 40–50 days in autonomic fibers and 140–150 days in sensory fibers [48]. These findings suggest capsaicin’s effects may vary according to exposure conditions, developmental stage, and neuronal vulnerability.

The studies included in this review employed a variety of capsaicin administration and concentrations, including low-concentration topical creams, and gel (0.025–0.1%), high-concentration patches (8%), and oral-systemic administration (0.25%, and 0.005–0.008 g). Notably, studies employing higher capsaicin concentrations generally reported greater pain reduction; however, these studies also tended to include smaller sample sizes [28,35]. In contrast, some studies using lower concentrations demonstrated pronounced analgesic effects, which may be partly explained by larger sample sizes and by the specificity of the pain condition evaluated [25,26,27,35]. This finding suggests that the analgesic effect of capsaicin does not follow a simple linear dose–response relationship and that low concentrations may be sufficient to clinically lead TRPV1 desensitization [16]. In addition, an interesting aspect of capsaicin is that its high-concentration use may lead to lengthened time for it to act on the TRPV1 receptors, especially in slower capsaicin metabolism organs, such as the central nervous system and the skin, increasing the likelihood of adverse events [49]. This prolonged receptor engagement may partly explain the higher incidence of local adverse events reported with high-concentration formulations (e.g., 8% capsaicin patches) compared with lower-concentration, more frequently applied preparations, as sustained TRPV1 activation delays the onset of the desensitization process that ultimately underlies capsaicin’s analgesic effect. Consequently, the initial period of treatment before desensitization is achieved and represents the window of greatest vulnerability to adverse events, particularly in anatomically sensitive regions such as the orofacial area, where the skin and mucosa are richly innervated and in close proximity to the eyes and airway.

Beyond its peripheral actions, capsaicin also exerts central effects by modulating neurotransmitter release in the central nervous system [49]. Prolonged stimulation of peripheral nociceptors induces the release of glutamate and neuropeptides in the trigeminothalamic tract and substantia gelatinosa of the brainstem, thereby influencing second-order neurons involved in pain processing [47,49]. Therefore, topical or systemic administration of capsaicin represents a promising strategy for modulating orofacial pain. It is worth mentioning that the combined central and peripheral use of capsaicin may lead to persistent allodynia and hyperalgesia even when inflammation resolves post-capsaicin application as observed in chronic neuropathic pain, with not enough evidence to determine whether central hypersensitivity resolves as seen peripherally with capsaicin [48,49].

Another important finding from the included studies was the predominance of female participants with orofacial pain [50,51]. Epidemiological studies have shown a higher prevalence of such pain in adult women, especially during the reproductive years. [51]. This pattern may be related to genetic factors as well as hormonal and psychosocial factors [46,52,53,54]. A systematic review reported that ten studies confirmed the influence of estrogen on the increase in pain associated with TMD [46]. The mechanism involved suggests that estradiol (especially 17β-estradiol) can enhance inflammation in the TMJ via estrogen receptors (ESR1), activating matrix metalloproteinases MMP-9 and MMP-13 [45]. Additionally, the local production of estradiol by aromatase-positive neurons in the trigeminal system may influence neuronal excitability and pain perception, contributing to the higher susceptibility of women to TMD and persistent orofacial pain [53]. With data derived from a twin study, Visscher et al. (2018) [52] investigated the genetic relationship associated with TMD pain, showing that approximately 35% of the variance in TMD pain can be attributed to genetic factors. Additionally, the study revealed a significant genetic correlation (r = 0.64) between TMD pain and neck pain, indicating that more than half of the comorbidity between these two conditions is explained by shared genetic factors. 

Furthermore, the mean age of participants in the included studies was frequently in the older adult range. Normative age-related declines in endogenous pain modulatory systems are believed to contribute to increased pain intensity with aging. Older adults have been shown to rate heat pain as more intense and unpleasant compared to younger adults and demonstrate impaired diffuse noxious inhibitory control, suggesting diminished endogenous inhibitory capacity [55]. Similarly, Derafshi et al. [56] investigated the prevalence of chronic orofacial pain in elderly patients and found that 11.16% reported orofacial pain as their main complaint. The average age was 55.3 ± 10.7 years, ranging from 46 to 90. The study identified advanced age as a significant risk factor for chronic orofacial pain, particularly in cases of neuropathies and burning mouth syndrome (BMS). Emotional disorders such as depression and anxiety were also commonly associated with these conditions [54,56].

Although our systematic review showed that capsaicin is effective in improving orofacial pain, caution is warranted due to methodological variability, the generally small sample sizes across studies, and the risk of bias identified in the randomization process of one of the included trials. Furthermore, although capsaicin was associated with a significantly higher incidence of adverse effects compared to control (OR = 19.84), these were mostly transient and localized, such as burning sensations or erythema.

This work presents some limitations. First, many included studies were uncontrolled, which may introduce bias due to the lack of comparison groups. Second, among the controlled studies, most were placebo-controlled, which may have led to blinding bias. This is particularly relevant for the capsaicin group, as the burning sensation it produces could have revealed to participants that they were receiving active treatment. Third, the discrepancy in sample sizes across studies may have contributed to the high heterogeneity observed in the meta-analyses. Fourth, the lack of information on individual risk factors for orofacial pain, such as age, sex, psychological distress, parafunctional habits (e.g., bruxism), hormonal status, and comorbidities such as anxiety, depression, and sleep disorders, in some studies represents an important limitation, as these variables can influence treatment outcomes. Fifth, the method of pain assessment was not standardized across the studies. Sixth, publication bias was not formally evaluated because each meta-analysis included fewer than 10 studies. According to current methodological guidance, funnel plots and tests for funnel plot asymmetry are unreliable in meta-analyses with a limited number of studies, as they have insufficient power to distinguish true asymmetry from chance. Finally, a systematic review and meta-analysis comparing capsaicin with standard pharmacological treatments was not possible, since no eligible studies made such comparisons.

This review highlights capsaicin’s potential as a non-opioid therapeutic option capable of modulating nociceptive signaling through TRPV1 receptor desensitization. However, large-scale, multicenter randomized controlled trials would be beneficial to establish long-term efficacy, safety, optimal dosing regimens, and comparison among capsaicin and existing market drugs for different orofacial pain conditions. Standardization of treatment protocols, outcome measures, and follow-up periods would facilitate comparisons across studies and improve the quality of evidence.

## 5. Conclusions

Capsaicin provides clinically relevant analgesia in selected orofacial pain conditions, particularly burning mouth syndrome and temporomandibular disorders. Its effect is mediated by TRPV1 desensitization, leading to reduced peripheral nociceptive signaling. Despite heterogeneity in formulations and dosing, the overall evidence consistently supports a direction of effect toward pain reduction.

Capsaicin is well tolerated in the orofacial setting and adverse effects are typically mild, transient, and localized (burning or irritation), diminishing with repeated use. Serious events are not consistently reported, and treatment discontinuation is uncommon, supporting its use as a non-opioid option in multimodal pain management.

## Data Availability

No new data were created or analyzed in this study. Data sharing is not applicable to this review article.

## Supplementary Materials

The following supporting information can be downloaded at: https://www.mdpi.com/article/10.3390/ijms27167099/s1.

## References

[1] Raja S.N., Carr D.B., Cohen M., Finnerup N.B., Flor H., Gibson S., Keefe F.J., Mogil J.S., Ringkamp M., Sluka K.A. (2020). The Revised IASP definition of pain: Concepts, challenges, and compromises. Pain.

[2] Rotpenpian N., Yakkaphan P. (2021). Review of Literatures: Physiology of Orofacial Pain in Dentistry. eNeuro.

[3] Labanca M., Gianò M., Franco C., Rezzani R. (2023). Orofacial Pain and Dentistry Management: Guidelines for a More Comprehensive Evidence-Based Approach. Diagnostics.

[4] Schiffman E., Ohrbach R., Truelove E., Look J., Anderson G., Goulet J.-P., List T., Svensson P., Gonzalez Y., Lobbezoo F. (2014). Diagnostic Criteria for Temporomandibular Disorders (DC/TMD) for Clinical and Research Applications: Recommendations of the International RDC/TMD Consortium Network* and Orofacial Pain Special Interest Group†. J. Oral Facial Pain Headache.

[5] Murray H., Locker D., Mock D., Tenenbaum H.C. (1996). Pain and the quality of life in patients referred to a craniofacial pain unit. J. Orofac. Pain.

[6] Baron R., Binder A., Wasner G. (2010). Neuropathic pain: Diagnosis, pathophysiological mechanisms, and treatment. Lancet Neurol..

[7] Porporatti A.L., Schroder Â.G.D., Lebel A., Moreau N., Guillouet C., Stechman-Neto J., Boucher Y. (2024). Prevalence of orofacial and head pain: An umbrella review of systematic reviews. J. Oral Facial Pain Headache.

[8] Julius D. (2013). TRP channels and pain. Annu. Rev. Cell Dev. Biol..

[9] Venkatachalam K., Montell C. (2007). TRP channels. Annu. Rev. Biochem..

[10] Gees M., Owsianik G., Voets T. (2012). TRP Channels. Compr. Physiol..

[11] Jara-Oseguera A., Islas L.D. (2013). The role of allosteric coupling on thermal activation of thermo-TRP channels. Biophys. J..

[12] Carnevale V., Rohacs T. (2016). TRPV1: A Target for Rational Drug Design. Pharmaceuticals.

[13] Premkumar L.S., Sikand P. (2008). TRPV1: A Target for Next Generation Analgesics. Curr. Neuropharmacol..

[14] Caterina M.J. (2007). Transient receptor potential ion channels as participants in thermosensation and thermoregulation. Am. J. Physiol. Regul. Integr. Comp. Physiol..

[15] Caterina M.J., Julius D. (1999). Sense and specificity: A molecular identity for nociceptors. Curr. Opin. Neurobiol..

[16] Caterina M.J., Schumacher M.A., Tominaga M., Rosen T.A., Levine J.D., Julius D. (1997). The capsaicin receptor: A heat-activated ion channel in the pain pathway. Nature.

[17] Wood J.N., Winter J., James I.F., Rang H.P., Yeats J., Bevan S. (1988). Capsaicin-induced ion fluxes in dorsal root ganglion cells in culture. J. Neurosci..

[18] Zheng J. (2013). Molecular mechanism of TRP channels. Compr. Physiol..

[19] Bernstein J.E., Korman N.J., Bickers D.R., Dahl M.V., Millikan L.E. (1989). Topical capsaicin treatment of chronic postherpetic neuralgia. J. Am. Acad. Dermatol..

[20] Guedon J.M.G., Yee M.B., Zhang M., Harvey S.A.K., Goins W.F., Kinchington P.R. (2015). Neuronal changes induced by Varicella Zoster Virus in a rat model of Postherpetic Neuralgia. Virology.

[21] Kulkantrakorn K., Lorsuwansiri C., Meesawatsom P. (2013). 0.025% Capsaicin Gel for the Treatment of Painful Diabetic Neuropathy: A Randomized, Double-Blind, Crossover, Placebo-Controlled Trial. Pain Pract..

[22] Tshering G., Posadzki P., Kongkaew C. (2024). Efficacy and safety of topical capsaicin in the treatment of osteoarthritis pain: A systematic review and meta-analysis. Phyther. Res..

[23] Souto E.B., Cano A., Martins-Gomes C., Coutinho T.E., Zielińska A., Silva A.M. (2022). Microemulsions and Nanoemulsions in Skin Drug Delivery. Bioengineering.

[24] Wang X.R., Gao S.Q., Niu X.Q., Li L.J., Ying X.Y., Hu Z.J., Gao J.-Q. (2017). Capsaicin-loaded nanolipoidal carriers for topical application: Design, characterization, and in vitro/in vivo evaluation. Int. J. Nanomed..

[25] Jørgensen M.R., Pedersen A.M.L. (2017). Analgesic effect of topical oral capsaicin gel in burning mouth syndrome. Acta Odontol. Scand..

[26] Silvestre F.J., Silvestre-Rangil J., Tamarit-Santafé C., Bautista D. (2012). Application of a capsaicin rinse in the treatment of burning mouth syndrome. Med. Oral Patol. Oral Cir. Bucal.

[27] Winocur E., Gazit E., Halachmi M., Eli I., Gaziti E. (2000). Topical Application of Capsaicin for the Treatment of Localized Pain in the Temporomandibular Joint Area. J. Orofac. Pain.

[28] Campbell B.K., Fillingim R.B., Lee S., Brao R., Price D.D., Neubert J.K. (2017). Effects of high-dose capsaicin on TMD subjects: A randomized clinical study. JDR Clin. Trans. Res..

[29] Fusco B.M., Alessandri M. (1992). Analgesic effect of capsaicin in idiopathic trigeminal neuralgia. Anesth. Analg..

[30] Epstein J.B., Marcoe J.H. (1994). Topical application of capsaicin for treatment of oral neuropathic pain and trigeminal neuralgia. Oral Surg. Oral Med. Oral Pathol..

[31] Andrade A.C.M., Molina Esquivel N., Goldschmied Rossel F., Benso B. (2025). TRPV1-target drugs for the treatment of orofacial pain. Front. Pharmacol..

[32] Page M.J., McKenzie J.E., Bossuyt P.M., Boutron I., Hoffmann T.C., Mulrow C.D., Shamseer L., Tetzlaff J.M., Akl E.A., Brennan S.E. (2021). The PRISMA 2020 statement: An updated guideline for reporting systematic reviews. BMJ.

[33] Berger A., Henderson M., Nadoolman W., Duffy V., Cooper D., Saberski L., Bartoshuk L. (1995). Oral capsaicin provides temporary relief for oral mucositis pain secondary to chemotherapy/radiation therapy. J. Pain Symptom Manag..

[34] Militão Ricken C., Natalia Souza de Péder S., Suemi Kamikawa D., Pieralisi N., Chicarelli M., de Souza Tolentino E. (2021). Evaluation of a Protocol For Topical Application of Capsaicine Gel 0.025% in the Management of Burning Mouth Syndrome Correlating its Impact on Quality of Life. Int. J. Odontostomat..

[35] Wagner T., Poole C., Roth-Daniek A. (2013). The Capsaicin 8% Patch for Neuropathic Pain in Clinical Practice: A Retrospective Analysis. Pain Med..

[36] Petruzzi M., Lauritano D., De Benedittis M., Baldoni M., Serpico R. (2004). Systemic capsaicin for burning mouth syndrome: Short-term results of a pilot study. J. Oral Pathol. Med..

[37] Décia Gonçalves M., Virgínia Rebelo M., Paula Barbosa M., Armanda Gomes M. (2020). 8% Capsaicin Patch in Treatment of Peripheral Neuropathic Pain. Pain Physician.

[38] Shuba Y.M. (2021). Beyond Neuronal Heat Sensing: Diversity of TRPV1 Heat-Capsaicin Receptor-Channel Functions. Front. Cell. Neurosci..

[39] Cavalli E., Mammana S., Nicoletti F., Bramanti P., Mazzon E. (2019). The neuropathic pain: An overview of the current treatment and future therapeutic approaches. Int. J. Immunopathol. Pharmacol..

[40] Jaffal S.M. (2023). Role of TRPV1 in Health and Disease. J. Explor. Res. Pharmacol..

[41] Sugimoto T., Xiao C., Ichikawa H. (1998). Neonatal primary neuronal death induced by capsaicin and axotomy involves an apoptotic mechanism. Brain Res..

[42] Abdel-Salam O.M.E., Mózsik G. (2023). Capsaicin, The Vanilloid Receptor TRPV1 Agonist in Neuroprotection: Mechanisms Involved and Significance. Neurochem Res..

[43] Shin C.Y., Shin J., Kim B.M., Wang M.-H., Jang J.-H., Surh Y.-J., Oh U. (2003). Essential role of mitochondrial permeability transition in vanilloid receptor 1-dependent cell death of sensory neurons. Mol. Cell. Neurosci..

[44] Yohana W., Rafisa A. (2024). Unlocking the potential of capsaicin in oral health (Review). Biomed. Rep..

[45] Ahmad N., Chen S., Wang W., Kapila S. (2018). 17β-estradiol Induces MMP-9 and MMP-13 in TMJ Fibrochondrocytes via Estrogen Receptor α. J. Dent. Res..

[46] Zieliński G., Pająk-Zielińska B. (2024). Association between Estrogen Levels and Temporomandibular Disorders: An Updated Systematic Review. Int. J. Mol. Sci..

[47] Jin H.W., Ichikawa H., Fujita M., Yamaai T., Mukae K., Nomura K., Sugimoto T. (2005). Involvement of caspase cascade in capsaicin-induced apoptosis of dorsal root ganglion neurons. Brain Res..

[48] Gibbons C.H., Wang N., Freeman R. (2010). Capsaicin induces degeneration of cutaneous autonomic nerve fibers. Ann. Neurol..

[49] Alalami K., Goff J., Grimson H., Martin O., McDonald E., Mirza T., Mistry D., Ofodile A., Raja S., Shaker T. (2024). Does Topical Capsaicin Affect the Central Nervous System in Neuropathic Pain? A Narrative Review. Pharmaceuticals.

[50] Häggman-Henrikson B., Liv P., Ilgunas A., Visscher C.M., Lobbezoo F., Durham J., Lövgren A. (2020). Increasing gender differences in the prevalence and chronification of orofacial pain in the population. Pain.

[51] Slade G.D., Bair E., Greenspan J.D., Dubner R., Fillingim R.B., Diatchenko L., Maixner W., Knott C., Ohrbach R. (2013). Signs and symptoms of first-onset TMD and sociodemographic predictors of its development: The OPPERA prospective cohort study. J. Pain.

[52] Visscher C., Schouten M., Ligthart L., van Houtem C., de Jongh A., Boomsma D. (2018). Shared Genetics of Temporomandibular Disorder Pain and Neck Pain: Results of a Twin Study. J. Oral Facial Pain Headache.

[53] Bereiter D.A., Thompson R., Rahman M. (2019). Sex differences in estradiol secretion by trigeminal brainstem neurons. Front. Integr. Neurosci..

[54] Penlington C., Araújo-Soares V., Durham J. (2020). Predicting Persistent Orofacial Pain: The Role of Illness Perceptions, Anxiety, and Depression. JDR Clin. Trans. Res..

[55] Edwards R.R., Fillingim R.B., Ness T.J. (2003). Age-related differences in endogenous pain modulation: A comparison of diffuse noxious inhibitory controls in healthy older and younger adults. Pain.

[56] Derafshi R., Ghapanchi J., Rezazadeh F., Sharif D.B., Farzin M. (2019). Prevalence of chronic orofacial pain in elderly patients referred to shiraz dental school from 2005 to 2017. Anesth. Pain Med..

