# Supplementary material for: Capsaicin for Orofacial Pain Management: Systematic Review and Meta-Analysis
Source: Int J Mol Sci. 2026 Aug 7;27(16):7099. doi: 10.3390/ijms27167099 (PMC13513309; doi:10.3390/ijms27167099)
Supplement: Supplementary file 1 [file ijms-27-07099-s001.zip › Supplementary File S1 .pdf]

SUPPLEMENTARY FILES

Supplementary Table S1. The search strategy in electronic databases.

Database	Search Strategy	Number of records identified (February 23, 2025)
**PubMed**	((Facial Pain[MeSH Terms])) OR (Facial Pain[Title/Abstract])) OR (Pain, Facial[Title/Abstract])) OR (Face Pain[Title/Abstract])) OR (Pain, Face[Title/Abstract])) OR (Craniofacial Pain[Title/Abstract])) OR (Pain, Craniofacial[Title/Abstract])) OR (Myofacial Pain[Title/Abstract])) OR (Orofacial Pain[Title/Abstract])) OR (Neuralgic Facial Pain[Title/Abstract])) OR (Facial Neuralgia[MeSH Terms])) OR (Facial Neuralgia[Title/Abstract])) OR (Facial Neuralgias[Title/Abstract])) OR (Neuralgia, Facial[Title/Abstract])) OR (Neuralgias, Facial[Title/Abstract])) OR (Facial Pain Syndromes[Title/Abstract])) OR (Facial Pain Syndrome[Title/Abstract])) OR (Syndrome, Facial Pain[Title/Abstract])) OR (Syndromes, Facial Pain[Title/Abstract])) OR (Craniofacial Pain Syndromes[Title/Abstract])) OR (Craniofacial Pain Syndrome[Title/Abstract])) OR (Sphenopalatine Neuralgia[Title/Abstract])) OR (Neuralgia, Sphenopalatine[Title/Abstract])) OR (Sphenopalatine Neuralgias[Title/Abstract])) OR (Myofacial Pain Syndromes[Title/Abstract])) OR (Myofacial Pain Syndrome[Title/Abstract])) OR (Syndrome, Myofacial Pain[Title/Abstract])) OR (Acute Pain[MeSH Terms])) OR (Acute Pain[Title/Abstract])) OR (Acute Pains[Title/Abstract])) OR (Pain, Acute[Title/Abstract])) OR (Pains, Acute[Title/Abstract])) OR (Chronic Pain[MeSH Terms])) OR (Chronic Pain[Title/Abstract])) OR (Pain, Chronic[Title/Abstract])) OR (Widespread Chronic Pain[Title/Abstract])) OR (Chronic Pain, Widespread[Title/Abstract])) OR (Chronic Primary Pain[Title/Abstract])) OR (Pain, Chronic Primary[Title/Abstract])) OR (Chronic Secondary Pain[Title/Abstract])) OR (Pain, Chronic Secondary[Title/Abstract])) OR (Eye Pain[MeSH Terms])) OR (Eye Pain[Title/Abstract])) OR (Pain, Eye[Title/Abstract])) OR (Pains, Eye[Title/Abstract])) AND (((((((((((((((((((((((TRPV Cation Channels[MeSH Terms])) OR (Cation Channels, TRPV[Title/Abstract])) OR (Channels, TRPV Cation[Title/Abstract])) OR (Capsaicin Receptor[Title/Abstract])) OR (Receptor, Capsaicin[Title/Abstract])) OR (Capsaicin Receptors[Title/Abstract])) OR (Receptors, Capsaicin[Title/Abstract])) OR (Vanilloid Receptor[Title/Abstract])) OR (Receptor, Vanilloid[Title/Abstract])) OR (Vanilloid Receptors[Title/Abstract])) OR (Receptors, Vanilloid[Title/Abstract])) OR (Capsaicin[MeSH Terms])))) OR (Capsaicin[Title/Abstract])) OR (Capsaicine[Title/Abstract])) OR (8-Methyl-N-Vanillyl-6-Nonenamide[Title/Abstract])) OR (Axsain[Title/Abstract])) OR (Capsidol[Title/Abstract])) OR (Zostrix[Title/Abstract])) OR (Capsin[Title/Abstract])) OR (Capzasin[Title/Abstract])) OR (NGX-4010[Title/Abstract]))	1089 articles
**Web of Science**	(TS=("Facial Pain" OR "Pain, Facial" OR "Face Pain" OR "Pain, Face" OR "Craniofacial Pain" OR "Pain, Craniofacial" OR "Myofacial Pain"	1084 articles

	OR “Orofacial Pain” OR “Neuralgic Facial Pain” OR “Facial Neuralgia” OR “Facial Neuralgias” OR “Neuralgia, Facial” OR “Neuralgias, Facial” OR “Facial Pain Syndromes” OR “Facial Pain Syndrome” OR “Syndrome, Facial Pain” OR “Syndromes, Facial Pain” OR “Craniofacial Pain Syndromes” OR “Craniofacial Pain Syndrome” OR “Sphenopalatine Neuralgia” OR “Neuralgia, Sphenopalatine” OR “Sphenopalatine Neuralgias” OR “Myofacial Pain Syndromes” OR “Myofacial Pain Syndrome” OR “Syndrome, Myofacial Pain” OR “Acute Pain” OR “Acute Pains” OR “Pain, Acute” OR “Pains, Acute” OR “Chronic Pain” OR “Pain, Chronic” OR “Widespread Chronic Pain” OR “Chronic Pain, Widespread” OR “Chronic Primary Pain” OR “Pain, Chronic Primary” OR “Chronic Secondary Pain” OR “Pain, Chronic Secondary” OR “Eye Pain” OR “Pain, Eye” OR “Pains, Eye”)) AND (TS=(“TRPV Cation Channels” OR “Cation Channels, TRPV” OR “Channels, TRPV Cation” OR “Capsaicin Receptor” OR “Receptor, Capsaicin” OR “Capsaicin Receptors” OR “Receptors, Capsaicin” OR “Vanilloid Receptor” OR “Receptor, Vanilloid” OR “Vanilloid Receptors” OR “Receptors, Vanilloid” OR “Capsaicin” OR “Capsaicine” OR “8-Methyl-N-Vanillyl-6-Nonenamide” OR “Axsain” OR “Capsidol” OR “Zostrix” OR “Capsin” OR “Capzasin” OR “NGX-4010”))	
**Cochrane**	#1 MeSH descriptor: [Facial Pain] explode all trees 956	150 articles
	#2 ("facial pain"):ti,ab,kw 1317	
	#3 ("Pain, Facial"):ti,ab,kw 82	
	#4 ("Face Pain"):ti,ab,kw 425	
	#5 ("Pain, Face"):ti,ab,kw 37	
	#6 (Craniofacial Pain):ti,ab,kw 155	
	#7 ("Pain, Craniofacial"):ti,ab,kw 0	
	#8 ("Myofacial Pain"):ti,ab,kw 54	
	#9 ("Orofacial Pain"):ti,ab,kw 295	
	#10 ("Neuralgic Facial Pain"):ti,ab,kw 0	
	#11 MeSH descriptor: [Facial Neuralgia] explode all trees 390	
	#12 ("Facial Neuralgia"):ti,ab,kw 49	
	#13 ("Facial Neuralgias"):ti,ab,kw 3	
	#14 ("Neuralgia, Facial"):ti,ab,kw 1	
	#15 ("Neuralgias, Facial"):ti,ab,kw 0	
	#16 ("Facial Pain Syndromes"):ti,ab,kw 5	
	#17 ("Facial Pain Syndrome"):ti,ab,kw7	
	#18 ("Syndrome, Facial Pain"):ti,ab,kw 3	
	#19 ("Syndromes, Facial Pain"):ti,ab,kw 0	
	#20 ("Craniofacial Pain Syndromes"):ti,ab,kw 2	
	#21 ("Craniofacial Pain Syndrome"):ti,ab,kw 1	
	#22 ("Sphenopalatine Neuralgia"):ti,ab,kw 1	
	#23 ("Neuralgia, Sphenopalatine"):ti,ab,kw 0	
	#24 ("Sphenopalatine Neuralgias"):ti,ab,kw 0	
	#25 ("Myofacial Pain Syndromes"):ti,ab,kw 1	
	#26 ("Myofacial Pain Syndrome"):ti,ab,kw 20	
	#27 ("Syndrome, Myofacial Pain"):ti,ab,kw 0	
	#28 MeSH descriptor: [Acute Pain] explode all trees 1368	
	#29 ("Acute Pain"):ti,ab,kw 4647	
	#30 ("Acute Pains"):ti,ab,kw 10	
	#31 ("Pain, Acute"):ti,ab,kw 442	
	#32 ("Pains, Acute"):ti,ab,kw 1	

	#33 MeSH descriptor: [Chronic Pain] explode all trees 4850<br>#34 ("Chronic Pain"):ti,ab,kw 14606<br>#35 ("Pain, Chronic"):ti,ab,kw 492<br>#36 ("Widespread Chronic Pain"):ti,ab,kw 27<br>#37 ("Chronic Pain, Widespread"):ti,ab,kw 1<br>#38 ("Chronic Primary Pain"):ti,ab,kw 13<br>#39 ("Pain, Chronic Primary"):ti,ab,kw 2<br>#40 ("Chronic Secondary Pain"):ti,ab,kw 1<br>#41 ("Pain, Chronic Secondary"):ti,ab,kw 0<br>#42 MeSH descriptor: [Eye Pain] explode all trees 161<br>#43 ("Eye Pain"):ti,ab,kw 784<br>#44 ("Pain, Eye"):ti,ab,kw 64<br>#45 ("Pains, Eye"):ti,ab,kw 0<br>#46 #1 or #2 or #3 or #4 or #5 or #6 or #7 or #8 or #9 or #10 or #11 or #12 or #13 or #14 or #15 or #16 or #17 or #18 or #19 or #20 or #21 or #22 or #23 or #24 or #25 or #26 or #27 or #28 or #29 or #30 or #31 or #32 or #33 or #34 or #35 or #36 or #37 or #38 or #39 or #40 or #41 or #42 or #43 or #44 or #45 22001<br>#47 MeSH descriptor: [Capsaicin] explode all trees 802<br>#48 ("Capsaicin"):ti,ab,kw 1597<br>#49 ("Capsaicine"):ti,ab,kw 9<br>#50 ("8-Methyl-N-Vanillyl-6-Nonenamide"):ti,ab,kw 5<br>#51 ("Axsain"):ti,ab,kw 3<br>#52 ("Capsidol"):ti,ab,kw 0<br>#53 ("Zostrix"):ti,ab,kw 5<br>#54 ("Capsin"):ti,ab,kw 0<br>#55 ("Capzasin"):ti,ab,kw 1<br>#56 (NGX-4010):ti,ab,kw 34<br>#57 MeSH descriptor: [TRPV Cation Channels] explode all trees 94<br>#58 ("Cation Channels, TRPV"):ti,ab,kw 0<br>#59 ("Channels, TRPV Cation"):ti,ab,kw 0<br>#60 ("Capsaicin Receptor"):ti,ab,kw 11<br>#61 ("Receptor, Capsaicin"):ti,ab,kw 0<br>#62 ("Capsaicin Receptors"):ti,ab,kw 2<br>#63 ("Receptors, Capsaicin"):ti,ab,kw 2<br>#64 ("Vanilloid Receptor"):ti,ab,kw 34<br>#65 ("Receptor, Vanilloid"):ti,ab,kw 5<br>#66 ("Vanilloid Receptors"):ti,ab,kw 8<br>#67 ("Receptors, Vanilloid"):ti,ab,kw 1<br>#68 #47 or #48 or #49 or #50 or #51 or #52 or #53 or #54 or #55 or #56 or #57 or #58 or #59 or #60 or #61 or #62 or #63 or #64 or #65 or #66 or #67 1662<br>#69 #46 and #68 150	
**SCOPUS**	TITLE-ABS-KEY ("facial pain") OR TITLE-ABS-KEY ("pain, facial") OR TITLE-ABS-KEY ("face pain") OR TITLE-ABS-KEY ("pain, face") OR TITLE-ABS-KEY ("craniofacial pain") OR TITLE-ABS-KEY ("pain, craniofacial") OR TITLE-ABS-KEY ("myofacial pain") OR TITLE-ABS-KEY ("orofacial pain") OR TITLE-ABS-KEY ("neuralgic facial pain") OR TITLE-ABS-KEY ("facial neuralgia") OR TITLE-ABS-KEY ("facial neuralgias") OR TITLE-ABS-KEY	2608 articles

	("neuralgia, facial") OR TITLE-ABS-KEY ("neuralgias, facial") OR TITLE-ABS-KEY ("facial pain syndromes") OR TITLE-ABS-KEY ("facial pain syndrome") OR TITLE-ABS-KEY ("syndrome, facial pain") OR TITLE-ABS-KEY ("syndromes, facial pain") OR TITLE-ABS-KEY ("craniofacial pain syndromes") OR TITLE-ABS-KEY ("craniofacial pain syndrome") OR TITLE-ABS-KEY ("Sphenopalatine Neuralgia") OR TITLE-ABS-KEY ("Neuralgia, Sphenopalatine") OR TITLE-ABS-KEY ("sphenopalatine neuralgias") OR TITLE-ABS-KEY ("myofacial pain syndrome") OR TITLE-ABS-KEY ("myofacial pain syndrome") OR TITLE-ABS-KEY ("syndrome, myofacial pain") OR TITLE-ABS-KEY ("acute pain") OR TITLE-ABS-KEY ("Acute Pains") OR TITLE-ABS-KEY ("pain, acute") OR TITLE-ABS-KEY ("pains, acute") OR TITLE-ABS-KEY ("chronic pain") OR TITLE-ABS-KEY ("pain, chronic") OR TITLE-ABS-KEY ("widespread chronic pain") OR TITLE-ABS-KEY ("chronic pain, widespread") OR TITLE-ABS-KEY ("chronic primary pain") OR TITLE-ABS-KEY ("pain, chronic primary") OR TITLE-ABS-KEY ("chronic secondary pain") OR TITLE-ABS-KEY ("pain, chronic secondary") OR TITLE-ABS-KEY ("eye pain") OR TITLE-ABS-KEY ("pain, eye") OR TITLE-ABS-KEY ("pains, eye") AND TITLE-ABS-KEY ("TRPV Cation Channels") OR TITLE-ABS-KEY ("Cation Channels, TRPV") OR TITLE-ABS-KEY ("Channels, TRPV Cation") OR TITLE-ABS-KEY ("Capsaicin Receptor") OR TITLE-ABS-KEY ("Receptor, Capsaicin") OR TITLE-ABS-KEY ("Capsaicin Receptors") OR TITLE-ABS-KEY ("Receptors, Capsaicin") OR TITLE-ABS-KEY ("Vanilloid Receptor") OR TITLE-ABS-KEY ("Receptor, Vanilloid") OR TITLE-ABS-KEY ("Vanilloid Receptors") OR TITLE-ABS-KEY ("Receptors, Vanilloid") OR TITLE-ABS-KEY ("Capsaicin") OR TITLE-ABS-KEY ("Capsaicine") OR TITLE-ABS-KEY ("8-Methyl-N-Vanillyl-6-Nonenamide") OR TITLE-ABS-KEY ("Axsain") OR TITLE-ABS-KEY ("Capsidol") OR TITLE-ABS-KEY ("Zostrix") OR TITLE-ABS-KEY ("Capsin") OR TITLE-ABS-KEY ("Capzasin") OR TITLE-ABS-KEY ("NGX-4010")	
**LILACS**	(mh:("Facial Pain")) OR (mh:("Facial Neuralgia")) OR (mh:("Acute Pain")) OR (mh:("Chronic Pain")) OR (mh:("Eye Pain")) OR (tw:("Pain, Facial")) OR (tw:("Face Pain")) OR (tw:("Pain, Face")) OR (tw:("Craniofacial Pain")) OR (tw:("Pain, Craniofacial")) OR (tw:("Myofacial Pain")) OR (tw:("Orofacial Pain")) OR (tw:("Neuralgic Facial Pain")) OR (tw:("Facial Neuralgia")) OR (tw:("Facial Neuralgias")) OR (tw:("Neuralgia, Facial")) OR (tw:("Neuralgias, Facial")) OR (tw:("Facial Pain Syndromes")) OR (tw:("Facial Pain Syndrome")) OR (tw:("Syndrome, Facial Pain")) OR (tw:("Syndromes, Facial Pain")) OR (tw:("Craniofacial Pain Syndromes")) OR (tw:("Craniofacial Pain Syndrome")) OR (tw:("Sphenopalatine Neuralgia")) OR (tw:("Neuralgia, Sphenopalatine")) OR (tw:("Sphenopalatine Neuralgias")) OR (tw:("Myofacial Pain Syndromes")) OR (tw:("Myofacial Pain Syndrome")) OR (tw:("Syndrome, Myofacial Pain")) OR (tw:("Acute Pain")) OR (tw:("Acute Pains")) OR (tw:("Pain, Acute")) OR (tw:("Pains, Acute")) OR (tw:("Chronic Pain")) OR (tw:("Pain, Chronic")) OR (tw:("Widespread Chronic Pain")) OR (tw:("Chronic Pain, Widespread")) OR (tw:("Chronic Primary Pain")) OR (tw:("Pain, Chronic Primary")) OR (tw:("Chronic Secondary Pain")) OR (tw:("Pain, Chronic	125 articles

	Secondary")) OR (tw:("Eye Pain")) OR (tw:("Pain, Eye") OR (tw:("Pains, Eye")) AND (mh:("TRPV Cation Channels")) OR (mh:("Capsaicin")) OR (tw:("TRPV Cation Channels")) OR (tw:("Cation Channels, TRPV")) OR (tw:("Channels, TRPV Cation")) OR (tw:("Capsaicin Receptor")) OR (tw:("Receptor, Capsaicin")) OR (tw:("Capsaicin Receptors")) OR (tw:("Receptors, Capsaicin")) OR (tw:("Vanilloid Receptor")) OR (tw:("Receptor, Vanilloid")) OR (tw:("Vanilloid Receptors")) OR (tw:("Receptors, Vanilloid")) OR (tw:("Capsaicin")) OR (tw:("Capsaicine")) OR (tw:("8-Methyl-N-Vanillyl-6-Nonenamide")) OR (tw:("Axsain")) OR (tw:("Capsidol")) OR (tw:("Zostrix")) OR (tw:("Capsin")) OR (tw:("Capzasin")) OR (tw:("NGX-4010"))
