# Supplementary material for: Capsaicin for Orofacial Pain Management: Systematic Review and Meta-Analysis
Source: Int J Mol Sci. 2026 Aug 7;27(16):7099. doi: 10.3390/ijms27167099 (PMC13513309; doi:10.3390/ijms27167099)
Supplement: Supplementary file 1 [file ijms-27-07099-s001.zip › Supplementary File S2.pdf]

SUPPLEMENTARY FILES

Supplementary Table S2. Risk of bias of the included randomized controlled trials, including the crossover trial, assessed using the Cochrane RoB 2 tool.

**Ref or Label**	Jorgensen et al. 2016 [25]	**Aim**	assignment to intervention (the 'intention-to-treat' effect)		
**Experimental**	Capsaicin 0.025%	**Comparator**	Capsaicin 0.01%	**Source**	Journal article(s)
**Outcome**	Orofacial pain Reduction	**Results**	Mean Difference = 0.46	**Weight**	16

**Domain**	**Signalling question**	**Response**	**Comments**
**Bias arising from the randomization process**	1.1 Was the allocation sequence random?	Y	The study is described as a randomized, double-blind, cross-over trial, and the allocation list was computer-generated. The computer-generated randomization list was prepared by an impartial person from Glostrup Pharmacy. The gels were provided as Tube 1 and Tube 2, and both investigators and patients were blinded to which tube contained each concentration.
	1.2 Was the allocation sequence concealed until participants were enrolled and assigned to interventions?	Y	
	1.3 Did baseline differences between intervention groups suggest a problem with the randomization process?	Y	Although the patients were randomly assigned, the authors reported a baseline difference in VAS scores between the two treatment-sequence groups.

	**Risk of bias judgement**	**Low**	The study is described as a randomized, double-blind, cross-over trial, and the allocation list was computer-generated. The computer-generated randomization list was prepared by an impartial person from Glostrup Pharmacy. The gels were provided as Tube 1 and Tube 2, and both investigators and patients were blinded to which tube contained each concentration.<br><br>Although the patients were randomly assigned, the authors reported a baseline difference in VAS scores between the two treatment-sequence groups.
**Domain S: Risk of bias arising from period and carryover effects**	S.1 Was the number of participants allocated to each of the two sequences equal or nearly equal?	Y	Twenty-two participants were randomized, with 11 allocated to each treatment sequence (0.01%-0.025% and 0.025%-0.01%).
	S.2 If N/PN/NI to S.1: Were period effects accounted for in the analysis?	NA	
	S.3 Was there sufficient time for any carryover effects to have disappeared before outcome assessment in the second period?	PY	A 14-day washout period was included between treatment periods. In addition, the authors formally assessed carryover using a mixed model including the period-by-treatment interaction and reported no significant carryover effect (p = 0.23). However, because capsaicin may produce prolonged desensitization of TRPV1 receptors beyond the immediate treatment period, some uncertainty remains regarding whether a 14-day washout was fully sufficient.
	**Risk of bias judgement**	**Low**	Twenty-two participants were randomized, with 11 allocated to each treatment sequence (0.01%-0.025% and 0.025%-0.01%).<br><br>A 14-day washout period was included between treatment periods. In addition, the authors formally assessed carryover using a mixed model including the period-by-treatment interaction and reported

			no significant carryover effect (p = 0.23). However, because capsaicin may produce prolonged desensitization of TRPV1 receptors beyond the immediate treatment period, some uncertainty remains regarding whether a 14-day washout was fully sufficient.
**Bias due to deviations from intended interventions**	2.1.Were participants aware of their assigned intervention during the trial?	N	Both treatment periods involved active capsaicin gels. Although the concentrations differed, both formulations produced the characteristic burning sensation associated with capsaicin, making it unlikely that participants could reliably identify which concentration they were receiving.
	2.2.Were carers and people delivering the interventions aware of participants' assigned intervention during the trial?	N	Investigators were blinded to treatment allocation, and both interventions consisted of active capsaicin gels with similar expected local effects. There is no evidence that investigators became aware of the assigned concentration during either treatment period.
	2.3. If Y/PY/NI to 2.1 or 2.2: Were there deviations from the intended intervention that arose because of the experimental context?	NA	
	2.4 If Y/PY to 2.3: Were these deviations likely to have affected the outcome?	NA	
	2.5. If Y/PY/NI to 2.4: Were these deviations from intended intervention balanced between groups?	NA	
	2.6 Was an appropriate analysis used to estimate the effect of assignment to intervention?	PN	Four participants who discontinued treatment because of adverse effects were excluded from the final analysis. The analysis was performed only on the 18 participants who completed the study, rather than including all randomized participants.
	2.7 If N/PN/NI to 2.6: Was there potential for a substantial impact (on the result) of the failure to analyse participants in the group to which they were randomized?	PY	Four of the 22 randomized participants (18%) were excluded because of adverse effects related to the intervention. Since withdrawals were intervention-related and the sample size was

			small, excluding these participants may have substantially influenced the estimated treatment effect.
	**Risk of bias judgement**	**High**	Both treatment periods involved active capsaicin gels. Although the concentrations differed, both formulations produced the characteristic burning sensation associated with capsaicin, making it unlikely that participants could reliably identify which concentration they were receiving.<br><br>Investigators were blinded to treatment allocation, and both interventions consisted of active capsaicin gels with similar expected local effects. There is no evidence that investigators became aware of the assigned concentration during either treatment period.<br><br>Four participants who discontinued treatment because of adverse effects were excluded from the final analysis. The analysis was performed only on the 18 participants who completed the study, rather than including all randomized participants.<br><br>Four of the 22 randomized participants (18%) were excluded because of adverse effects related to the intervention. Since withdrawals were intervention-related and the sample size was small, excluding these participants may have substantially influenced the estimated treatment effect.

**Bias due to missing outcome data**	3.1 Were data for this outcome available for all, or nearly all, participants randomized?	N	Outcome data were not available for all randomized participants. Four of the 22 randomized participants discontinued the study before completing both treatment periods and were excluded from the final analysis.
	3.2 If N/PN/NI to 3.1: Is there evidence that result was not biased by missing outcome data?	N	No intention-to-treat analysis, sensitivity analysis, or imputation methods were reported to demonstrate that the missing outcome data did not bias the results.
	3.3 If N/PN to 3.2: Could missingness in the outcome depend on its true value?	PY	Participants discontinued because of adverse effects (burning sensation), which may be associated with their response to treatment and could therefore be related to the true outcome.<br><br>Since withdrawals were directly related to the intervention and represented approximately 18% of the randomized sample, it is plausible that missing outcome data depended on participants' treatment experience, potentially influencing the estimated treatment effect.
	3.4 If Y/PY/NI to 3.3: Is it likely that missingness in the outcome depended on its true value?	PY	
	**Risk of bias judgement**	**High**	Outcome data were not available for all randomized participants. Four of the 22 randomized participants discontinued the study before completing both treatment periods and were excluded from the final analysis.<br><br>No intention-to-treat analysis, sensitivity analysis, or imputation methods were reported to demonstrate that the missing outcome data did not bias the results.<br><br>Participants discontinued because of adverse effects (burning sensation), which may be associated with their response to treatment and could therefore be related to the true outcome.<br><br>Since withdrawals were directly related to the intervention and represented approximately 18% of the randomized sample, it is

			plausible that missing outcome data depended on participants' treatment experience, potentially influencing the estimated treatment effect.
**Bias in measurement of the outcome**	4.1 Was the method of measuring the outcome inappropriate?	N	Pain intensity was measured using a validated Visual Analogue Scale (VAS), which is an appropriate and widely accepted instrument for assessing burning mouth symptoms.
	4.2 Could measurement or ascertainment of the outcome have differed between intervention groups?	N	The same outcome measurement procedures, assessment schedule, and instrument were applied during both treatment periods for all participants.
	4.3 Were outcome assessors aware of the intervention received by study participants?	N	Pain was self-reported by participants. Both interventions consisted of active capsaicin gels, and the study was conducted under double-blind conditions. Although the higher concentration produced somewhat greater burning sensations, both gels caused similar local effects, making reliable identification of the treatment concentration unlikely.
	4.4 If Y/PY/NI to 4.3: Could assessment of the outcome have been influenced by knowledge of intervention received?	NA	
	4.5 If Y/PY/NI to 4.4: Is it likely that assessment of the outcome was influenced by knowledge of intervention received?	NA	
	**Risk of bias judgement**	**Low**	Pain intensity was measured using a validated Visual Analogue Scale (VAS), which is an appropriate and widely accepted instrument for assessing burning mouth symptoms.<br><br>The same outcome measurement procedures, assessment schedule, and instrument were applied during both treatment periods for all participants.<br><br>Pain was self-reported by participants. Both interventions consisted of active capsaicin gels, and the study was conducted

			under double-blind conditions. Although the higher concentration produced somewhat greater burning sensations, both gels caused similar local effects, making reliable identification of the treatment concentration unlikely.
**Bias in selection of the reported result**	5.1 Were the data that produced this result analysed in accordance with a pre-specified analysis plan that was finalized before unblinded outcome data were available for analysis?	NI	Although the article states that the trial was registered at ClinicalTrials.gov, the registration record could not be identified or verified. Therefore, it cannot be determined whether the analyses followed a pre-specified analysis plan finalized before unblinded outcome data were available.<br><br>5.2 No (N)<br><br>5.3 No Information (NI)<br><br>5.4 No (N)
	5.2 ... multiple eligible outcome measurements (e.g. scales, definitions, time points) within the outcome domain?	N	The primary outcome (VAS pain score) and assessment time points described in the Methods were consistently reported in the Results, with no evidence of selective outcome measurement reporting.
	5.3 ... multiple eligible analyses of the data?	NI	There is insufficient information to determine whether multiple eligible statistical analyses were performed before selecting the reported analysis.
	5.4 Is a result based on data from both periods sought, but unavailable on the basis of carryover having been identified?	N	Results from both treatment periods were analysed, and second-period data were not excluded because of carryover. The authors tested for carryover and found no statistically significant effect.
	**Risk of bias judgement**	**Some concerns**	Although the article states that the trial was registered at ClinicalTrials.gov, the registration record could not be identified or verified. Therefore, it cannot be determined whether the analyses followed a pre-specified analysis plan finalized before unblinded outcome data were available.

			5.2 No (N)<br><br>5.3 No Information (NI)<br><br>5.4 No (N)<br><br>The primary outcome (VAS pain score) and assessment time points described in the Methods were consistently reported in the Results, with no evidence of selective outcome measurement reporting.<br><br>There is insufficient information to determine whether multiple eligible statistical analyses were performed before selecting the reported analysis.<br><br>Results from both treatment periods were analysed, and second-period data were not excluded because of carryover. The authors tested for carryover and found no statistically significant effect.
**Overall bias**	**Risk of bias judgement**	**High**	
