# Supplementary material for: Capsaicin for Orofacial Pain Management: Systematic Review and Meta-Analysis
Source: Int J Mol Sci. 2026 Aug 7;27(16):7099. doi: 10.3390/ijms27167099 (PMC13513309; doi:10.3390/ijms27167099)
Supplement: Supplementary file 1 [file ijms-27-07099-s001.zip › Supplementary File S3.pdf]

SUPPLEMENTARY FILES

Supplementary Table S3. Risk of bias assessment of the included randomized controlled trials using the Cochrane RoB 2 tool.

**Ref or Label**	Winocur et al. 2000 [27]	**Aim**	assignment to intervention (the 'intention-to-treat' effect)		
**Experimental**	Treatment	**Comparator**	Control	**Source**	Journal article(s)
**Outcome**	orofacial pain reduction	**Results**	Mean Difference = -1.10	**Weight**	1

**Domain**	**Signalling question**	**Response**	**Comments**
**Bias arising from the randomization process**	1.1 Was the allocation sequence random?	PY	The study was reported as randomized, but no information was provided on how the random allocation sequence was generated or whether allocation was concealed before participant assignment. Consequently, the randomization process could not be fully verified.
	1.2 Was the allocation sequence concealed until participants were enrolled and assigned to interventions?	NI	
	1.3 Did baseline differences between intervention groups suggest a problem with the randomization process?	N	Baseline demographic and clinical characteristics were generally comparable between the intervention groups. No important imbalances were observed that would suggest a problem with the randomization process.
	**Risk of bias judgement**	**Some concerns**	No information was provided on how the random allocation sequence was generated.

			No important imbalances were observed that would suggest a problem with the randomization process.
**Bias due to deviations from intended interventions**	2.1.Were participants aware of their assigned intervention during the trial?	PN	The study was described as double-blind. Participants were informed that either cream could produce a warm, stinging, or burning sensation, which was intended to preserve blinding. Although the characteristic burning sensation induced by capsaicin may have allowed some participants and evaluaters to infer their treatment allocation.
	2.2.Were carers and people delivering the interventions aware of participants' assigned intervention during the trial?	PN	
	2.3. If Y/PY/NI to 2.1 or 2.2: Were there deviations from the intended intervention that arose because of the experimental context?	NA	
	2.4 If Y/PY to 2.3: Were these deviations likely to have affected the outcome?	NA	
	2.5. If Y/PY/NI to 2.4: Were these deviations from intended intervention balanced between groups?	NA	
	2.6 Was an appropriate analysis used to estimate the effect of assignment to intervention?	N	Dois participantes do grupo capsaicina interromperam o tratamento devido à sensação intensa de queimação e foram excluídos da análise. O estudo analisou apenas os participantes que completaram o protocolo (análise por protocolo), e não por intenção de tratar (ITT).
	2.7 If N/PN/NI to 2.6: Was there potential for a substantial impact (on the result) of the failure to analyse participants in the group to which they were randomized?	PN	Embora a análise não tenha sido por ITT, apenas 2 de 30 participantes (6,7%) foram excluídos. Essa perda é relativamente pequena e provavelmente teve impacto limitado na estimativa do efeito. Entretanto, como ambas as perdas ocorreram no grupo capsaicina, existe algum potencial de viés, mas provavelmente não substancial.
	**Risk of bias judgement**	**Low**	
**Bias due to missing outcome data**	3.1 Were data for this outcome available for all, or nearly all, participants randomized?	N	Outcome data were not available for all randomized participants. Two participants in the capsaicin group discontinued the study because of intolerable burning sensations and were excluded from the analysis.

	3.2 If N/PN/NI to 3.1: Is there evidence that result was not biased by missing outcome data?	N	No sensitivity analysis, intention-to-treat analysis, or other methods were reported to demonstrate that the missing outcome data did not bias the results.
	3.3 If N/PN to 3.2: Could missingness in the outcome depend on its true value?	PY	Participants withdrew because of intolerance to the intervention (burning sensation), suggesting that missingness may have been related to treatment response or outcome severity.
	3.4 If Y/PY/NI to 3.3: Is it likely that missingness in the outcome depended on its true value?	PY	
	**Risk of bias judgement**	**Some concerns**	Outcome data were not available for all randomized participants. Two participants in the capsaicin group discontinued the study because of intolerable burning sensations and were excluded from the analysis.<br><br>No sensitivity analysis, intention-to-treat analysis, or other methods were reported to demonstrate that the missing outcome data did not bias the results.<br><br>Participants withdrew because of intolerance to the intervention (burning sensation), suggesting that missingness may have been related to treatment response or outcome severity.
**Bias in measurement of the outcome**	4.1 Was the method of measuring the outcome inappropriate?	N	Pain was assessed using a validated Visual Analogue Scale (VAS), and clinical outcomes were measured using standardized examination procedures. These methods are appropriate for assessing pain in temporomandibular disorders.
	4.2 Could measurement or ascertainment of the outcome have differed between intervention groups?	N	The same outcome measures and assessment procedures were applied equally to both groups throughout the study.
	4.3 Were outcome assessors aware of the intervention received by study participants?	PY	Although the study was described as double-blind, the characteristic burning sensation produced by topical capsaicin may have enabled participants, who were the outcome assessors for the self-reported pain outcome, to infer their treatment allocation.
	4.4 If Y/PY/NI to 4.3: Could assessment of the outcome have been influenced by knowledge of intervention received?	PN	Although participants may have inferred their treatment allocation because of the characteristic burning sensation associated with

	4.5 If Y/PY/NI to 4.4: Is it likely that assessment of the outcome was influenced by knowledge of intervention received?	NA	capsaicin, knowledge of the assigned intervention was unlikely to systematically influence outcome assessment. The burning sensation is a pharmacological effect of capsaicin and may transiently increase pain rather than indicate therapeutic benefit. Therefore, any influence of treatment awareness on self-reported pain is considered unlikely.
	**Risk of bias judgement**	**Low**	Pain was assessed using a validated Visual Analogue Scale (VAS), and clinical outcomes were measured using standardized examination procedures. These methods are appropriate for assessing pain in temporomandibular disorders.<br><br>The same outcome measures and assessment procedures were applied equally to both groups throughout the study.<br><br>Although the study was described as double-blind, the characteristic burning sensation produced by topical capsaicin may have enabled participants, who were the outcome assessors for the self-reported pain outcome, to infer their treatment allocation.<br><br>Although participants may have inferred their treatment allocation because of the characteristic burning sensation associated with capsaicin, knowledge of the assigned intervention was unlikely to systematically influence outcome assessment. The burning sensation is a pharmacological effect of capsaicin and may transiently increase pain rather than indicate therapeutic benefit. Therefore, any influence of treatment awareness on self-reported pain is considered unlikely.
**Bias in selection of the reported result**	5.1 Were the data that produced this result analysed in accordance with a pre-specified analysis plan that was finalized before unblinded outcome data were available for analysis?	N	No study protocol, statistical analysis plan, or trial registration was available to determine whether the reported analyses were pre-specified before outcome data became available.
	5.2 ... multiple eligible outcome measurements (e.g. scales, definitions, time points) within the outcome domain?	N	The study clearly prespecified the clinical outcomes (VAS pain scores, pain relief, palpation findings, and mouth opening) and reported the scheduled weekly assessments. There was no

			evidence that only selected outcome measurements were reported.
	5.3 ... multiple eligible analyses of the data?	N	The article does not provide sufficient information to determine whether multiple statistical analyses were performed before selecting those reported. No protocol or statistical analysis plan was available.
	**Risk of bias judgement**	**Some concerns**	No study protocol, statistical analysis plan, or trial registration was available to determine whether the reported analyses were pre-specified before outcome data became available.<br><br>The study clearly prespecified the clinical outcomes (VAS pain scores, pain relief, palpation findings, and mouth opening) and reported the scheduled weekly assessments. There was no evidence that only selected outcome measurements were reported.<br><br>The article does not provide sufficient information to determine whether multiple statistical analyses were performed before selecting those reported. No protocol or statistical analysis plan was available.
**Overall bias**	**Risk of bias judgement**	**Some concerns**	No information was provided on how the random allocation sequence was generated.<br><br>No important imbalances were observed that would suggest a problem with the randomization process.<br><br>Outcome data were not available for all randomized participants. Two participants in the capsaicin group discontinued the study because of intolerable burning sensations and were excluded from the analysis.<br><br>No sensitivity analysis, intention-to-treat analysis, or other methods were reported to demonstrate that the missing outcome data did

			not bias the results.<br><br>Participants withdrew because of intolerance to the intervention (burning sensation), suggesting that missingness may have been related to treatment response or outcome severity.<br><br><br>Pain was assessed using a validated Visual Analogue Scale (VAS), and clinical outcomes were measured using standardized examination procedures. These methods are appropriate for assessing pain in temporomandibular disorders.<br><br>The same outcome measures and assessment procedures were applied equally to both groups throughout the study.<br><br>Although the study was described as double-blind, the characteristic burning sensation produced by topical capsaicin may have enabled participants, who were the outcome assessors for the self-reported pain outcome, to infer their treatment allocation.<br><br>Although participants may have inferred their treatment allocation because of the characteristic burning sensation associated with capsaicin, knowledge of the assigned intervention was unlikely to systematically influence outcome assessment. The burning sensation is a pharmacological effect of capsaicin and may transiently increase pain rather than indicate therapeutic benefit. Therefore, any influence of treatment awareness on self-reported pain is considered unlikely.<br><br><br>No study protocol, statistical analysis plan, or trial registration was available to determine whether the reported analyses were pre-specified before outcome data became available.

					The study clearly prespecified the clinical outcomes (VAS pain scores, pain relief, palpation findings, and mouth opening) and reported the scheduled weekly assessments. There was no evidence that only selected outcome measurements were reported.<br><br>The article does not provide sufficient information to determine whether multiple statistical analyses were performed before selecting those reported. No protocol or statistical analysis plan was available.
**Ref or Label**	Petruzzi et al. 2004 [36]	**Aim**	assignment to intervention (the 'intention-to-treat' effect)		
**Experimental**	Treatment	**Comparator**	Control	**Source**	Journal article(s)
**Outcome**	orofacial pain reduction	**Results**	Mean Difference = -1.10	**Weight**	1

Domain	Signalling question	Response	Comments
**Bias arising from the randomization process**	1.1 Was the allocation sequence random?	N	Participants were allocated alternately to the capsaicin or placebo groups, which does not constitute a truly random allocation sequence. Because allocation was performed alternately, treatment assignment was predictable and allocation concealment was not ensured.
	1.2 Was the allocation sequence concealed until participants were enrolled and assigned to interventions?	N	
	1.3 Did baseline differences between intervention groups suggest a problem with the randomization process?	N	Baseline demographic and clinical characteristics appeared comparable between groups, with no important imbalances suggesting problems beyond the non-random allocation method.

	**Risk of bias judgement**	**High**	Participants were allocated alternately to the capsaicin or placebo groups, which does not constitute a truly random allocation sequence. Because allocation was performed alternately, treatment assignment was predictable and allocation concealment was not ensured.<br><br>Baseline demographic and clinical characteristics appeared comparable between groups, with no important imbalances suggesting problems beyond the non-random allocation method.
**Bias due to deviations from intended interventions**	2.1.Were participants aware of their assigned intervention during the trial?	PN	The study was described as triple-blind and placebo tablets were matched in appearance, taste, smell, and color. However, gastrointestinal adverse effects experienced by participants receiving capsaicin may have allowed some participants to infer their treatment allocation.<br><br>Investigators and personnel administering the intervention were reported to be blinded to treatment allocation.
	2.2.Were carers and people delivering the interventions aware of participants' assigned intervention during the trial?	N	
	2.3. If Y/PY/NI to 2.1 or 2.2: Were there deviations from the intended intervention that arose because of the experimental context?	NA	
	2.4 If Y/PY to 2.3: Were these deviations likely to have affected the outcome?	NA	
	2.5. If Y/PY/NI to 2.4: Were these deviations from intended intervention balanced between groups?	NA	
	2.6 Was an appropriate analysis used to estimate the effect of assignment to intervention?	Y	Yes (Y)<br><br>The study reported an intention-to-treat analysis and evaluated participants according to the planned intervention.
	2.7 If N/PN/NI to 2.6: Was there potential for a substantial impact (on the result) of the failure to analyse participants in the group to which they were randomized?	NA	

	**Risk of bias judgement**	**Low**	
**Bias due to missing outcome data**	3.1 Were data for this outcome available for all, or nearly all, participants randomized?	Y	Outcome data were available for all, or nearly all, randomized participants. No relevant loss to follow-up was reported.
	3.2 If N/PN/NI to 3.1: Is there evidence that result was not biased by missing outcome data?	NA	
	3.3 If N/PN to 3.2: Could missingness in the outcome depend on its true value?	NA	
	3.4 If Y/PY/NI to 3.3: Is it likely that missingness in the outcome depended on its true value?	NA	
	**Risk of bias judgement**	**Low**	Outcome data were available for all, or nearly all, randomized participants. No relevant loss to follow-up was reported.
**Bias in measurement of the outcome**	4.1 Was the method of measuring the outcome inappropriate?	N	Pain severity was assessed using a validated Visual Analogue Scale (VAS), which is an appropriate method for evaluating burning mouth symptoms.
	4.2 Could measurement or ascertainment of the outcome have differed between intervention groups?	N	Outcome assessment procedures were identical for both intervention groups.
	4.3 Were outcome assessors aware of the intervention received by study participants?	PY	Gastrointestinal adverse effects associated with capsaicin may have enabled some participants and evaluaters to infer their treatment allocation.
	4.4 If Y/PY/NI to 4.3: Could assessment of the outcome have been influenced by knowledge of intervention received?	PN	Although some participants may have suspected their assigned intervention, gastrointestinal adverse effects do not necessarily indicate therapeutic benefit and are unlikely to have systematically influenced self-reported pain scores.
	4.5 If Y/PY/NI to 4.4: Is it likely that assessment of the outcome was influenced by knowledge of intervention received?	NA	
	**Risk of bias judgement**	**Low**	Pain severity was assessed using a validated Visual Analogue Scale (VAS), which is an appropriate method for evaluating burning mouth symptoms.

			Outcome assessment procedures were identical for both intervention groups.<br><br>Gastrointestinal adverse effects associated with capsaicin may have enabled some participants and evaluaters to infer their treatment allocation.<br><br>Although some participants may have suspected their assigned intervention, gastrointestinal adverse effects do not necessarily indicate therapeutic benefit and are unlikely to have systematically influenced self-reported pain scores.
**Bias in selection of the reported result**	5.1 Were the data that produced this result analysed in accordance with a pre-specified analysis plan that was finalized before unblinded outcome data were available for analysis?	N	No study protocol, trial registration, or pre-specified statistical analysis plan was available.
	5.2 ... multiple eligible outcome measurements (e.g. scales, definitions, time points) within the outcome domain?	N	The predefined outcome (pain severity measured by VAS) was consistently described in the Methods and reported in the Results. There was no evidence of selective reporting among multiple outcome measurements.
	5.3 ... multiple eligible analyses of the data?	N	No information was available to determine whether multiple eligible statistical analyses were conducted before selecting the reported results.
	**Risk of bias judgement**	**Some concerns**	No study protocol, trial registration, or pre-specified statistical analysis plan was available.<br><br>The predefined outcome (pain severity measured by VAS) was consistently described in the Methods and reported in the Results. There was no evidence of selective reporting among multiple outcome measurements.<br><br>No information was available to determine whether multiple eligible statistical analyses were conducted before selecting the reported results.

**Overall bias**	**Risk of bias judgement**			**Some concerns**	
**Ref or Label**	Campbell et al. 2017 [28]	**Aim**	assignment to intervention (the 'intention-to-treat' effect)		
**Experimental**	Treatment	**Comparator**	Control	**Source**	Journal article(s)
**Outcome**	orofacial pain reduction	**Results**	Mean Difference = -1.10	**Weight**	1

**Domain**	**Signalling question**	**Response**	**Comments**
**Bias arising from the randomization process**	1.1 Was the allocation sequence random?	Y	Treatments were assigned using a random-number generator.<br><br>Syringes were labelled with study protocol numbers, and the pharmacist did not break the allocation code until final analysis.
	1.2 Was the allocation sequence concealed until participants were enrolled and assigned to interventions?	Y	
	1.3 Did baseline differences between intervention groups suggest a problem with the randomization process?	N	Baseline demographic characteristics did not show important differences between groups.
	**Risk of bias judgement**	**Low**	Treatments were assigned using a random-number generator.<br><br>Syringes were labelled with study protocol numbers, and the pharmacist did not break the allocation code until final analysis.<br><br>Baseline demographic characteristics did not show important differences between groups.

**Bias due to deviations from intended interventions**	2.1.Were participants aware of their assigned intervention during the trial?	PY	Although the study was double-blind, the intense burning/pain caused by 8% capsaicin may have allowed some participants and investigator to infer their allocation.
	2.2.Were carers and people delivering the interventions aware of participants' assigned intervention during the trial?	PN	
	2.3. If Y/PY/NI to 2.1 or 2.2: Were there deviations from the intended intervention that arose because of the experimental context?	N	No deviations from the intended intervention due to the trial context were reported. Early cream removal occurred because of excessive pain, not because of trial conduct or unblinding.
	2.4 If Y/PY to 2.3: Were these deviations likely to have affected the outcome?	NA	
	2.5. If Y/PY/NI to 2.4: Were these deviations from intended intervention balanced between groups?	NA	
	2.6 Was an appropriate analysis used to estimate the effect of assignment to intervention?	N	One randomized participant in the TMD capsaicin group who discontinued the intervention because of excessive pain was excluded from the analysis. Therefore, the analysis was not performed according to the intention-to-treat principle and did not estimate the effect of assignment to intervention.
	2.7 If N/PN/NI to 2.6: Was there potential for a substantial impact (on the result) of the failure to analyse participants in the group to which they were randomized?	PY	The exclusion of one randomized participant from the capsaicin group may have had a meaningful impact on the estimated treatment effect because of the small sample size and because discontinuation was directly related to the intervention.
	**Risk of bias judgement**	**Some concerns**	
**Bias due to missing outcome data**	3.1 Were data for this outcome available for all, or nearly all, participants randomized?	PY	Outcome data were available for nearly all TMD participants. One TMD participant in the capsaicin group requested early cream removal due to excessive pain and was excluded.
	3.2 If N/PN/NI to 3.1: Is there evidence that result was not biased by missing outcome data?	NA	
	3.3 If N/PN to 3.2: Could missingness in the outcome depend on its true value?	NA	
	3.4 If Y/PY/NI to 3.3: Is it likely that missingness in the outcome depended on its true value?	NA	

	**Risk of bias judgement**	**Low**	Outcome data were available for nearly all TMD participants. One TMD participant in the capsaicin group requested early cream removal due to excessive pain and was excluded.
**Bias in measurement of the outcome**	4.1 Was the method of measuring the outcome inappropriate?	N	Pain was measured using a VAS, which is appropriate for pain assessment.
	4.2 Could measurement or ascertainment of the outcome have differed between intervention groups?	N	The same outcome measurement procedures were used for both groups.
	4.3 Were outcome assessors aware of the intervention received by study participants?	PY	For self-reported pain, participants were the outcome assessors, and some may have inferred allocation due to capsaicin-induced burning.
	4.4 If Y/PY/NI to 4.3: Could assessment of the outcome have been influenced by knowledge of intervention received?	PN	Although participants may have suspected allocation, burning is an expected pharmacological effect and does not necessarily indicate therapeutic benefit; it may even increase pain.
	4.5 If Y/PY/NI to 4.4: Is it likely that assessment of the outcome was influenced by knowledge of intervention received?	NA	
	**Risk of bias judgement**	**Low**	Pain was measured using a VAS, which is appropriate for pain assessment.<br><br>The same outcome measurement procedures were used for both groups.<br><br>For self-reported pain, participants were the outcome assessors, and some may have inferred allocation due to capsaicin-induced burning.<br><br>Although participants may have suspected allocation, burning is an expected pharmacological effect and does not necessarily indicate therapeutic benefit; it may even increase pain.
	5.1 Were the data that produced this result analysed in accordance with a pre-specified analysis plan that	N	No published protocol, trial registration, or pre-specified statistical analysis plan was available.

Bias in selection of the reported result	was finalized before unblinded outcome data were available for analysis?		
	5.2 ... multiple eligible outcome measurements (e.g. scales, definitions, time points) within the outcome domain?	PN	Pain outcomes and time points were described and reported, with no clear evidence that the result was selected from multiple outcome measurements.
	5.3 ... multiple eligible analyses of the data?	NI	It is unclear whether multiple eligible analyses were conducted before selecting the reported analysis.
	**Risk of bias judgement**	**Some concerns**	No published protocol, trial registration, or pre-specified statistical analysis plan was available.<br><br>Pain outcomes and time points were described and reported, with no clear evidence that the result was selected from multiple outcome measurements.<br><br>It is unclear whether multiple eligible analyses were conducted before selecting the reported analysis.
**Overall bias**	**Risk of bias judgement**	**Some concerns**	
