# Supplementary material for: Capsaicin for Orofacial Pain Management: Systematic Review and Meta-Analysis
Source: Int J Mol Sci. 2026 Aug 7;27(16):7099. doi: 10.3390/ijms27167099 (PMC13513309; doi:10.3390/ijms27167099)
Supplement: Supplementary file 1 [file ijms-27-07099-s001.zip › Supplementary File S4.pdf]

SUPPLEMENTARY FILES

Supplementary Table S4. Risk of bias of the included randomized controlled trials, including the crossover trial, assessed using the Cochrane RoB 2 tool.

The Risk Of Bias In Non-randomized Studies – of Interventions (ROBINS-I) assessment tool

(version for cohort-type studies)

Version 19 September 2016

This work is licensed under a Creative Commons Attribution-NonCommercial-NoDerivatives 4.0 International License.

ROBINS-I tool (Stage I): At protocol stage

Specify the review question

Participants	Participants with orofacial pain
Experimental intervention	capsaicin
Comparator	placebo or other pharmacological intervention
Outcomes	managing orofacial pain

List the confounding domains relevant to all or most studies

Baseline pain intensity and severity, age, sex, type of orofacial pain, chronicity of pain, previous or concomitant pain treatment, cancer treatment (when applicable), comorbidities affecting pain perception.

List co-interventions that could be different between intervention groups and that could impact on outcomes

Systemic analgesics (opioids, NSAIDs, acetaminophen), Topical analgesics or anesthetics (e.g., lidocaine), Neuropathic pain medications (e.g., anticonvulsants, antidepressants, clonazepam), Standard supportive care for the underlying condition, Physical or behavioral therapies, Changes in ongoing cancer therapy (when applicable).

ROBINS-I tool (Stage II): For each study

Study: Berger et al. (1995) [33]

Specify a target randomized trial specific to the study

Design	Individually randomized
Participants	Adult patients with oral mucositis pain secondary to chemotherapy and/or radiotherapy for cancer.
Experimental intervention	Oral capsaicin administered in a candy (taffy) formulation.
Comparator	No comparator but the ideal would be Placebo candy (or usual supportive care without capsaicin)

Is your aim for this study…?

☐ to assess the effect of *assignment to* intervention

x to assess the effect of *starting and adhering to* intervention

Specify the outcome

Specify which outcome is being assessed for risk of bias (typically from among those earmarked for the Summary of Findings table). Specify whether this is a proposed benefit or harm of intervention.

Reduction in oral mucositis pain intensity after capsaicin treatment (proposed benefit of the intervention).

Specify the numerical result being assessed

In case of multiple alternative analyses being presented, specify the numeric result (e.g. RR = 1.52 (95% CI 0.83 to 2.77) and/or a reference (e.g. to a table, figure or paragraph) that uniquely defines the result being assessed.

The pain ratings before and after the first capsaicin candy were significantly different (t-- 8.5, P < 0.0001).

Preliminary consideration of confounders

Complete a row for each important confounding domain (i) listed in the review protocol; and (ii) relevant to the setting of this particular study, or which the study authors identified as potentially important.

"Important" confounding domains are those for which, in the context of this study, adjustment is expected to lead to a clinically important change in the estimated effect of the intervention. "Validity" refers to whether the confounding variable or variables fully measure the domain, while "reliability" refers to the precision of the measurement (more measurement error means less reliability).

**(i) Confounding domains listed in the review protocol**				
Confounding domain	Measured variable(s)	Is there evidence that controlling for this variable was unnecessary?*	Is the confounding domain measured validly and reliably by this variable (or these variables)?	OPTIONAL: Is failure to adjust for this variable (alone) expected to favour the experimental intervention or the comparator?
Baseline severity of mucositis	**ECOG mucositis grade**	No	Yes	Favour experimental
Baseline pain intensity	**Pain score (0–10) before treatment**	No	Yes	Favour experimental
Cancer type	Primary cancer site	No	Yes	No information
Cancer treatment	Chemotherapy and/or radiotherapy	No information	No information	No information
Concomitant analgesic therapy	Not adequately reported	No	No information	Favour experimental
Natural progression of mucositis	Not mensured	No	No information	Favour experimental
Placebo effect	Not measured	No	No information	Favour experimental
				Favour experimental / Favour comparator / No information

**(ii) Additional confounding domains relevant to the setting of this particular study, or which the study authors identified as important**				
Confounding domain	Measured variable(s)	Is there evidence that controlling for this variable was unnecessary?*	Is the confounding domain measured validly and reliably by this variable (or these variables)?	OPTIONAL: Is failure to adjust for this variable (alone) expected to favour the experimental intervention or the comparator?
None identified			Yes / No / No information	Favour experimental / Favour comparator / No information

* In the context of a particular study, variables can be demonstrated not to be confounders and so not included in the analysis: (a) if they are not predictive of the outcome; (b) if they are not predictive of intervention; or (c) because adjustment makes no or minimal difference to the estimated effect of the primary parameter. Note that "no statistically significant association" is not the same as "not predictive".

Preliminary consideration of co-interventions

Complete a row for each important co-intervention (i) listed in the review protocol; and (ii) relevant to the setting of this particular study, or which the study authors identified as important.

"Important" co-interventions are those for which, in the context of this study, adjustment is expected to lead to a clinically important change in the estimated effect of the intervention.

**(i) Co-interventions listed in the review protocol**		
Co-intervention	Is there evidence that controlling for this co-intervention was unnecessary (e.g. because it was not administered)?	Is presence of this co-intervention likely to favour outcomes in the experimental intervention or the comparator
Systemic analgesics (opioids/non-opioids/ anesthetics)		Favour experimental
Topical oral analgesics/anesthetics		Favour experimental

**(ii) Additional co-interventions relevant to the setting of this particular study, or which the study authors identified as important**		
Co-intervention	Is there evidence that controlling for this co-intervention was unnecessary (e.g. because it was not administered)?	Is presence of this co-intervention likely to favour outcomes in the experimental intervention or the comparator
None identified		Favour experimental / Favour comparator / No information
		Favour experimental / Favour comparator / No information
		Favour experimental / Favour comparator / No information
		Favour experimental / Favour comparator / No information

Risk of bias assessment

Responses underlined in green are potential markers for low risk of bias, and responses in red are potential markers for a risk of bias. Where questions relate only to sign posts to other questions, no formatting is used.

Signalling questions	Description	Response options
**Bias due to confounding**		
1.1 Is there potential for confounding of the effect of intervention in this study?<br>**If N/PN to 1.1:** the study can be considered to be at low risk of bias due to confounding and no further signalling questions need be considered	The study was an uncontrolled case series without a comparator group. Important confounding factors, including mucositis severity, concomitant analgesic therapy, cancer treatment, and the natural course of mucositis, were not controlled for.	Y
**If Y/PY to 1.1**: determine whether there is a need to assess time-varying confounding:		Y
1.2. Was the analysis based on splitting participants' follow up time according to intervention received?<br>**If N/PN**, answer questions relating to baseline confounding (1.4 to 1.6)<br>**If Y/PY**, go to question 1.3.	Participants received a single intervention and were evaluated immediately before and after treatment. There was no change in intervention status during follow-up.	N
1.3. Were intervention discontinuations or switches likely to be related to factors that are prognostic for the outcome?<br>**If N/PN**, answer questions relating to baseline confounding (1.4 to 1.6)<br>**If Y/PY**, answer questions relating to both baseline and time-varying confounding (1.7 and 1.8)	No treatment switching or discontinuation occurred because the intervention consisted of a single administration. Although two participants informed pain in the full-strength candy use, because that the authors did a half-strength candy for this two.	NA

**Questions relating to baseline confounding only**		
1.4. Did the authors use an appropriate analysis method that controlled for all the important confounding domains?	Only pre- versus post-treatment comparisons were performed. No statistical adjustment for confounding variables was undertaken.	N
1.5. **If Y/PY to 1.4**: Were confounding domains that were controlled for measured validly and reliably by the variables available in this study?		NA / Y / PY / PN / N / NI
1.6. Did the authors control for any post-intervention variables that could have been affected by the intervention?	No post-intervention variables were included in the statistical analysis.	N
**Questions relating to baseline and time-varying confounding**		
1.7. Did the authors use an appropriate analysis method that controlled for all the important confounding domains and for time-varying confounding?	Time-varying confounding was not applicable because the intervention was administered once.	NA
1.8. **If Y/PY to 1.7**: Were confounding domains that were controlled for measured validly and reliably by the variables available in this study?		NA / Y / PY / PN / N / NI
**Risk of bias judgement**	The absence of a control group and lack of adjustment for important confounders substantially increase the risk of biased treatment effect estimates.	**Serious**
Optional: What is the predicted direction of bias due to confounding?		Favours experimental

Bias in selection of participants into the study		
2.1. Was selection of participants into the study (or into the analysis) based on participant characteristics observed after the start of intervention?<br>**If N/PN to 2.1:** go to 2.4	Participants were recruited before receiving capsaicin and were selected based on the presence of oral mucositis pain rather than post-intervention characteristics.	PN
2.2. **If Y/PY to 2.1**: Were the post-intervention variables that influenced selection likely to be associated with intervention?		NA
2.3 **If Y/PY to 2.2**: Were the post-intervention variables that influenced selection likely to be influenced by the outcome or a cause of the outcome?		NA
2.4. Do start of follow-up and start of intervention coincide for most participants?	Baseline pain assessment was performed immediately before capsaicin administration, and follow-up began at intervention initiation.	Y
2.5. **If Y/PY to 2.2 and 2.3, or N/PN to 2.4**: Were adjustment techniques used that are likely to correct for the presence of selection biases?	Adjustment techniques were not required because selection after intervention initiation was unlikely.	NA
**Risk of bias judgement**	Although participants entered the study before treatment, convenience sampling and the absence of a comparison group reduce external validity and increase selection bias.	**Moderate**
Optional: What is the predicted direction of bias due to selection of participants into the study?		Favours experimental

**Bias in classification of interventions**		
3.1 Were intervention groups clearly defined?	The intervention was clearly described as oral capsaicin administered in a candy formulation.	Y
3.2 Was the information used to define intervention groups recorded at the start of the intervention?	Exposure status was recorded before outcome assessment.	Y
3.3 Could classification of intervention status have been affected by knowledge of the outcome or risk of the outcome?	Intervention classification occurred before pain outcomes were measured and therefore could not have been influenced by outcome knowledge.	N
**Risk of bias judgement**	Intervention classification was objective and unlikely to be misclassified.	**Low**
Optional: What is the predicted direction of bias due to classification of interventions?		Favours experimental / Favours comparator / Towards null /Away from null / Unpredictable

Bias due to deviations from intended interventions		
**If your aim for this study is to assess the effect of assignment to intervention, answer questions 4.1 and 4.2**		
4.1. Were there deviations from the intended intervention beyond what would be expected in usual practice?		NA
4.2. **If Y/PY to 4.1**: Were these deviations from intended intervention unbalanced between groups *and* likely to have affected the outcome?		NA
**If your aim for this study is to assess the effect of starting and adhering to intervention, answer questions 4.3 to 4.6**		
4.3. Were important co-interventions balanced across intervention groups?	Concomitant analgesics and supportive treatments were insufficiently reported, and no comparison group was available for assessing balance.	NI
4.4. Was the intervention implemented successfully for most participants?	Most participants completed the intervention, although some received a reduced-strength candy due to intolerance.	PY
4.5. Did study participants adhere to the assigned intervention regimen?	Participants generally completed the intervention as intended, but adherence was not formally assessed.	PY
4.6. **If N/PN to 4.3, 4.4 or 4.5**: Was an appropriate analysis used to estimate the effect of starting and adhering to the intervention?		NA
**Risk of bias judgement**	Incomplete reporting of adherence and co-interventions introduces uncertainty regarding the estimated intervention effect.	**Moderate**
Optional: What is the predicted direction of bias due to deviations from the intended interventions?		Favours experimental

Bias due to missing data		
5.1 Were outcome data available for all, or nearly all, participants?	Outcome data were reported for all participants.	Y
5.2 Were participants excluded due to missing data on intervention status?	No exclusions due to missing intervention data were reported.	N
5.3 Were participants excluded due to missing data on other variables needed for the analysis?	No exclusions based on missing covariate data were reported.	N
5.4 **If PN/N to 5.1, or Y/PY to 5.2 or 5.3**: Are the proportion of participants and reasons for missing data similar across interventions?		NA
5.5 **If PN/N to 5.1, or Y/PY to 5.2 or 5.3**: Is there evidence that results were robust to the presence of missing data?		NA
**Risk of bias judgement**	Outcome completeness appears acceptable.	**Low**
Optional: What is the predicted direction of bias due to missing data?		Favours experimental / Favours comparator / Towards null /Away from null / Unpredictable

Bias in measurement of outcomes		
6.1 Could the outcome measure have been influenced by knowledge of the intervention received?	Pain intensity was a subjective, patient-reported outcome and participants were aware that they had received capsaicin.	Y
6.2 Were outcome assessors aware of the intervention received by study participants?	Neither participants nor investigators were blinded.	Y
6.3 Were the methods of outcome assessment comparable across intervention groups?	No, only one intervention group was included.	N
6.4 Were any systematic errors in measurement of the outcome related to intervention received?	Knowledge of treatment may have influenced patient-reported pain scores through expectation effects.	PY
**Risk of bias judgement**	The primary outcome was subjective and measured without blinding, making it highly susceptible to measurement bias.	**Serious**
Optional: What is the predicted direction of bias due to measurement of outcomes?		Favours experimental

**Bias in selection of the reported result**		
Is the reported effect estimate likely to be selected, on the basis of the results, from...		
7.1. ... multiple outcome *measurements* within the outcome domain?	The study assessed pain before and after capsaicin using a subjective pain score, but the protocol was not available and it is unclear whether other pain time points, formulations, or pain-related measures were assessed but not fully reported.	PY
7.2 ... multiple *analyses* of the intervention-outcome relationship?	The main analysis appears to be a simple before–after comparison of pain scores. There is no clear evidence that multiple statistical models or alternative analyses were conducted and selectively reported.	PN
7.3 ... different *subgroups*?	Although full-strength and half-strength candies were described, the main result was not clearly selected from multiple subgroup analyses. There is no evidence that subgroup-specific results were selectively emphasized based on statistical significance.	PN
**Risk of bias judgement**		**Moderate**
Optional: What is the predicted direction of bias due to selection of the reported result?		Favours experimental

**Overall bias**		
**Risk of bias judgement**		**Serious**
Optional: What is the overall predicted direction of bias for this outcome?	The study was an uncontrolled pilot case series without randomization, comparator group, or blinding. Pain was measured using a subjective self-reported scale immediately before and after treatment, making the results susceptible to confounding, placebo effects, and measurement bias. Although intervention classification was clear, the absence of control for major confounding factors and the subjective outcome assessment resulted in an overall serious risk of bias.	Favours experimental

ROBINS-I tool (Stage II): For each study

Study: Fusco et al. (1992)[29]

Specify a target randomized trial specific to the study

Design	Individually randomized
Participants	See original assessment prepared in chat.
Experimental intervention	Capsaicin intervention evaluated in the study.
Comparator	Placebo/usual care (target trial comparator).

Is your aim for this study…?

☐ to assess the effect of *assignment to* intervention

x to assess the effect of *starting and adhering to* intervention

Specify the outcome

Specify which outcome is being assessed for risk of bias (typically from among those earmarked for the Summary of Findings table). Specify whether this is a proposed benefit or harm of intervention.

Reduction in pain intensity associated with idiopathic trigeminal neuralgia (beneficial outcome).

Specify the numerical result being assessed

In case of multiple alternative analyses being presented, specify the numeric result (e.g. RR = 1.52 (95% CI 0.83 to 2.77) and/or a reference (e.g. to a table, figure or paragraph) that uniquely defines the result being assessed.

Preliminary consideration of confounders

Complete a row for each important confounding domain (i) listed in the review protocol; and (ii) relevant to the setting of this particular study, or which the study authors identified as potentially important.

"Important" confounding domains are those for which, in the context of this study, adjustment is expected to lead to a clinically important change in the estimated effect of the intervention. "Validity" refers to whether the confounding variable or variables fully measure the domain, while "reliability" refers to the precision of the measurement (more measurement error means less reliability).

**(i) Confounding domains listed in the review protocol**				
Confounding domain	Measured variable(s)	Is there evidence that controlling for this variable was unnecessary?*	Is the confounding domain measured validly and reliably by this variable (or these variables)?	OPTIONAL: Is failure to adjust for this variable (alone) expected to favour the experimental intervention or the comparator?
Baseline pain intensity	Pain severity score	No	Yes	Favour experimental
Baseline disease severity	Duration and clinical characteristics of trigeminal neuralgia	No	Probably Yes	Favour experimental
Duration of symptoms	Years since diagnosis	No	Yes	Favour experimental
Previous pain treatment	Previous carbamazepine/phenytoin use	No	Yes	Favour experimental
Age	Years	No	Yes	No information
Sex	Male/Female	No	Yes	No information
Comorbidities	Not reported	No information	No information	No information
				No information

**(ii) Additional confounding domains relevant to the setting of this particular study, or which the study authors identified as important**				
Confounding domain	Measured variable(s)	Is there evidence that controlling for this variable was unnecessary?*	Is the confounding domain measured validly and reliably by this variable (or these variables)?	OPTIONAL: Is failure to adjust for this variable (alone) expected to favour the experimental intervention or the comparator?
None identified			Yes / No / No information	Favour experimental / Favour comparator / No information

* In the context of a particular study, variables can be demonstrated not to be confounders and so not included in the analysis: (a) if they are not predictive of the outcome; (b) if they are not predictive of intervention; or (c) because adjustment makes no or minimal difference to the estimated effect of the primary parameter. Note that "no statistically significant association" is not the same as "not predictive".

Preliminary consideration of co-interventions

Complete a row for each important co-intervention (i) listed in the review protocol; and (ii) relevant to the setting of this particular study, or which the study authors identified as important.

"Important" co-interventions are those for which, in the context of this study, adjustment is expected to lead to a clinically important change in the estimated effect of the intervention.

**(i) Co-interventions listed in the review protocol**		
Co-intervention	Is there evidence that controlling for this co-intervention was unnecessary (e.g. because it was not administered)?	Is presence of this co-intervention likely to favour outcomes in the experimental intervention or the comparator
Systemic analgesics	Yes (previous treatments were discontinued ≥10 days before capsaicin)	No information
Neuropathic pain medications	Yes	No information
Topical analgesics	No information	No information
Other pain therapies	No information	No information

**(ii) Additional co-interventions relevant to the setting of this particular study, or which the study authors identified as important**		
Co-intervention	Is there evidence that controlling for this co-intervention was unnecessary (e.g. because it was not administered)?	Is presence of this co-intervention likely to favour outcomes in the experimental intervention or the comparator
None identified.		Favour experimental / Favour comparator / No information
		Favour experimental / Favour comparator / No information
		Favour experimental / Favour comparator / No information
		Favour experimental / Favour comparator / No information

Risk of bias assessment

Responses underlined in green are potential markers for low risk of bias, and responses in red are potential markers for a risk of bias. Where questions relate only to sign posts to other questions, no formatting is used.

Signalling questions	Description	Response options
**Bias due to confounding**		
1.1 Is there potential for confounding of the effect of intervention in this study?<br>**If N/PN to 1.1:** the study can be considered to be at low risk of bias due to confounding and no further signalling questions need be considered	The study lacked a control group and randomization. Baseline pain severity, disease duration, placebo effects, and spontaneous fluctuation of trigeminal neuralgia could influence the observed treatment effect.	Y
**If Y/PY to 1.1**: determine whether there is a need to assess time-varying confounding:		
1.2. Was the analysis based on splitting participants' follow up time according to intervention received?<br>**If N/PN**, answer questions relating to baseline confounding (1.4 to 1.6)<br>**If Y/PY**, go to question 1.3.	All participants received the same intervention throughout follow-up.	N
1.3. Were intervention discontinuations or switches likely to be related to factors that are prognostic for the outcome?<br>**If N/PN**, answer questions relating to baseline confounding (1.4 to 1.6)<br>**If Y/PY**, answer questions relating to both baseline and time-varying confounding (1.7 and 1.8)	No treatment switching occurred. Patients who relapsed underwent a second treatment cycle.	NA

**Questions relating to baseline confounding only**		
1.4. Did the authors use an appropriate analysis method that controlled for all the important confounding domains?	No statistical adjustment for confounding variables was performed.	N
1.5. **If Y/PY to 1.4**: Were confounding domains that were controlled for measured validly and reliably by the variables available in this study?		NA
1.6. Did the authors control for any post-intervention variables that could have been affected by the intervention?	No post-intervention variables were included in the analysis.	N
**Questions relating to baseline and time-varying confounding**		Not applicable.
1.7. Did the authors use an appropriate analysis method that controlled for all the important confounding domains and for time-varying confounding?	The study did not involve changes in intervention status over time, treatment switching, or time-varying exposure. All participants received the same topical capsaicin regimen throughout the study, and no analytical methods addressing time-varying confounding were required.	NA
1.8. **If Y/PY to 1.7**: Were confounding domains that were controlled for measured validly and reliably by the variables available in this study?		NA
**Risk of bias judgement**		**Serious**
Optional: What is the predicted direction of bias due to confounding?		Favours experimental

Bias in selection of participants into the study		
2.1. Was selection of participants into the study (or into the analysis) based on participant characteristics observed after the start of intervention?<br>**If N/PN to 2.1:** go to 2.4	Participants were enrolled before treatment based on clinical diagnosis of idiopathic trigeminal neuralgia.	PN
2.2. **If Y/PY to 2.1**: Were the post-intervention variables that influenced selection likely to be associated with intervention?		NA
2.3 **If Y/PY to 2.2**: Were the post-intervention variables that influenced selection likely to be influenced by the outcome or a cause of the outcome?		NA
2.4. Do start of follow-up and start of intervention coincide for most participants?	Follow-up started when topical capsaicin treatment began.	Y
2.5. **If Y/PY to 2.2 and 2.3, or N/PN to 2.4**: Were adjustment techniques used that are likely to correct for the presence of selection biases?		NA
**Risk of bias judgement**		Moderate
Optional: What is the predicted direction of bias due to selection of participants into the study?		Unpredictable

Bias in classification of interventions		
3.1 Were intervention groups clearly defined?	Capsaicin concentration (0.05%), dose, frequency, and treatment area were clearly described.	Y
3.2 Was the information used to define intervention groups recorded at the start of the intervention?	Treatment assignment preceded outcome measurement.	Y
3.3 Could classification of intervention status have been affected by knowledge of the outcome or risk of the outcome?	Intervention status could not have been influenced by pain outcomes.	N
**Risk of bias judgement**		Low
Optional: What is the predicted direction of bias due to classification of interventions?		Favours experimental / Favours comparator / Towards null /Away from null / Unpredictable

**Bias due to deviations from intended interventions**		
**If your aim for this study is to assess the effect of assignment to intervention, answer questions 4.1 and 4.2**		
4.1. Were there deviations from the intended intervention beyond what would be expected in usual practice?		NA
4.2. **If Y/PY to 4.1**: Were these deviations from intended intervention unbalanced between groups *and* likely to have affected the outcome?		NA
**If your aim for this study is to assess the effect of starting and adhering to intervention, answer questions 4.3 to 4.6**		
4.3. Were important co-interventions balanced across intervention groups?	Previous medications were discontinued before treatment, reducing imbalance, although complete information on concomitant therapies during follow-up was unavailable.	PY
4.4. Was the intervention implemented successfully for most participants?	Patients completed the prescribed topical capsaicin regimen.	Y
4.5. Did study participants adhere to the assigned intervention regimen?	Treatment was self-administered three times daily, but adherence was not objectively monitored.	PY
4.6. **If N/PN to 4.3, 4.4 or 4.5**: Was an appropriate analysis used to estimate the effect of starting and adhering to the intervention?		
**Risk of bias judgement**		**Moderate**
Optional: What is the predicted direction of bias due to deviations from the intended interventions?		Favours experimental

Bias due to missing data		
5.1 Were outcome data available for all, or nearly all, participants?	Outcomes were reported for all 12 enrolled participants.	Y
5.2 Were participants excluded due to missing data on intervention status?	No missing intervention information was reported.	N
5.3 Were participants excluded due to missing data on other variables needed for the analysis?	No exclusions due to missing variables were reported.	N
5.4 **If PN/N to 5.1, or Y/PY to 5.2 or 5.3**: Are the proportion of participants and reasons for missing data similar across interventions?		NA
5.5 **If PN/N to 5.1, or Y/PY to 5.2 or 5.3**: Is there evidence that results were robust to the presence of missing data?		NA
**Risk of bias judgement**		**Low**
Optional: What is the predicted direction of bias due to missing data?		Favours experimental / Favours comparator / Towards null /Away from null / Unpredictable

Bias in measurement of outcomes		
6.1 Could the outcome measure have been influenced by knowledge of the intervention received?	Pain relief was self-reported in an unblinded study, making placebo and expectation effects likely.	Y
6.2 Were outcome assessors aware of the intervention received by study participants?	Neither participants nor investigators were blinded.	Y
6.3 Were the methods of outcome assessment comparable across intervention groups?	Only one intervention group was evaluated.	N
6.4 Were any systematic errors in measurement of the outcome related to intervention received?	Subjective pain assessment without blinding increases the likelihood of measurement bias.	PY
**Risk of bias judgement**		**Serious**
Optional: What is the predicted direction of bias due to measurement of outcomes?		Favours experimental

Bias in selection of the reported result		
Is the reported effect estimate likely to be selected, on the basis of the results, from...		
7.1. ... multiple outcome *measurements* within the outcome domain?	No protocol was available, making it unclear whether additional pain outcomes were measured but not reported.	PY
7.2 ... multiple *analyses* of the intervention-outcome relationship?	Only descriptive analyses of pain response were reported, with no indication of multiple statistical models.	PN
7.3 ... different *subgroups*?	No subgroup analyses were reported.	PN
**Risk of bias judgement**		**Moderate**
Optional: What is the predicted direction of bias due to selection of the reported result?		Favours experimental

Overall bias		
**Risk of bias judgement**		**Serious**
Optional: What is the overall predicted direction of bias for this outcome?		Favours experimental

ROBINS-I tool (Stage II): For each study

Study: Wagner et al. (2013)

Specify a target randomized trial specific to the study

Design	Individually randomized
Participants	Adult patients with peripheral neuropathic pain (including postherpetic neuralgia, facial neuropathy/trigeminal neuralgia, polyneuropathy, radiculopathy, post-surgical neuropathy, and other peripheral neuropathic pain conditions).
Experimental intervention	Capsaicin 8% patch (Qutenza®) applied according to the manufacturer's protocol.
Comparator	Placebo patch/or standard care alone.

Is your aim for this study…?

☐ to assess the effect of *assignment to* intervention

x to assess the effect of *starting and adhering to* intervention

Specify the outcome

Specify which outcome is being assessed for risk of bias (typically from among those earmarked for the Summary of Findings table). Specify whether this is a proposed benefit or harm of intervention.

Reduction in pain intensity measured using the Numeric Pain Rating Scale (NPRS) (beneficial outcome).

Specify the numerical result being assessed

In case of multiple alternative analyses being presented, specify the numeric result (e.g. RR = 1.52 (95% CI 0.83 to 2.77) and/or a reference (e.g. to a table, figure or paragraph) that uniquely defines the result being assessed.

Preliminary consideration of confounders

Complete a row for each important confounding domain (i) listed in the review protocol; and (ii) relevant to the setting of this particular study, or which the study authors identified as potentially important.

"Important" confounding domains are those for which, in the context of this study, adjustment is expected to lead to a clinically important change in the estimated effect of the intervention. "Validity" refers to whether the confounding variable or variables fully measure the domain, while "reliability" refers to the precision of the measurement (more measurement error means less reliability).

**(i) Confounding domains listed in the review protocol**				
Confounding domain	Measured variable(s)	Is there evidence that controlling for this variable was unnecessary?*	Is the confounding domain measured validly and reliably by this variable (or these variables)?	OPTIONAL: Is failure to adjust for this variable (alone) expected to favour the experimental intervention or the comparator?
Baseline pain intensity	NPRS score	No	Yes	Favour experimental
Baseline severity of neuropathic pain	Clinical diagnosis and painful area	No	Probably Yes	Favour experimental
Duration of symptoms	Months since diagnosis	No	Yes	Favour experimental
Underlying diagnosis	Type of neuropathic pain	No	Yes	Favour experimental
Previous/concomitant pain treatment	Neuropathic pain medications	No	Yes	Favour experimental
Age	Years	No	Yes	No information
Sex	Male/Female	No	Yes	No information
Comorbidities	Reported clinically	No	Probably Yes	No information

(ii) Additional confounding domains relevant to the setting of this particular study, or which the study authors identified as important				
Confounding domain	Measured variable(s)	Is there evidence that controlling for this variable was unnecessary?*	Is the confounding domain measured validly and reliably by this variable (or these variables)?	OPTIONAL: Is failure to adjust for this variable (alone) expected to favour the experimental intervention or the comparator?
None identified.			Yes / No / No information	Favour experimental / Favour comparator / No information

* In the context of a particular study, variables can be demonstrated not to be confounders and so not included in the analysis: (a) if they are not predictive of the outcome; (b) if they are not predictive of intervention; or (c) because adjustment makes no or minimal difference to the estimated effect of the primary parameter. Note that "no statistically significant association" is not the same as "not predictive".

Preliminary consideration of co-interventions

Complete a row for each important co-intervention (i) listed in the review protocol; and (ii) relevant to the setting of this particular study, or which the study authors identified as important.

"Important" co-interventions are those for which, in the context of this study, adjustment is expected to lead to a clinically important change in the estimated effect of the intervention.

**(i) Co-interventions listed in the review protocol**		
Co-intervention	Is there evidence that controlling for this co-intervention was unnecessary (e.g. because it was not administered)?	Is presence of this co-intervention likely to favour outcomes in the experimental intervention or the comparator
Systemic analgesics	No	Favour experimental
Neuropathic pain medications	No	Favour experimental
Topical analgesics	No information	No information
Standard supportive care	No information	No information

**(ii) Additional co-interventions relevant to the setting of this particular study, or which the study authors identified as important**		
Co-intervention	Is there evidence that controlling for this co-intervention was unnecessary (e.g. because it was not administered)?	Is presence of this co-intervention likely to favour outcomes in the experimental intervention or the comparator
Topical anesthetic (EMLA®) before patch application	Yes	No information
		Favour experimental / Favour comparator / No information
		Favour experimental / Favour comparator / No information
		Favour experimental / Favour comparator / No information

Risk of bias assessment

Responses underlined in green are potential markers for low risk of bias, and responses in red are potential markers for a risk of bias. Where questions relate only to sign posts to other questions, no formatting is used.

Signalling questions	Description	Response options
**Bias due to confounding**		
1.1 Is there potential for confounding of the effect of intervention in this study?<br>**If N/PN to 1.1:** the study can be considered to be at low risk of bias due to confounding and no further signalling questions need be considered	This retrospective observational study lacked a comparator group and randomization. Baseline disease severity, underlying diagnosis, concomitant medications, and clinical decision-making could have influenced treatment response.	Y
**If Y/PY to 1.1**: determine whether there is a need to assess time-varying confounding:		
1.2. Was the analysis based on splitting participants' follow up time according to intervention received?<br>**If N/PN**, answer questions relating to baseline confounding (1.4 to 1.6)<br>**If Y/PY**, go to question 1.3.	All participants received the capsaicin patch. Follow-up was not divided according to different interventions.	N
1.3. Were intervention discontinuations or switches likely to be related to factors that are prognostic for the outcome?<br>**If N/PN**, answer questions relating to baseline confounding (1.4 to 1.6)<br>**If Y/PY**, answer questions relating to both baseline and time-varying confounding (1.7 and 1.8)	No switching between interventions occurred. Retreatment with capsaicin was part of routine clinical management.	NA

**Questions relating to baseline confounding only**		
1.4. Did the authors use an appropriate analysis method that controlled for all the important confounding domains?	No multivariable statistical adjustment or propensity score methods were performed.	N
1.5. **If Y/PY to 1.4**: Were confounding domains that were controlled for measured validly and reliably by the variables available in this study?	No adjustment for confounding was undertaken.	NA
1.6. Did the authors control for any post-intervention variables that could have been affected by the intervention?	No post-intervention variables were adjusted for.	N
**Questions relating to baseline and time-varying confounding**		
1.7. Did the authors use an appropriate analysis method that controlled for all the important confounding domains and for time-varying confounding?		N
1.8. **If Y/PY to 1.7**: Were confounding domains that were controlled for measured validly and reliably by the variables available in this study?		NA
**Risk of bias judgement**		**Serious**
Optional: What is the predicted direction of bias due to confounding?		Favours experimental

Bias in selection of participants into the study		
2.1. Was selection of participants into the study (or into the analysis) based on participant characteristics observed after the start of intervention?<br>**If N/PN to 2.1:** go to 2.4	Patients were included before treatment based on eligibility for capsaicin patch therapy.	PN
2.2. **If Y/PY to 2.1**: Were the post-intervention variables that influenced selection likely to be associated with intervention?		NA
2.3 **If Y/PY to 2.2**: Were the post-intervention variables that influenced selection likely to be influenced by the outcome or a cause of the outcome?	Not applicable.	NA
2.4. Do start of follow-up and start of intervention coincide for most participants?	Follow-up began when the first capsaicin patch was applied.	Y
2.5. **If Y/PY to 2.2 and 2.3, or N/PN to 2.4**: Were adjustment techniques used that are likely to correct for the presence of selection biases?	Not applicable.	NA
**Risk of bias judgement**		Moderate
Optional: What is the predicted direction of bias due to selection of participants into the study?		Unpredictable

Bias in classification of interventions		
3.1 Were intervention groups clearly defined?	The intervention (capsaicin 8% patch) was clearly described according to the manufacturer's protocol.	Y
3.2 Was the information used to define intervention groups recorded at the start of the intervention?	Treatment exposure was documented before outcome assessment.	Y
3.3 Could classification of intervention status have been affected by knowledge of the outcome or risk of the outcome?	Intervention classification was established before pain outcomes were measured.	N
**Risk of bias judgement**		Low
Optional: What is the predicted direction of bias due to classification of interventions?		Favours experimental / Favours comparator / Towards null /Away from null / Unpredictable

Bias due to deviations from intended interventions		
**If your aim for this study is to assess the effect of assignment to intervention, answer questions 4.1 and 4.2**		
4.1. Were there deviations from the intended intervention beyond what would be expected in usual practice?		NA
4.2. **If Y/PY to 4.1**: Were these deviations from intended intervention unbalanced between groups *and* likely to have affected the outcome?		NA
**If your aim for this study is to assess the effect of starting and adhering to intervention, answer questions 4.3 to 4.6**		
4.3. Were important co-interventions balanced across intervention groups?	Most patients continued concomitant neuropathic pain medications. Although medication use was documented, balance across participants could not be evaluated because there was no comparison group.	PY
4.4. Was the intervention implemented successfully for most participants?	The capsaicin patch was applied according to standard clinical procedures in nearly all patients.	Y
4.5. Did study participants adhere to the assigned intervention regimen?	Patch application was supervised in the clinic, ensuring adherence to the treatment protocol.	Y
4.6. **If N/PN to 4.3, 4.4 or 4.5**: Was an appropriate analysis used to estimate the effect of starting and adhering to the intervention?		NA
**Risk of bias judgement**		**Moderate**
Optional: What is the predicted direction of bias due to deviations from the intended interventions?		Favours experimental

Bias due to missing data		
5.1 Were outcome data available for all, or nearly all, participants?	Only 53 of the 68 treated patients had evaluable 8-week follow-up data, indicating incomplete outcome data.	PN
5.2 Were participants excluded due to missing data on intervention status?	Intervention status was available for all treated patients.	N
5.3 Were participants excluded due to missing data on other variables needed for the analysis?	No exclusions due to missing baseline variables were reported.	N
5.4 **If PN/N to 5.1, or Y/PY to 5.2 or 5.3**: Are the proportion of participants and reasons for missing data similar across interventions?	Reasons for incomplete follow-up were not fully described.	N
5.5 **If PN/N to 5.1, or Y/PY to 5.2 or 5.3**: Is there evidence that results were robust to the presence of missing data?	No sensitivity analyses addressing missing follow-up data were reported.	N
**Risk of bias judgement**		**Moderate**
Optional: What is the predicted direction of bias due to missing data?		Favours experimental

Bias in measurement of outcomes		
6.1 Could the outcome measure have been influenced by knowledge of the intervention received?	Pain relief was self-reported in an unblinded study, making placebo and expectation effects likely.	Y
6.2 Were outcome assessors aware of the intervention received by study participants?	Neither participants nor investigators were blinded.	Y
6.3 Were the methods of outcome assessment comparable across intervention groups?	Only one intervention group was evaluated.	N
6.4 Were any systematic errors in measurement of the outcome related to intervention received?	Subjective pain assessment without blinding increases the likelihood of measurement bias.	PY
**Risk of bias judgement**		**Serious**
Optional: What is the predicted direction of bias due to measurement of outcomes?		Favours experimental

Bias in selection of the reported result		
Is the reported effect estimate likely to be selected, on the basis of the results, from...		
7.1. ... multiple outcome *measurements* within the outcome domain?	No prespecified protocol was available, and several efficacy outcomes (NPRS, responder rate, medication reduction, retreatment interval) were reported, making selective reporting possible.	PY
7.2 ... multiple *analyses* of the intervention-outcome relationship?	The analyses presented were descriptive and consistent with the study objectives, with no evidence of multiple statistical models.	PN
7.3 ... different *subgroups*?	The study included several neuropathic pain conditions, but subgroup analyses were limited and not clearly prespecified, raising some concern regarding selective subgroup reporting.	PY
**Risk of bias judgement**		**Moderate**
Optional: What is the predicted direction of bias due to selection of the reported result?		Favours experimental

Overall bias		
**Risk of bias judgement**		**Moderate**
Optional: What is the overall predicted direction of bias for this outcome?		Favours experimental

ROBINS-I tool (Stage II): For each study

Study: Gonçalves et al. (2020)

Specify a target randomized trial specific to the study

Design	Individually randomized
Participants	Adult patients with peripheral neuropathic pain refractory to first-line therapy, including postherpetic neuralgia, chronic post-surgical pain, post-traumatic neuropathic pain, diabetic neuropathy, CRPS, HIV-associated neuropathy, lumbar neuropathic pain, trigeminal neuralgia, and other neuropathies.
Experimental intervention	Capsaicin 8% patch (Qutenza®).
Comparator	Placebo patch /or standard care alone.

Is your aim for this study…?

- ☐ to assess the effect of *assignment to* intervention
- x to assess the effect of *starting and adhering to* intervention

Specify the outcome

Specify which outcome is being assessed for risk of bias (typically from among those earmarked for the Summary of Findings table). Specify whether this is a proposed benefit or harm of intervention.

Reduction in pain intensity measured using the Numeric Rating Scale (NRS-11) (beneficial outcome).

Specify the numerical result being assessed

In case of multiple alternative analyses being presented, specify the numeric result (e.g. RR = 1.52 (95% CI 0.83 to 2.77) and/or a reference (e.g. to a table, figure or paragraph) that uniquely defines the result being assessed.

The proportion of patients achieving a ≥30% reduction in baseline pain intensity was 40.8% at 15 days, 43.3% at 8 weeks, and 45.0% at 12 weeks. Additionally, 30.0% achieved a ≥50% reduction at 12 weeks. Pain area decreased in 36.7% of patients, and 18.3% reduced concomitant analgesic therapy.

Preliminary consideration of confounders

Complete a row for each important confounding domain (i) listed in the review protocol; and (ii) relevant to the setting of this particular study, or which the study authors identified as potentially important.

"Important" confounding domains are those for which, in the context of this study, adjustment is expected to lead to a clinically important change in the estimated effect of the intervention. "Validity" refers to whether the confounding variable or variables fully measure the domain, while "reliability" refers to the precision of the measurement (more measurement error means less reliability).

**(i) Confounding domains listed in the review protocol**				
Confounding domain	Measured variable(s)	Is there evidence that controlling for this variable was unnecessary?*	Is the confounding domain measured validly and reliably by this variable (or these variables)?	OPTIONAL: Is failure to adjust for this variable (alone) expected to favour the experimental intervention or the comparator?
Duration of symptoms	Not consistently reported	No	No information	Favour experimental
Underlying diagnosis	Etiology of neuropathic pain	No	Yes	Favour experimental
Previous/concomitant pain treatment	Chronic analgesic therapy	No	Yes	Favour experimental
Age	Years	No	Yes	No information
Sex	Male/Female	No	Yes	No information
Comorbidities	Clinical diagnosis	No	Probably Yes	No information

(ii) Additional confounding domains relevant to the setting of this particular study, or which the study authors identified as important				
Confounding domain	Measured variable(s)	Is there evidence that controlling for this variable was unnecessary?*	Is the confounding domain measured validly and reliably by this variable (or these variables)?	OPTIONAL: Is failure to adjust for this variable (alone) expected to favour the experimental intervention or the comparator?
None identified.			Yes / No / No information	Favour experimental / Favour comparator / No information

* In the context of a particular study, variables can be demonstrated not to be confounders and so not included in the analysis: (a) if they are not predictive of the outcome; (b) if they are not predictive of intervention; or (c) because adjustment makes no or minimal difference to the estimated effect of the primary parameter. Note that “no statistically significant association” is not the same as “not predictive”.

Preliminary consideration of co-interventions

Complete a row for each important co-intervention (i) listed in the review protocol; and (ii) relevant to the setting of this particular study, or which the study authors identified as important.

"Important" co-interventions are those for which, in the context of this study, adjustment is expected to lead to a clinically important change in the estimated effect of the intervention.

**(i) Co-interventions listed in the review protocol**		
Co-intervention	Is there evidence that controlling for this co-intervention was unnecessary (e.g. because it was not administered)?	Is presence of this co-intervention likely to favour outcomes in the experimental intervention or the comparator
Systemic analgesics	No	Favour experimental
Neuropathic pain medications	No	Favour experimental
Topical analgesics	No information	No information
Standard supportive care	No information	No information

**(ii) Additional co-interventions relevant to the setting of this particular study, or which the study authors identified as important**		
Co-intervention	Is there evidence that controlling for this co-intervention was unnecessary (e.g. because it was not administered)?	Is presence of this co-intervention likely to favour outcomes in the experimental intervention or the comparator
Oral paracetamol (1 g before patch application)	Yes	No information
		Favour experimental / Favour comparator / No information
		Favour experimental / Favour comparator / No information
		Favour experimental / Favour comparator / No information

Risk of bias assessment

Responses underlined in green are potential markers for low risk of bias, and responses in red are potential markers for a risk of bias. Where questions relate only to sign posts to other questions, no formatting is used.

Signalling questions	Description	Response options
**Bias due to confounding**		
1.1 Is there potential for confounding of the effect of intervention in this study?<br>**If N/PN to 1.1:** the study can be considered to be at low risk of bias due to confounding and no further signalling questions need be considered	The study was retrospective and uncontrolled. Baseline disease severity, neuropathic pain etiology, concomitant medications, and clinician treatment decisions could influence outcomes independently of the capsaicin patch.	Y
**If Y/PY to 1.1**: determine whether there is a need to assess time-varying confounding:		
1.2. Was the analysis based on splitting participants' follow up time according to intervention received?<br>**If N/PN**, answer questions relating to baseline confounding (1.4 to 1.6)<br>**If Y/PY**, go to question 1.3.	All participants received the same intervention and remained in the same exposure group throughout follow-up.	N
1.3. Were intervention discontinuations or switches likely to be related to factors that are prognostic for the outcome?<br>**If N/PN**, answer questions relating to baseline confounding (1.4 to 1.6)<br>**If Y/PY**, answer questions relating to both baseline and time-varying confounding (1.7 and 1.8)	No switching between intervention groups occurred.	NA

**Questions relating to baseline confounding only**		
1.4. Did the authors use an appropriate analysis method that controlled for all the important confounding domains?	No multivariable adjustment, propensity score analysis, or matching procedures were performed.	N
1.5. **If Y/PY to 1.4**: Were confounding domains that were controlled for measured validly and reliably by the variables available in this study?		NA
1.6. Did the authors control for any post-intervention variables that could have been affected by the intervention?	No adjustment for post-intervention variables was reported.	N
**Questions relating to baseline and time-varying confounding**		Not applicable.
1.7. Did the authors use an appropriate analysis method that controlled for all the important confounding domains and for time-varying confounding?	The study did not involve changes in intervention status over time, treatment switching, or time-varying exposure. All participants received the same topical capsaicin regimen throughout the study, and no analytical methods addressing time-varying confounding were required.	N
1.8. **If Y/PY to 1.7**: Were confounding domains that were controlled for measured validly and reliably by the variables available in this study?		NA
**Risk of bias judgement**		**Serious**
Optional: What is the predicted direction of bias due to confounding?		Favours experimental

Bias in selection of participants into the study		
2.1. Was selection of participants into the study (or into the analysis) based on participant characteristics observed after the start of intervention?<br>**If N/PN to 2.1:** go to 2.4	Patients were included before receiving the capsaicin patch according to routine clinical eligibility criteria.	PN
2.2. **If Y/PY to 2.1**: Were the post-intervention variables that influenced selection likely to be associated with intervention?		NA
2.3 **If Y/PY to 2.2**: Were the post-intervention variables that influenced selection likely to be influenced by the outcome or a cause of the outcome?		NA
2.4. Do start of follow-up and start of intervention coincide for most participants?	Baseline assessment was performed immediately before patch application.	Y
2.5. **If Y/PY to 2.2 and 2.3, or N/PN to 2.4**: Were adjustment techniques used that are likely to correct for the presence of selection biases?		NA
**Risk of bias judgement**		Moderate
Optional: What is the predicted direction of bias due to selection of participants into the study?		Unpredictable

Bias in classification of interventions		
3.1 Were intervention groups clearly defined?	The intervention (capsaicin 8% patch) was clearly described, including dose, application time, and treatment protocol.	Y
3.2 Was the information used to define intervention groups recorded at the start of the intervention?	Exposure was documented before follow-up assessments.	Y
3.3 Could classification of intervention status have been affected by knowledge of the outcome or risk of the outcome?	Intervention classification occurred before outcome measurement.	N
**Risk of bias judgement**		Low
Optional: What is the predicted direction of bias due to classification of interventions?		Favours experimental / Favours comparator / Towards null /Away from null / Unpredictable

Bias due to deviations from intended interventions		
**If your aim for this study is to assess the effect of assignment to intervention, answer questions 4.1 and 4.2**		
4.1. Were there deviations from the intended intervention beyond what would be expected in usual practice?		NA
4.2. **If Y/PY to 4.1**: Were these deviations from intended intervention unbalanced between groups *and* likely to have affected the outcome?		NA
**If your aim for this study is to assess the effect of starting and adhering to intervention, answer questions 4.3 to 4.6**		
4.3. Were important co-interventions balanced across intervention groups?	Patients continued individualized analgesic regimens, and medication adjustments occurred during follow-up. Because there was no comparator group, balance could not be assessed.	PY
4.4. Was the intervention implemented successfully for most participants?	The capsaicin patch was applied according to a standardized institutional protocol under medical supervision.	Y
4.5. Did study participants adhere to the assigned intervention regimen?	Patch application was supervised by healthcare professionals, ensuring protocol adherence.	Y
4.6. **If N/PN to 4.3, 4.4 or 4.5**: Was an appropriate analysis used to estimate the effect of starting and adhering to the intervention?		NA
**Risk of bias judgement**		Moderate
Optional: What is the predicted direction of bias due to deviations from the intended interventions?		Favours experimental / Favours comparator / Towards null /Away from null / Unpredictable

Bias due to missing data		
5.1 Were outcome data available for all, or nearly all, participants?	Most patients completed the scheduled follow-up assessments, although the article does not explicitly report losses at every time point.	PY
5.2 Were participants excluded due to missing data on intervention status?	No exclusions due to missing intervention information were reported.	N
5.3 Were participants excluded due to missing data on other variables needed for the analysis?	No exclusions based on missing baseline variables were described.	N
5.4 **If PN/N to 5.1, or Y/PY to 5.2 or 5.3**: Are the proportion of participants and reasons for missing data similar across interventions?		NA
5.5 **If PN/N to 5.1, or Y/PY to 5.2 or 5.3**: Is there evidence that results were robust to the presence of missing data?		NA
**Risk of bias judgement**		**Low**
Optional: What is the predicted direction of bias due to missing data?		Favours experimental / Favours comparator / Towards null /Away from null / Unpredictable

Bias in measurement of outcomes		
6.1 Could the outcome measure have been influenced by knowledge of the intervention received?	Pain intensity was self-reported using the NRS-11, and participants knew they had received active treatment.	Y
6.2 Were outcome assessors aware of the intervention received by study participants?	Neither patients nor clinicians were blinded to treatment.	Y
6.3 Were the methods of outcome assessment comparable across intervention groups?	Only one intervention group was included.	N
6.4 Were any systematic errors in measurement of the outcome related to intervention received?	Expectation effects and the absence of blinding could have influenced subjective pain ratings.	PY
**Risk of bias judgement**		**Serious**
Optional: What is the predicted direction of bias due to measurement of outcomes?		Favours experimental

Bias in selection of the reported result		
Is the reported effect estimate likely to be selected, on the basis of the results, from...		
7.1. ... multiple outcome *measurements* within the outcome domain?	No prespecified protocol was available, and several efficacy outcomes (NPRS, responder rate, medication reduction, retreatment interval) were reported, making selective reporting possible.	PY
7.2 ... multiple *analyses* of the intervention-outcome relationship?	The analyses presented were consistent with the study objectives, with no evidence of multiple statistical models.	PN
7.3 ... different *subgroups*?	The study included several neuropathic pain conditions, but subgroup analyses were limited and not clearly prespecified, raising some concern regarding selective subgroup reporting.	PY
**Risk of bias judgement**		**Moderate**
Optional: What is the predicted direction of bias due to selection of the reported result?		Favours experimental

Overall bias		
**Risk of bias judgement**		**Serious**
Optional: What is the overall predicted direction of bias for this outcome?	Although the study included a relatively large clinical cohort and used a standardized protocol with validated pain measurements (NRS-11), it was a retrospective, uncontrolled observational study without randomization or a concurrent comparator. The lack of adjustment for baseline confounding, continuation and modification of concomitant analgesic therapies, and unblinded assessment of subjective outcomes resulted in an overall serious risk of bias	Favours experimental

ROBINS-I tool (Stage II): For each study

Study: Ricken et al. (2021) [34]

Specify a target randomized trial specific to the study

Design	Individually randomized
Participants	Adult patients diagnosed with Burning Mouth Syndrome (BMS).
Experimental intervention	Topical capsaicin gel 0.025% applied according to the study protocol with gradual withdrawal over 180 days.
Comparator	Placebo gel/or standard care alone.

Is your aim for this study…?

☐ to assess the effect of *assignment to* intervention

x to assess the effect of *starting and adhering to* intervention

Specify the outcome

Specify which outcome is being assessed for risk of bias (typically from among those earmarked for the Summary of Findings table). Specify whether this is a proposed benefit or harm of intervention.

Reduction in burning intensity measured using a 0–10 subjective burning score (beneficial outcome).<br>Secondary outcome: improvement in oral health-related quality of life assessed by the OHIP-14 questionnaire.

Specify the numerical result being assessed

In case of multiple alternative analyses being presented, specify the numeric result (e.g. RR = 1.52 (95% CI 0.83 to 2.77) and/or a reference (e.g. to a table, figure or paragraph) that uniquely defines the result being assessed.

At 180 days, 6 of 10 patients (60%) reported complete resolution of burning symptoms, whereas the remaining four patients showed stable or reduced symptom scores. OHIP-14 scores improved throughout follow-up in all patients who completed the protocol.

Preliminary consideration of confounders

Complete a row for each important confounding domain (i) listed in the review protocol; and (ii) relevant to the setting of this particular study, or which the study authors identified as potentially important.

"Important" confounding domains are those for which, in the context of this study, adjustment is expected to lead to a clinically important change in the estimated effect of the intervention. "Validity" refers to whether the confounding variable or variables fully measure the domain, while "reliability" refers to the precision of the measurement (more measurement error means less reliability).

**(i) Confounding domains listed in the review protocol**				
Confounding domain	Measured variable(s)	Is there evidence that controlling for this variable was unnecessary?*	Is the confounding domain measured validly and reliably by this variable (or these variables)?	OPTIONAL: Is failure to adjust for this variable (alone) expected to favour the experimental intervention or the comparator?
Baseline burning intensity	Subjective burning score (0–10)	No	Yes	Favour experimental
Baseline severity of BMS	Clinical diagnosis and symptom severity	No	Probably Yes	Favour experimental
Duration of symptoms	Not consistently reported	No	No information	Favour experimental
Previous pain treatment	Medication history	No	Probably Yes	Favour experimental
Age	Years	No	Yes	No information
Sex	Male/Female	No	Yes	No information
Psychological disorders	Psychological history obtained during anamnesis	No	Probably Yes	Favour experimental
Xerostomia	Clinical history	No	Probably Yes	Favour experimental

(ii) Additional confounding domains relevant to the setting of this particular study, or which the study authors identified as important				
Confounding domain	Measured variable(s)	Is there evidence that controlling for this variable was unnecessary?*	Is the confounding domain measured validly and reliably by this variable (or these variables)?	OPTIONAL: Is failure to adjust for this variable (alone) expected to favour the experimental intervention or the comparator?
None identified.			Yes / No / No information	Favour experimental / Favour comparator / No information

* In the context of a particular study, variables can be demonstrated not to be confounders and so not included in the analysis: (a) if they are not predictive of the outcome; (b) if they are not predictive of intervention; or (c) because adjustment makes no or minimal difference to the estimated effect of the primary parameter. Note that “no statistically significant association” is not the same as “not predictive”.

Preliminary consideration of co-interventions

Complete a row for each important co-intervention (i) listed in the review protocol; and (ii) relevant to the setting of this particular study, or which the study authors identified as important.

"Important" co-interventions are those for which, in the context of this study, adjustment is expected to lead to a clinically important change in the estimated effect of the intervention.

**(i) Co-interventions listed in the review protocol**		
Co-intervention	Is there evidence that controlling for this co-intervention was unnecessary (e.g. because it was not administered)?	Is presence of this co-intervention likely to favour outcomes in the experimental intervention or the comparator
Systemic analgesics	No information	Favours experimental
Topical analgesics/anesthetics	No information	Favours experimental
Neuropathic pain medications (e.g., clonazepam, antidepressants)	Partially (continuous users of clonazepam and tricyclic antidepressants were excluded)	No information
Standard supportive oral care	No information	Unpredictable

**(ii) Additional co-interventions relevant to the setting of this particular study, or which the study authors identified as important**		
Co-intervention	Is there evidence that controlling for this co-intervention was unnecessary (e.g. because it was not administered)?	Is presence of this co-intervention likely to favour outcomes in the experimental intervention or the comparator
Medication withdrawal during follow-up	No	Favour experimental
		Favour experimental / Favour comparator / No information
		Favour experimental / Favour comparator / No information
		Favour experimental / Favour comparator / No information

Risk of bias assessment

Responses underlined in green are potential markers for low risk of bias, and responses in red are potential markers for a risk of bias. Where questions relate only to sign posts to other questions, no formatting is used.

Signalling questions	Description	Response options
**Bias due to confounding**		
1.1 Is there potential for confounding of the effect of intervention in this study?<br>**If N/PN to 1.1:** the study can be considered to be at low risk of bias due to confounding and no further signalling questions need be considered	The study lacked a control group and randomization. Baseline symptom severity, psychological factors, xerostomia, previous treatments, and the natural fluctuation of BMS could influence symptom improvement independently of capsaicin.	Y
**If Y/PY to 1.1**: determine whether there is a need to assess time-varying confounding:		
1.2. Was the analysis based on splitting participants' follow up time according to intervention received?<br>**If N/PN**, answer questions relating to baseline confounding (1.4 to 1.6)<br>**If Y/PY**, go to question 1.3.	All participants received the same intervention throughout follow-up.	N
1.3. Were intervention discontinuations or switches likely to be related to factors that are prognostic for the outcome?<br>**If N/PN**, answer questions relating to baseline confounding (1.4 to 1.6)<br>**If Y/PY**, answer questions relating to both baseline and time-varying confounding (1.7 and 1.8)	No switching between intervention groups occurred.	NA

**Questions relating to baseline confounding only**		
1.4. Did the authors use an appropriate analysis method that controlled for all the important confounding domains?	No statistical adjustment for confounding variables was performed.	N
1.5. **If Y/PY to 1.4**: Were confounding domains that were controlled for measured validly and reliably by the variables available in this study?		NA
1.6. Did the authors control for any post-intervention variables that could have been affected by the intervention?	No adjustment for post-intervention variables was reported.	N
**Questions relating to baseline and time-varying confounding**		
1.7. Did the authors use an appropriate analysis method that controlled for all the important confounding domains and for time-varying confounding?	The analysis was not based on changes in intervention status over time. All participants received the same intervention throughout the study, and no time-varying confounding was present	N
1.8. **If Y/PY to 1.7**: Were confounding domains that were controlled for measured validly and reliably by the variables available in this study?		NA
**Risk of bias judgement**		**Serious**
Optional: What is the predicted direction of bias due to confounding?		Favours experimental

Bias in selection of participants into the study		
2.1. Was selection of participants into the study (or into the analysis) based on participant characteristics observed after the start of intervention?<br>**If N/PN to 2.1:** go to 2.4	Patients were enrolled before treatment based on the diagnosis of BMS.	PN
2.2. **If Y/PY to 2.1**: Were the post-intervention variables that influenced selection likely to be associated with intervention?		NA
2.3 **If Y/PY to 2.2**: Were the post-intervention variables that influenced selection likely to be influenced by the outcome or a cause of the outcome?		NA
2.4. Do start of follow-up and start of intervention coincide for most participants?	Baseline assessments were performed before initiating topical capsaicin treatment.	Y
2.5. **If Y/PY to 2.2 and 2.3, or N/PN to 2.4**: Were adjustment techniques used that are likely to correct for the presence of selection biases?		NA
**Risk of bias judgement**		Low
Optional: What is the predicted direction of bias due to selection of participants into the study?		Favours experimental / Favours comparator / Towards null /Away from null / Unpredictable

Bias in classification of interventions		
3.1 Were intervention groups clearly defined?	The capsaicin concentration (0.025%), application protocol, and treatment duration were clearly described.	Y
3.2 Was the information used to define intervention groups recorded at the start of the intervention?	Treatment exposure was established before follow-up evaluations.	Y
3.3 Could classification of intervention status have been affected by knowledge of the outcome or risk of the outcome?	Intervention classification occurred before outcome measurement.	N
**Risk of bias judgement**		**Low**
Optional: What is the predicted direction of bias due to classification of interventions?		Favours experimental / Favours comparator / Towards null /Away from null / Unpredictable

Bias due to deviations from intended interventions		
**If your aim for this study is to assess the effect of assignment to intervention, answer questions 4.1 and 4.2**		
4.1. Were there deviations from the intended intervention beyond what would be expected in usual practice?		NA
4.2. **If Y/PY to 4.1**: Were these deviations from intended intervention unbalanced between groups *and* likely to have affected the outcome?		NA
**If your aim for this study is to assess the effect of starting and adhering to intervention, answer questions 4.3 to 4.6**		
4.3. Were important co-interventions balanced across intervention groups?	There was no comparison group, and medication withdrawal during follow-up may have influenced outcomes.	N
4.4. Was the intervention implemented successfully for most participants?	Most participants followed the protocol, although several did not complete the 180-day follow-up.	PY
4.5. Did study participants adhere to the assigned intervention regimen?	Adherence appeared acceptable among completers, but it was not objectively monitored.	PY
4.6. **If N/PN to 4.3, 4.4 or 4.5**: Was an appropriate analysis used to estimate the effect of starting and adhering to the intervention?	No analysis accounting for protocol deviations or adherence was performed.	N
**Risk of bias judgement**		**Moderate**
Optional: What is the predicted direction of bias due to deviations from the intended interventions?		Favours experimental

Bias due to missing data		
5.1 Were outcome data available for all, or nearly all, participants?	Only 10 of the 18 enrolled patients completed the treatment protocol and were included in the final analysis, indicating substantial loss to follow-up.	N
5.2 Were participants excluded due to missing data on intervention status?	No participants were excluded because of missing information regarding the intervention received.	N
5.3 Were participants excluded due to missing data on other variables needed for the analysis?	Exclusions were related to non-completion rather than missing baseline variables.	N
5.4 **If PN/N to 5.1, or Y/PY to 5.2 or 5.3**: Are the proportion of participants and reasons for missing data similar across interventions?	Not applicable because there was only one intervention group.	NA
5.5 **If PN/N to 5.1, or Y/PY to 5.2 or 5.3**: Is there evidence that results were robust to the presence of missing data?	No sensitivity analyses were performed to assess the impact of missing data.	N
**Risk of bias judgement**		**Serious**
Optional: What is the predicted direction of bias due to missing data?		Favours experimental

Bias in measurement of outcomes		
6.1 Could the outcome measure have been influenced by knowledge of the intervention received?	Burning intensity and quality of life were self-reported outcomes, and participants were aware that they had received active treatment.	Y
6.2 Were outcome assessors aware of the intervention received by study participants?	Neither participants nor investigators were blinded.	Y
6.3 Were the methods of outcome assessment comparable across intervention groups?	Only one intervention group was evaluated.	N
6.4 Were any systematic errors in measurement of the outcome related to intervention received?	Subjective outcome assessment without blinding increases the likelihood of expectation and placebo effects.	PY
**Risk of bias judgement**		**Serious**
Optional: What is the predicted direction of bias due to measurement of outcomes?		Favours experimental

Bias in selection of the reported result		
Is the reported effect estimate likely to be selected, on the basis of the results, from...		
7.1. ... multiple outcome *measurements* within the outcome domain?	The protocol clearly specified the burning score and OHIP-14 as the main outcomes, and these were consistently reported at all planned follow-up visits.	PN
7.2 ... multiple *analyses* of the intervention-outcome relationship?	The analyses were consistent with the study objectives, with no indication that multiple statistical approaches were tested and selectively reported.	PN
7.3 ... different *subgroups*?	No subgroup analyses were reported or emphasized.	PN
**Risk of bias judgement**		**Low**
Optional: What is the predicted direction of bias due to selection of the reported result?		Unpredictable

Overall bias		
**Risk of bias judgement**		**Serious**
Optional: What is the overall predicted direction of bias for this outcome?		Favours experimental

This work is licensed under a Creative Commons Attribution-NonCommercial-NoDerivatives 4.0 International License.
