# Supplementary material for: Capsaicin for Orofacial Pain Management: Systematic Review and Meta-Analysis
Source: Int J Mol Sci. 2026 Aug 7;27(16):7099. doi: 10.3390/ijms27167099 (PMC13513309; doi:10.3390/ijms27167099)
Supplement: Supplementary file 1 [file ijms-27-07099-s001.zip › Supplementary File S5.pdf]

SUPPLEMENTARY FILES

Supplementary File S5. Summary of clinicodemographic data, intervention and outcomes of the use of capsaicin for the treatment of orofacial pain.

Author (year)	Country	Study	Study duration	Subgroup of orofacial pain (site)	Participants (n)	Mean age (years) (SD)	Sex	Interventions group		Capsaicin concentra-tion used	Outcome (PS, BS, VAS, QoL)			Administration route	Adverse effects
								Capsaicin	control		Baseline (mean ± SD)	After treatment (mean ± SD)	% reduction		
**Berger et al. (1995) [33]**	USA	Pilot clinical trial (uncontrolled)	-	oral mucositis	11	54 (19.1)	F, 6; M, 5	11	0	The taffy recipe (454 g) contains approximately 0.005-0.008 g capsaicin (approximately equivalent to 5-9 ppm).	**PS**[¥] 6.7 (2)	**PS**[¥] 1.9 (1.7)	71.6	Topical/oral systemic	Nausea, and a participant with a prior history of hemorrhoids developed burning hemorrhoids.
**Campbell et al. (2017) [28]**	USA	Clinical trial	May 2006 to Jan 2009	Temporomandibular joint disorders (TMDs)	15	27.4 (11.9)	F, 15	7	8	8% capsaicin	**VAS***: Vehicle: 3.7 (0.8) Capsaicin: 2.7 (0.7)	**VAS***: Vehicle: 2 (0.8) Capsaicin: 0.9 (0.6)	Vehicle: 45.9 Capsaicin: 66.7	Topical	Two patients (1 from each of the TMD and non-TMD capsaicin groups) requested the cream to be removed before the 2-h time limit after drug application due to excessive pain, and their data were not included in the results.
**Fusco et al. (1992) [29]**	Italy	Cross-sectional study	-	idiopathic trigeminal neuralgia	12	65.4 (3.2)	F, 7; M, 5	12	0	0.05% capsaicin	**BS** (0-4)* 4 (0)	**BS** 3.5 (0.5)	BS 12.5 PS 4.6	Topical	NI

											**PS** (0-10)** 8.7 (0.9)	**PS** 8.3 (0.8)			
**Gonçalves et al. (2020) [37]**	Brazil	Retrospective observational study	Feb. 2011 and Feb. 2019	Peripheral neuropathic pain - trigeminal neuralgia	4	56.0 (26.6)	F, 4; M, 0	4	0	8% capsaicin patch	**NRS-11**[$]<br>7.3 (3.2)	**NRS-11**[$]<br>3.5 (0.6)	52.1	Topical	20.8% of participants increased their pain one hour after 8% capsaicin patch application. There was only one case of need for early adhesive removal due to pain intolerance. In the patients included in the sample, no major treatment-related adverse reactions were reported.
**Jorgensen et al. (2016) [25]**	Denmark	Cross-over clinical trial	Feb. 2009 to May 2010	Burning mouth syndrome (BMS)	22	61 (-)	F, 22	9	10	0.01% and 0.025% capsaicin	**VAS**[#]<br>Capsaicin: 6.4 (2.0) [1a time point]<br>**VAS**[#]<br>Capsaicin: 5.9 (3.2) [2a time point]	**VAS**[#]<br>Capsaicin: 4.7 (2.7) [1a time point]<br>**VAS**[#]<br>Capsaicin: 5.1 (3.0) [2a time point]	Capsaicin: 26.6 [1a time point]<br>Capsaicin: 13.6 [2a time point]	Topical	Three patients stopped therapy during the first week of treatment due to gastrointestinal side effects such as nausea and itching and another found the consistency of the gel

															unpleasant
								10	9	0.025% and 0.01% capsaicin	**VAS**[#]<br><br>Capsaicin: 4.6 (2.6) [1a time point]<br><br>**VAS**[#]<br><br>Capsaicin: 3.5 (2.8) [2a time point]	**VAS**[#]<br><br>Capsaicin: 3.6 (2.8) [1a time point]<br><br>**VAS**[#]<br><br>Capsaicin: 3.0 (2.9) [2a time point]	Capsaicin: 21.7 [1a time point]<br><br>Capsaicin: 14.3 [2a time point]		
**Petruzzi et al. (2004) [36]**	Italy	Clinical trial	-	Burning mouth syndrome (BMS)	50	56.5 (6.5)	F, 36; M, 14	25	25	Oral administration of 0.25% capsaicin	**VAS**[$] Scores, n<br>Vehicle:<br>8-10, 13<br>4-7, 7<br>0-3, 5<br><br>Capsaicin:<br>8-10, 15<br>4-7, 8<br>0-3, 2	**VAS**[$] Scores, n<br>Vehicle:<br>8-10, 13<br>4-7, 6<br>0-3, 6<br><br>Capsaicin:<br>8-10, 1<br>4-7, 3<br>0-3, 21	VAS[$] Scores<br>Vehicle:<br>8-10, 0<br>4-7, 14.3<br>0-3, NA<br><br>Capsaicin:<br>8-10, 93.3<br>4-7, 62.5<br>0-3, NA	Oral (Systemic)	Gastric pain
**Ricken et al. (2021) [34]**	Brazil	NI	2017 to 2019	Burning mouth syndrome (BMS)	18	-	F, 18; M, 0	10	0	0.025% capsaicin	**Burning scale**[Ω] 7.4 (1.8)<br><br>**OHIP-14** 4.3 (1.1)	**Burning scale** [Ω] 4.3 (2.5)<br><br>**OHIP-14** 2.9 (1.6)	Burning scale 41.9<br><br>OHIP-14 32.6	Topical	erythema on the lip and jugal mucosa
**Wagner et al. (2013) [35]**	Germany	Retrospective observacional study	Jan. 2010 to Feb. 2011	Neuropathic pain - face	2	-	-	2	0	capsaicin 8% patch	NPRS[†] All patients: 7.5	NPRS[†] All patients: 4.3	All patients: 48 Neuropathy,	Topical	discomfort during their first capsaicin

													Face: 59%		patch application.
**Winocur et al. (2000) [27]**	Israel	Clinical trial	-	temporomandibular joint disorders (TMDs)	30	36.7 (15.2)	F, 24; M, 6	17	13	0.025% capsaicin.<br><br>Improvement was attributed mainly to the passage of time and a strong placebo effect, rather than the specific action of capsaicin.	**VAS**<br>Vehicle: 6.7<br><br>Capsaicin: 7.3	**VAS**<br>Vehicle: 4<br><br>Capsaicin: 3.4	Vehicle: 40.3<br>Capsaicin: 53.4	Topical	warm sensation, stinging, burning, or redness.

Legenda: NA, not applicable. NI, not informed. VAS, visual analogue scale. ¥Pain severity using a numeric verbal scale of 0 to 10 (0 = no pain and 10 = being the most intense pain ever experienced). *Burning sensation was scored using a numeric rating scale of 0 to 4. **Pain severity was scored using a numeric verbal scale of 0 to 10 (0 = no pain, 2 = very mild, 4 = mild, 6 = moderate, 8 = severe, 10 = very severe).#severity of oral burning pain on a VAS (0 = no pain and 10 = worst imaginable pain). $VAS expressed in score. ΩBurning scale, subjective score of intensity of the burning ranging from 0 to 10 (0 = symptom-free, 5 = bearable symptoms and 10 = unbearable burning). 30 days after treatment measuring. †NPRS, 11-point numeric pain rating scale. $ Numeric Rating Scale 0-10, baseline and after 15 d.
