# Supplementary material for: Capsaicin for Orofacial Pain Management: Systematic Review and Meta-Analysis
Source: Int J Mol Sci. 2026 Aug 7;27(16):7099. doi: 10.3390/ijms27167099 (PMC13513309; doi:10.3390/ijms27167099)
Supplement: Supplementary file 1 [file ijms-27-07099-s001.zip › Supplementary File S6.pdf]

SUPPLEMENTARY FILES

Supplementary File S6. The certainty of the evidence was assessed using the GRADE approach

Author(s): Ana Claudia de Macedo Andrade, Fernanda Aragão Felix, Sebastián Brauchi and Bruna Benso*

Question: Capsaicin compared to Placebo or Capsaicin in a low dose for Orofacial pain

Setting:

Bibliography:

Certainty assessment							№ of patients		Effect		Certainty	Importance
№ of studies	Study design	Risk of bias	Inconsistency	Indirectness	Imprecision	Other considerations	Capsaicin	Placebo or Capsaicin in a low dose	Relative (95% CI)	Absolute (95% CI)		
**Orofacial Pain Reduction (capsaicin vs control) (assessed with: Visual Analog Scale (VAS; 0-10))**												
4	randomised trials	serious[a]	serious[b]	not serious	serious[c]	none	66	65	-	MD **1.1 points (0-10) lower** (2.79 lower to 0.58 higher)	⊕◯◯◯ Very low[a,b,c]	CRITICAL
**Orofacial Pain Reduction (before-and-after studies) (assessed with: Visual scale (0-10))**												
3	non-randomised studies	serious[d]	not serious	not serious	serious[e]	none	26		-	MD **6.39 points (0-10) lower** (5.38 lower to 7.4 lower)	⊕⊕◯◯ Low[d,e]	CRITICAL
**Capsaicin-related adverse events (follow-up: range 7 days to 30 days; assessed with: Number of participants experiencing adverse events)**												
3	randomised trials	serious[f]	not serious	not serious	not serious	none	22/48 (45.8%)	2/46 (4.3%)	**OR 19.84** (4.32 to 91.21)	**431 more per 1000** (from 121 more to 762 more)	⊕⊕⊕◯ Moderate[f]	CRITICAL
								4.3%		**428 more per 1000** (from 120 more to 761 more)		

CI: confidence interval; **MD:** mean difference; **OR:** odds ratio

Explanations

a. Two of the four randomized controlled trials were judged to be at high risk of bias according to the RoB 2 tool, while the remaining two studies presented some concerns. No study was judged to be at low risk of bias overall, raising concerns that methodological limitations may have influenced the estimated treatment effect.

b. The evidence was downgraded for inconsistency because the forest plot showed substantial heterogeneity ($I^2 > 70\%$).

c. The confidente interval is wide and includes effects ranging from a clinically important reduction close to no perfect. In addition, the total sample size is small, increasing uncertainty in the estimate.

d. Most studies were judged as having serious risk of bias according to ROBINS-I due to lack of a control group, absence of adjustment for confounding, and subjective outcome assessment.

e. The pooled sample size was small ($n = 26$), and the optimal information size was not achieved despite a statistically significant pooled effect.

f. Two of the four randomized controlled trials were judged to be at high risk of bias according to the RoB 2 tool, while the remaining two studies presented some concerns. No study was judged to be at low risk of bias overall, raising concerns that methodological limitations may have influenced the estimated treatment effect.
